# Incidence Trends of Five Common Sexually Transmitted Infections Excluding HIV From 1990 to 2019 at the Global, Regional, and National Levels: Results From the Global Burden of Disease Study 2019

**DOI:** 10.3389/fmed.2022.851635

**Published:** 2022-03-02

**Authors:** Leiwen Fu, Yinghui Sun, Min Han, Bingyi Wang, Fei Xiao, Yiguo Zhou, Yanxiao Gao, Thomas Fitzpatrick, Tanwei Yuan, Peiyang Li, Yuewei Zhan, Yong Lu, Ganfeng Luo, Junyi Duan, Zhongsi Hong, Christopher K. Fairley, Tong Zhang, Jin Zhao, Huachun Zou

**Affiliations:** ^1^School of Public Health (Shenzhen), Sun Yat-sen University, Shenzhen, China; ^2^Department of Medical Administration, Guangdong Provincial People's Hospital, Guangdong Academy of Medical Sciences, Guangzhou, China; ^3^The Fifth Affiliated Hospital of Sun Yat-sen University, Zhuhai, China; ^4^Department of Internal Medicine, University of Washington, Seattle, WA, United States; ^5^School of Public Health, The Key Laboratory of Environmental Pollution Monitoring and Disease Control, Ministry of Education, Guizhou Medical University, Guiyang, China; ^6^Clinical and Research Center for Infectious Diseases, Beijing Youan Hospital, Capital Medical University, Beijing, China; ^7^Central Clinical School, Monash University, Melbourne, VIC, Australia; ^8^Melbourne Sexual Health Centre, Alfred Health, Melbourne, VIC, Australia; ^9^Department of HIV/AIDS Control and Prevention, Shenzhen Center for Disease Control and Prevention, Shenzhen, China; ^10^Kirby Institute, University of New South Wales, Sydney, NSW, Australia

**Keywords:** global burden, STIs, syphilis, chlamydia, gonorrhea, trichomoniasis, genital herpes

## Abstract

**Objective:**

Sexually transmitted infections (STIs) are common worldwide and pose a challenge to public health. We conducted this study to assess the annual incidence of five common STIs, including syphilis, chlamydia, gonorrhea, trichomoniasis, and genital herpes at the global, regional, and national levels.

**Materials and Methods:**

We obtained detailed data on STIs excluding HIV from 1990 to 2019 from the Global Burden of Disease (GBD) 2019 database. Estimated annual percentage change (EAPC) was calculated to quantify trends in age-standardized incidence rates (ASR) of STIs, stratified by gender, sociodemographic index (SDI) region, and pathogenic microorganism.

**Results:**

Globally, incident cases of STIs increased by 58.15% from 486.77 million in 1990 to 769.85 million in 2019, but the annual change in ASR was only −0.04% (95% CI −0.09 to 0.01) per year. EAPC was 0.16 (0.06 to 0.26) for syphilis, 0.09 (0.05 to 0.13) for genital herpes, 0.06 (0.03 to 0.09) for trichomoniasis, −0.21 (−0.36 to −0.06) for chlamydia, and −0.14 (−0.19 to −0.08) for gonorrhea. High SDI regions reported significant increases in ASR of syphilis and chlamydia.

**Conclusions:**

The burden of disease from STIs remains large, though control of STIs has contributed to the decreasing incidence in most regions, especially in the low-SDI regions. Globally, over the past 20 years, the ASR has remained stable for trichomoniasis and genital herpes decreased for chlamydia and gonorrhea, and increased for syphilis.

## Introduction

Sexually transmitted infections (STIs) are among the most common infectious diseases reported worldwide (1). More than one million people are newly infected with STIs per day (2). In 2016, the World Health Organization (WHO) estimated there were 376 million new infections of four curable STIs, including chlamydia (127 million), gonorrhea (87 million), syphilis (6 million), and trichomoniasis (156 million), respectively (2–4). Additionally, more than 500 million people are living with genital herpes (2). Although most STIs are usually not fatal; they result in a substantial burden of diseases (1). STIs such as herpes and syphilis are associated with an increased risk of HIV transmission (3, 5). STIs, including chlamydia and gonorrhea can result in reproductive tract morbidities, such as infertility and pelvic inflammatory diseases among women (6–8). Mother-to-child transmission of STIs can result in neonatal death, congenital deformities, and other adverse birth outcomes (3, 5, 9, 10). A report published by the United States Centers for Disease Control and Prevention in 2016 warned that high levels of antibiotic resistance in the USA might soon make gonorrhea untreatable, and a similar trend is now emerging in chlamydia (3, 11, 12). Although the incidence of STIs is high, prevention efforts targeting STIs have been largely ignored because safe-sex messaging related to HIV is assumed to be sufficient to control STIs transmission (11, 13). Most STIs are asymptomatic and therefore easily neglected (14, 15). Changes in sexual behaviors due to increasing availability of HIV pre-exposure prophylaxis, including decreased condom use and increased number of sexual partners, may led to a significant increase in STIs (16).

To date, there have been limited population estimates of the burden of STIs in different regions around the world (4). Two systematic reviews of estimates of STIs in 2012 and 2016 reported the global prevalence and incidence of chlamydia, gonorrhea, trichomoniasis, and syphilis in adults remained high; nearly one million new infections with curable STIs were detected every day, but varied by region and gender (4, 17). Estimates of the incidence of specific STIs at the regional level, such as herpes simplex virus and congenital syphilis, show that despite the decrease, the prevalence remains stable and poses a huge global burden (18). Previous studies have focused on cross-sectional snapshots of STI burdens in specific regions and populations but have not reported distribution patterns or trends over time. Existing monitoring data on STIs are scattered, and systematic monitoring and comparison at the national level are needed to inform the rational allocation of health resources (3).

The Global Burden of Disease Study (GBD) 2019 is a systematic effort to assess the burden of many diseases according to age, gender, and geography across the world from 1990 to 2019 (19). STIs excluding HIV have been estimated and classified into six categories: syphilis, chlamydia, gonorrhea, trichomoniasis, genital herpes, and other STIs. No previous study has quantified annual trends in the incidence of STIs, excluding HIV and specific pathogenic microorganisms over a specified time period. The first strategic direction of the WHO Global Health Sector Strategy on Sexually Transmitted Infections 2016–2021 is to collect information on STI prevalence and incidence across representative populations (20). Therefore, understanding the incidence of STIs in regions and countries is essential to advocating, funding, planning, and implementing the prevention and control of STIs. In the present study, we used the results of the GBD 2019 to estimate the burden of syphilis, chlamydia, gonorrhea, trichomoniasis, and genital herpes by determining temporal trends in incidence at the global, regional, and national levels.

## Materials and Methods

### Data Source

We extracted data from the GBD 2019 using the Global Health Data Exchange query tool (http://ghdx.healthdata.org/gbd-results-tool). The GBD 2019 reports estimates of incidence, prevalence, mortality, years of life lost (YLLs), years lived with disability (YLDs), and disability-adjusted life-years (DALYs) due to 369 diseases and injuries, for two genders as well as for 204 countries and territories. Incident cases, incidence rates, and age-standardized incidence rates (ASR) of syphilis, chlamydia, gonorrhea, trichomoniasis, and genital herpes from 1990 to 2019, segregated by gender, age, location, and specific STI, were extracted using GBD's operation guide (19, 21). A Bayesian meta-regression modeling tool, DisMod-MR 2.1, was used to ensure consistency between incidence, prevalence, remission, excess mortality, and cause-specific mortality for most causes. The estimated incidence of trichomoniasis, genital herpes, syphilis; each in separate models in DisMod-MR 2.1. The incidence of chlamydia and gonorrhea was estimated in a custom process outside of Dismod, as described in a previous study (19). Available data were collected from 21 GBD regions in terms of geography, e.g., East Asia (Table 1), and 204 countries and territories. Age was extracted by five-year age groups for a total of 20 GBD age groups. Because the GBD database groups the age according to the interval of 5 years, it is divided into 1–4, 5–9, etc. In order to facilitate the comparison between different age groups, we did not include people under the age of one year in this study. The 204 countries and territories were categorized into five regions based on the sociodemographic index (SDI): low, low-middle, middle, high-middle, and high SDI regions. The SDI is a summary measure that estimates a location's position on a spectrum of development, which is a composite indicator of a country's lag-distributed income per capita, average years of schooling, and the fertility rate in females under the age of 25 years. The cutoff values used to determine quintiles for analysis were then computed using country-level estimates of SDI for 2019. Additional details on results from the SDI calculation are available in the GBD2019. The general methods for the GBD 2019 have been detailed in previous studies (19). Data collection for syphilis from case notification, ante-natal and community surveillance data, cross-sectional studies and claims data; for chlamydia and gonorrhea from case notification, ante-natal and community surveillance data and cross-sectional studies; for trichomoniasis from case notification and cross-sectional studies; for genital herpes from cross-sectional studies. Case definitions for all of these infections were based on laboratory findings, except late syphilis, which was ascertained from administrative data using ICD-9.093–095 and ICD-10A52 and I98.0. For chlamydia, gonorrhea, and trichomoniasis, the reference case definition was diagnosis with a nucleic acid amplification test (NAAT). For all STIs, sources were excluded if the sample population was drawn exclusively from a high-risk group (e.g., HIV-positive, men who have sex with men [MSM], or sex workers).

**Table 1:** Incident cases and age-standardized incidence rates of sexually transmitted infections in 1990 and 2019 and estimated annual percentage change from 1990 to 2019.

**Characteristics**	**1990**		**2019**		**1990–2019**
	**Incident cases** **No. ×10^**3**^ (95% UI)**	**ASR per 100,000** **No. (95% UI)**	**Incident cases** **No. ×10^**3**^ (95% UI)**	**ASR per 100,000** **No. (95% UI)**	**EAPC** **No. (95% CI)**
Overall	486,771.20 (416,757.41–565,524.92)	9,323.71 (7,994.18–10,804.75)	769,851.91 (659,059.33–892,663.79)	9,535.71 (8,169.73–11,054.76)	−0.04 (−0.09 to 0.01)
Sex					
Male	271,108.35 (230,667.77–316,253.28)	10,369.15 (8,814.94–12,091.21)	426,072.07 (362,331.77–496,890.04)	10,471.63 (8,892.20–12,176.10)	−0.04 (−0.09 to 0.02)
Female	215,662.85 (185,370.23–250,535.94)	8,262.58 (7,107.40–9,594.72)	343,779.83 (293,773.14–399,266.98)	8,602.40 (7,358.00–10,001.18)	−0.04 (−0.10 to 0.02)
SDI category					
Low-SDI	50,472.93 (43,231.14–58,660.96)	11,817.48 (10,145.19–13,761.86)	107,712.82 (91,841.64–125,724.84)	10,933.50 (9,332.30–12,773.10)	−0.09 (−0.17 to −0.02)
Low-middle-SDI	89,265.65 (76,517.23–103,797.10)	8,906.49 (7649.99–10,325.71)	160,814.99 (137,884.69–186,617.47)	8,883.31 (7,608.08–10,298.21)	−0.05 (−0.08 to −0.03)
Middle-SDI	181,587.51 (155,220.87–211,757.46)	10,830.67 (9,263.26–12,636.21)	264,896.29 (226,459.24–309,655.39)	10,085.26 (8,646.52–11,806.73)	−0.15 (−0.23 to −0.08)
High-middle-SDI	112,059.93 (95,563.15–130,936.37)	9,238.83 (7,913.02–10,785.43)	148,390.88 (126,213.58–173,312.20)	9,222.42 (7,864.23–10,753.14)	−0.20 (−0.29 to −0.11)
High-SDI	53,073.66 (44,435.87–62,521.66)	5,940.18 (5,004.14–6,988.20)	65,804.44 (55,027.33–77,434.49)	6,117.57 (5,136.38–7,209.63)	0.06 (0.05 to 0.08)
Sexually transmitted infections excluding HIV					
Syphilis	8,845.22 (6,562.51–11,588.86)	160.03 (120.66–208.10)	14,114.11 (10,648.49–18,415.97)	178.48 (134.94–232.34)	0.16 (0.06 to 0.26)
Chlamydia	151,695.68 (113,998.56–199,144.01)	2,867.67 (2,150.60–3,741.43)	232,534.84 (174,269.19–303,009.11)	2,883.87 (2,161.21–3,762.80)	−0.21 (−0.36 to −0.06)
Gonorrhea	67,732.22 (51,820.12–89,251.61)	1,178.58 (912.29–1,536.00)	87,951.95 (68,461.02–112,961.84)	1,124.39 (872.97–1,441.08)	−0.14 (−0.19 to −0.08)
Trichomoniasis	205,446.49 (151,261.12–273,107.88)	4,157.14 (3,061.97–5,439.34)	354,466.58 (260,117.34–461,359.68)	4,327.29 (3,176.53–5,645.76)	0.06 (0.03 to 0.09)
Genital herpes	53,051.59 (45,029.38–61,934.04)	960.29 (822.81–1,116.81)	80,784.43 (68,810.96–94,200.33)	1,021.68 (869.15–1,191.20)	0.09 (0.05 to 0.13)
Region					
East Asia	137,725.48 (116,644.99–162,625.37)	10,607.48 (8,996.37–12,490.97)	178,904.41 (150,660.79–211,566.79)	10,476.70 (8,849.86–12,376.60)	−0.41 (−0.57 to −0.24)
Southeast Asia	49,962.40 (42,687.23–58,572.42)	11,326.64 (9,713.89–13,174.51)	82,549.05 (70,604.05–96,081.74)	11,230.92 (9,620.76–13,068.46)	−0.03 (−0.04 to −0.03)
Oceania	871.37 (746.46–1,014.18)	14,588.13 (125,49.89–16,873.11)	1,862.95 (1,576.67–2,184.08)	14,285.59 (12,118.18–16,679.43)	0.01 (−0.03 to 0.04)
Central Asia	8,196.70 (6,894.45–9,713.17)	12,383.96 (10,497.47–14,530.25)	12,178.68 (10,273.85–14,353.30)	12,235.85 (10,365.83–14,354.40)	−0.10 (−0.12 to −0.08)
Central Europe	11,629.25 (9,869.73–13,512.87)	9,173.26 (78,07.47–10,696.18)	10,679.92 (9,118.03–12,422.30)	9,126.48 (7,756.82–10,664.06)	−0.04 (−0.05 to −0.03)
Eastern Europe	23,434.74 (20,089.42–27,523.04)	9,909.11 (8,510.07–11,739.04)	21,459.59 (18,307.99–25,324.18)	9,895.41 (8,480.04–11,724.59)	−0.06 (−0.07 to −0.04)
High-income Asia Pacific	11,073.03 (9,351.29–13,025.79)	5,780.52 (4,877.69–6,788.02)	11,164.35 (9,321.58–13,155.00)	5,705.97 (4,794.87–6,714.58)	−0.02 (−0.04 to 0.00)
Australasia	1,143.33 (970.08–1,346.40)	5,247.11 (4,465.01–6,157.35)	1,521.33 (1,288.04–1,781.82)	5,018.90 (4,251.70–5,907.28)	−0.14 (−0.24 to −0.05)
Western Europe	15,705.13 (13,112.74–18,669.09)	3,768.35 (3,139.71–4,497.39)	17,190.14 (14,251.86–20,319.64)	3,729.40 (3,099.27–4,444.56)	−0.03 (−0.05 to −0.01)
Southern Latin America	2,747.36 (2367.56–3,189.59)	5,654.43 (4,850.87–6,571.61)	3,990.25 (3,429.68–4,646.06)	5,51.23 (4,849.22–6,583.30)	−0.01 (−0.02 to 0.01)
High-income North America	20,552.26 (16,813.62–24,896.24)	6,692.36 (5,494.37–8,065.15)	24,479.62 (20,010.26–29,207.73)	6,489.07 (5,320.69–7,797.60)	−0.24 (−0.28 to −0.20)
Caribbean	4,325.39 (3,700.06–5,029.65)	12,460.65 (10,649.93–14,467.67)	6,136.82 (5,240.39–7,115.89)	12,540.87 (10,720.36–14,571.42)	−0.01 (−0.02 to 0.01)
Andean Latin America	3,163.54 (2,712.04–3,676.01)	9,278.14 (7,938.01–10,763.57)	6,028.75 (5,141.35–7,050.07)	9,213.75 (7,866.87–107,48.10)	0.00 (−0.02 to 0.01)
Central Latin America	18,546.46 (15,786.31–21,785.53)	12,590.93 (10,713.35–14,664.66)	33,539.52 (28,375.32–39,254.34)	12,756.44 (10,798.14–14,937.03)	0.11 (0.08 to 0.14)
Tropical Latin America	18,910.45 (16,142.13–22,082.28)	12,809.55 (10,978.92–14,976.17)	32,029.05 (27,292.76–37,417.34)	12,955.71 (11,074.32–15,125.80)	−0.06 (−0.12 to 0.00)
North Africa and Middle East	28,845.03 (24,682.92–33,559.92)	9,552.82 (8,178.98–11,091.56)	58,679.34 (49,671.26–68,405.06)	8,946.10 (7,619.55–10,371.37)	−0.20 (−0.22 to −0.18)
South Asia	67,172.26 (57,095.03–79,685.03)	6,818.42 (5,796.52–8,055.92)	125,287.68 (106,806.37–147,313.92)	6,652.49 (5,662.30–7,819.84)	−0.11 (−0.13 to −0.09)
Central Sub-Saharan Africa	5,650.89 (4,931.90–6,496.61)	12,289.81 (10,723.69–14,164.84)	14,097.74 (12,225.69–16,294.45)	12,093.61 (10,531.43–13,910.25)	−0.07 (−0.09 to −0.05)
Eastern Sub-Saharan Africa	26,026.33 (22,217.09–30,528.73)	17,597.87 (14,939.06–20,625.45)	59,742.73 (50,627.16–70,681.29)	17,033.30 (14,330.44–20,087.35)	−0.22 (−0.27 to −0.18)
Southern Sub-Saharan Africa	10,475.91 (9,095.70–12,113.61)	21,090.11 (18,370.38–24,194.60)	16,722.29 (14,470.90–19,371.69)	19,973.12 (17,382.69–23,001.57)	−0.26 (−0.32 to −0.20)
Western Sub-Saharan Africa	20,613.89 (17,656.99–24,123.30)	13,319.18 (11,355.57–15,603.23)	51,607.69 (43,835.11–60,708.46)	13,358.91 (11,298.50–15,721.57)	−0.01 (−0.06 to 0.03)

*UI, uncertainty interval; ASR, age-standardized incidence rate; CI, confidential interval; EAPC, estimated annual percentage change*.

For all STIs excluding genital herpes, the datasets were supplemented with a manual search of national ministry of health websites, antenatal clinic surveillance reports, data from the GBD collaborator network, and case-notification data from locations where centralized reporting was mandatory. The genital herpes dataset was only supplemented by sources from the GBD collaborator network. With regard to specific STIs, details on the flow chart, definitions, input data, and modeling strategy are available in the online Supplementary Appendix 1 of the GBD 2019 (https://www.ncbi.nlm.nih.gov/pmc/articles/PMC7567026/bin/mmc1.pdf) (19). The human development index (HDI) values of all nations were collected from the World Bank (http://hdr.undp.org/en/composite/trends).

### Statistical Analysis

Incident cases of all STIs from 1990 to 2019 were summarized. According to the previous study, the estimated annual percentage change (EAPC) is a summary and widely used measure of the ASR trend over a specified interval. ASR and EAPC were calculated to estimate trends of STI incidence (22). ASR (per 100,000 persons) was calculated using the following formula:


$$ASR=\frac{\sum_{i=1}^{N}\alpha_{i}w_{i}}{\sum_{i=1}^{N}w_{i}}$$

Where α_i_ and *w*_*i*_ represent the age-specific rate and number of people (or the weight) in the *i*^*th*^ age group, respectively. *N* represents the number of age groups. The 95% uncertainty interval (UI) was generated from 2.5% and 97.5% quantiles extracted 1,000 times from the posterior distribution. To summarize ASR trends over a specified interval, EAPC and its 95% confidence interval (CI) were calculated using the following linear regression model (23, 24):


$$\begin{array}{l} \text{ y}=\alpha +\beta x+\varepsilon \\ EAPC=100\times (e^{\beta }-1) \end{array}$$

where y = ln (ASR), and x = calendar year.

An ASR was determined to represent a trend of increasing or decreasing incidence over time if both the EAPC and its 95% CI was above or below 0, respectively. To explore factors that may influence EAPC, correlation analyses were conducted comparing EAPC and ASR (1990), and HDI (2019), respectively, for each included STI. The global, regional, and national incidence rates of syphilis, chlamydia, gonorrhea, trichomoniasis, and genital herpes were described using maps, including ASR in 2019, the percentage change in incident cases, and EAPC in ASR from 1990 to 2019. Correlation analysis was used to estimate the ρ indices and *p* values for the association of EAPC with HDI and baseline ASR. The breakpoint was estimated by the change of ρ indices in the smoothed curve. All data were analyzed using R software 3.6.0 (R Core Team, Vienna, Austria). A *P* value < 0.05 was considered statistically significant.

## Results

### Incident Cases of STIs

**Figure 1:**
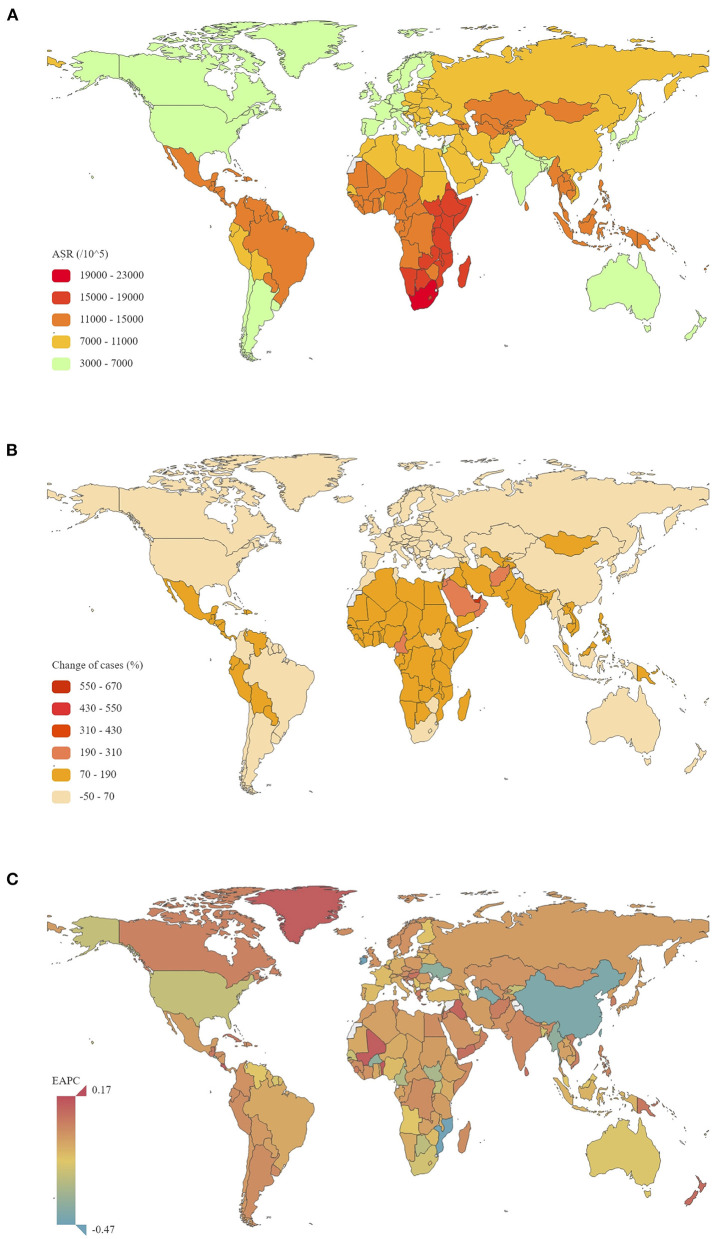
The global disease burden of STIs excluding HIV for men and women in 204 countries and territories. **(A)** The ASR of STIs excluding HIV in 2019; **(B)** The relative change in incident cases of STIs excluding HIV between 1990 and 2019; **(C)** The EAPC in STI ASR from 1990 to 2019 ASR, age-standardized rate; EAPC, estimated annual percentage change; STIs, sexually transmitted infections; HIV, human immunodeficiency virus.

**Figure 2:**
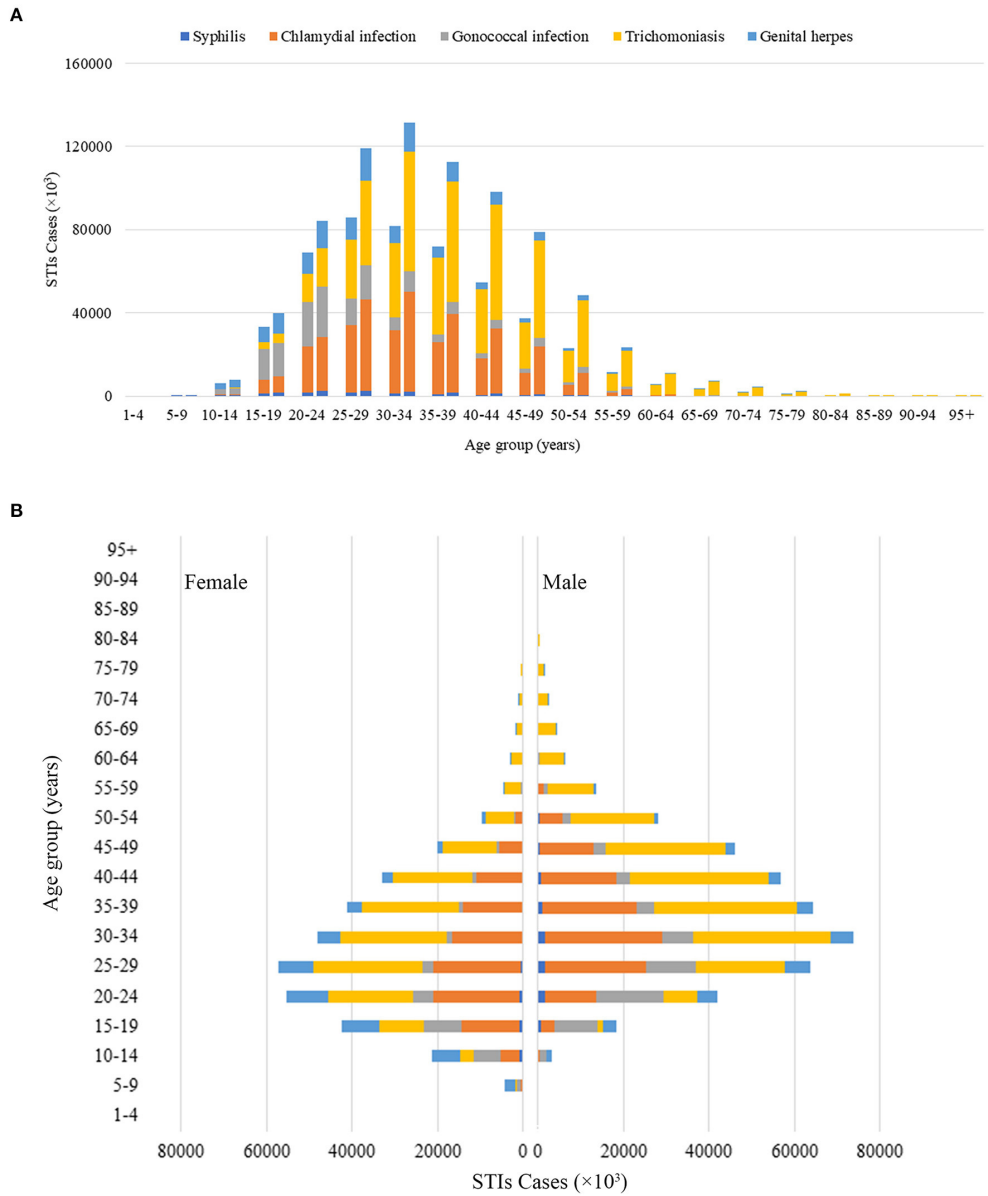
Global STIs incident cases by 20 GBD age groups. **(A)** Global STIs incident cases by age for men and women combined in 1990 and 2019. For each group, the left column presents case data from 1990 and the right column presents data from 2019. **(B)** Difference by gender in global STI incident cases by age in 2019 GBD, Global Burden of Disease; STIs, sexually transmitted infections.

Globally, from 1990 to 2019, the total number of combined incident cases of syphilis, chlamydia, gonorrhea, trichomoniasis, and genital herpes increased by 58.15% from 486.77 (95% CI 416.76 to 565.52) million to 769.85 (659.06 to 892.66) million (country-specific details see Supplementary Table 7 and Figures 1A,B). The group aged 30–34 years had the highest number of incident cases in 2019. Except for the groups aged 10–14, 15–19, 20–24, and >85 years, men had a higher incidence of all STIs than women in 2019 (426.07 vs. 343.78 million, Table 1; Figures 2A,B). The change in STI incidence varied considerably between nations, with the most prominent increase observed in Qatar (661.68%), United Arab Emirates (552.08%), and Maldives (297.78%), and the most prominent decline observed in Georgia (−36.64%). The largest number of incident cases occurred in China (172.83 million), India (99.91 million), and Indonesia (32.61 million) in 2019.

### ASR and EAPC of STIs

Globally, ASR of all STIs was 9,535.71 per 100.000 in 2019, which was significantly heterogeneous across the world, with the highest ASR in South Africa (21,759.56 per 100.000) and lowest in Belgium (3,465.76 per 100.000) (Figure 1A). From 1990 to 2019, ASR of all STIs globally decreased, with an EAPC of −0.04 (95% CI −0.09 to 0.01). However, this trend was not statistically significant. EAPC for men and women was −0.04 (95% CI −0.09 to 0.02) and −0.04 (95% CI −0.10 to 0.02), respectively (Table 1). The largest increase in ASR was seen in Mexico (0.17, 95% CI 0.12 to 0.22), while the most significant decreases were seen in Iraq (−0.47, 95% CI −0.56 to −0.38), Morocco (−0.46, 95% CI −0.52 to −0.41), and China (−0.42, 95% CI −0.59 to −0.25) from 1990 to 2019 (Table 1; Supplementary Table 7; Figure 1C).

In the analysis by geographic region, ASR was highest in Southern Sub-Saharan Africa (19,973.12 per 100.000 in 2019), Western Sub-Saharan Africa (17,033.30 per 100.000 in 2019), and Oceania (14,285.59 per 100.000 in 2019). Significant decreases in ASR from 1990 to 2019 were found in East Asia (EAPC = −0.41; 95% CI −0.57 to −0.24), Southern Sub-Saharan Africa (EAPC = −0.26; 95% CI −0.32 to −0.20), and High-income North America (EAPC = −0.24; 95% CI −0.28 to −0.20). In the analysis by SDI region, an increase in ASR from 1990 to 2019 was only observed in high SDI regions (EAPC = 0.06, 95% CI 0.05 to 0.08).

### Incidence of STIs by Pathogenic Organism

**Figure 3:**
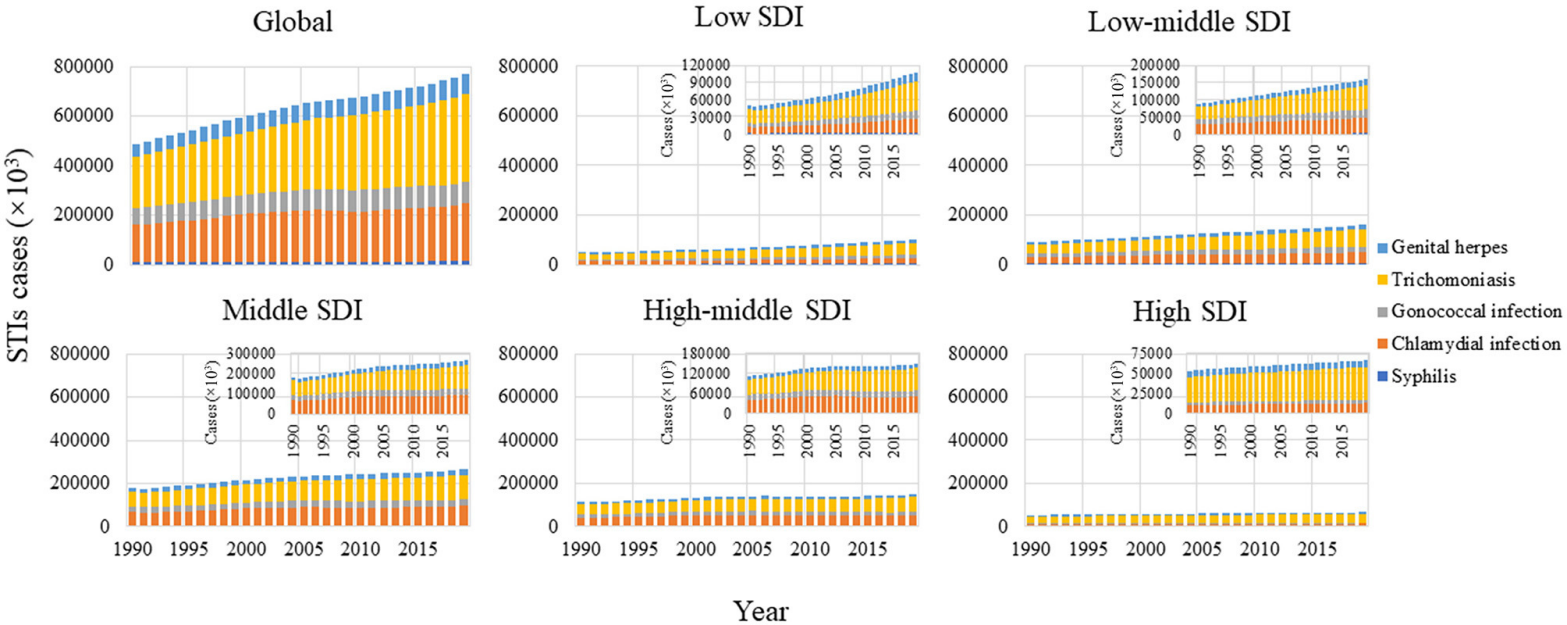
Number of incident cases of STIs excluding HIV 1990-2019, stratified by SDI region. SDI regions with lower number of cases are magnified in the top- right of the panel SDI, sociodemographic index; STIs, sexually transmitted infections; HIV, human immunodeficiency virus.

**Figure 4:**
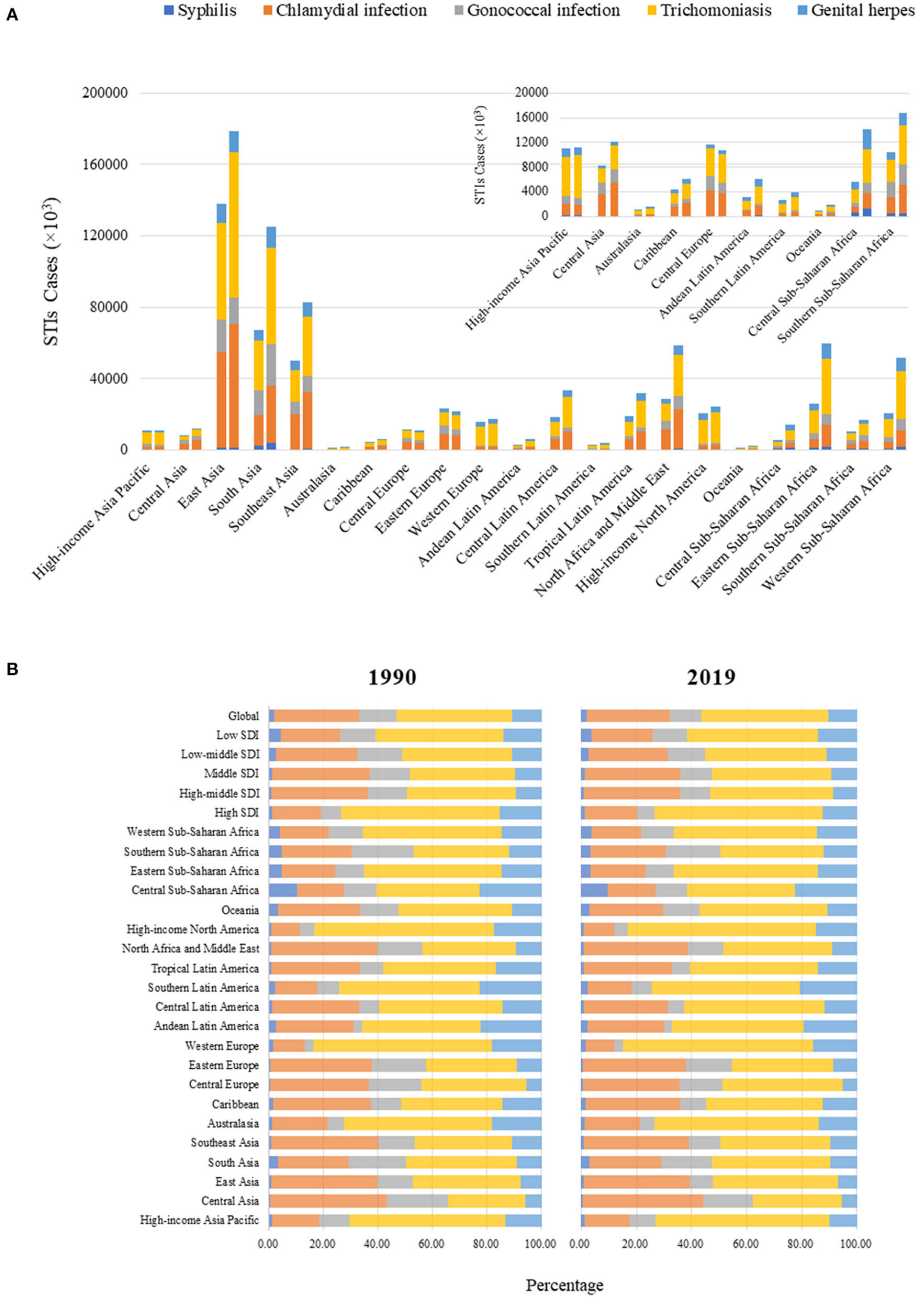
**(A)** Number of incident cases of STIs excluding HIV at the regional level. The left column in each group presents cases in 1990 and the right column presents cases in 2019. Regions with lower numbers of cases are magnified in the top right of the panel. **(B)** Proportion of syphilis, chlamydia, gonorrhea, trichomoniasis, and genital herpes in all incident cases of STIs 1990-2019, at the global, SDI region, and regional levels STIs, sexually transmitted infections; HIV, human immunodeficiency virus.

Incident cases of each STI in 1990 and 2019 at the global and regional levels are presented in Figures 3, 4. Globally in 2019, trichomoniasis incident cases outnumbered other STIs, followed by chlamydia, gonorrhea, genital herpes, and syphilis, accounting for 46.04, 30.21, 11.42, 10.49, and 1.83% of total incident cases, respectively. These proportions were relatively stable at the global and regional levels over time. Among these STIs, the largest increase in the number of incident cases was trichomoniasis (72.53%) from 1990 to 2019. Globally, ASR of syphilis increased from 1990 to 2019, with an EAPC of 0.16 (95% CI 0.06 to 0.26) (Table 1). However, ASR decreased for chlamydia and gonorrhea, with an EAPC of −0.21 (95% CI −0.36 to −0.06) and −0.14 (−0.19 to −0.08), respectively. ASR remained stable for trichomoniasis and genital herpes, with an EAPC of 0.06 (95% CI 0.03 to 0.09) and 0.09 (95% CI 0.05 to 0.13).

**Figure 5:**
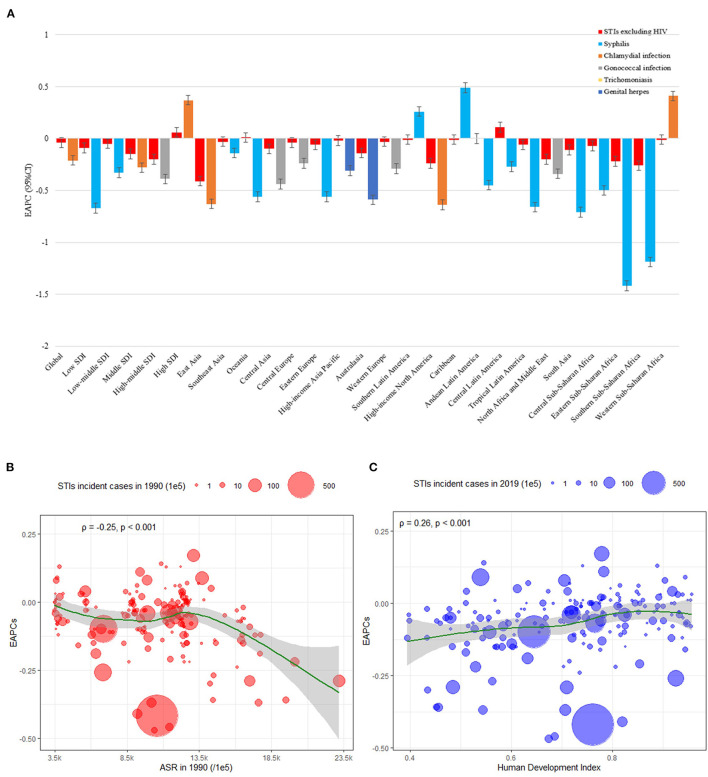
EAPCs in STI ASR excluding HIV from 1990 to 2019, at global, regional, and national levels. **(A)** EAPC in STI ASR from 1990 to 2019, by pathogenic microorganism and by region. EAPC in each region are presented as an overall and an absolute maximum caused by pathogenic microorganism. The correlation between EAPC and **(B)** STI ASR in 1990 and **(C)** HDI in 2019. The circles represent countries with available HDI data. Circle size represents total number of STI cases. The ρ indices and p values presented in **(B)** and **(C)** were derived from Pearson correlation analysis ASR, age-standardized rate; EAPC, estimated annual percentage change; HDI, human development index; STIs, sexually transmitted infections; HIV, human immunodeficiency virus.

ASR trends for each STI were significantly heterogeneous across 5 SDI regions. The highest EAPC for syphilis was observed in high-SDI regions and the Caribbean, for chlamydia in high-SDI regions and western Sub-Saharan Africa, for gonorrhea in low-middle SDI regions and Oceania, for trichomoniasis in high-SDI regions and Oceania, and for genital herpes and in low-middle SDI regions, southern Sub-Saharan Africa, and South Asia (Figure 5A). Men had a larger increase in EAPC than women for syphilis (0.43 vs. −0.30) and gonorrhea (0.07 vs. −0.52). Detailed information of the incident cases, ASR, and EAPC for each STI by age, gender, region, and country or territory are presented in supplementary files.

### Factors Associated With STIs Incidence

Figures 5B,C show the correlation between EAPC and ASR in 1990 and HDI in 2019 for all five STIs and for each of the 204 included countries and territories. A significant negative correlation (ρ=-0.25, *P* <0.001) was observed between EAPC and ASR (in 1990) for any incident STI. A significant positive relation was also detected between EAPCs and HDIs (ρ=0.26, *P* <0.001). Countries and territories with higher HDI experienced a more rapid increase in ASR from 1990 to 2019. These correlations for each of the five included STIs are presented in supplementary files.

## Discussion

To our knowledge, this is the first study to comprehensively analyze the GBD database for trends in incidence of chlamydia, gonorrhea, trichomoniasis, syphilis, and genital herpes at global, regional, and national levels from 1990 to 2019. The incident cases of these five common STIs increased by 58.15%, and ASR decreased by an average of 0.04% per year.

The trend in STI incidence over time was similar to that of other diseases whose ASR has not substantially changed (24). Global population growth appears to be the main reason for the increase in the number of incident STI cases, which increased by 25.7%, from 6.2 billion (6.0–6.3) in 2000 to 7.7 billion (7.5–8.0) in 2019, estimated by GBD 2019 Demographics Collaborators (24–26). However, trends in the incidence of these five STIs varied considerably by region and country. For example, in low and low-middle income countries, the trend in ASR for all STIs was mainly attributable to changes in syphilis. Conversely, in high-middle income regions, the decreasing trend in ASR of all STIs may be due to the decrease in the incidence of chlamydia and gonorrhea. Notably, ASR of syphilis and chlamydia was only found to be rapidly increasing in high SDI regions, a trend also reflected in the significant positive association between EAPC and HDI between 1990 and 2019. EAPC was negatively associated with baseline ASR. The baseline ASR in high-income countries was generally low, and lower the baseline ASR may explain the positive EAPCs observed in high SDI countries. It is critical to understand the exact trends in STIs with specific pathogenic microorganisms to effectively prevent STIs.

Significant increases in ASR of syphilis from 1990 to 2019 were found in male and high-SDI regions. The re-emergence of syphilis has also been reported in upper-middle-income and high-income regions, such as the United States (27, 28), Greece (29, 30), Japan (31, 32), and the UK recently (33), which is consistent with this study. Over the past decade, the incidence of syphilis among MSM has increased markedly in many countries. For example, the rate of primary or secondary syphilis among MSM in the United States increased from 11.7 cases per 100,000 population in 2014 to 18.7 per 100,000 in 2018 (34). A recent meta-analysis reported that the global pooled prevalence among MSM from 2000–2020 was 7.5% (35). Among MSM who are receiving preexposure prophylaxis against HIV infection, the incidence of syphilis is particularly high due to the increase in condomless sex (36). Although the burden of syphilis in low-SDI regions is still heavy, the high-SDI regions are areas of concern. Regular syphilis screening in the high-risk population, health education, and management of sexual partners are necessary to prevent the spread of syphilis.

Consistent with previous global estimates, we found the ASR of chlamydia and gonorrhea were highest in the middle, high middle-SDI countries and low middle, middle-SDI countries, respectively (17). Globally, the trend in ASR of chlamydia and gonorrhea decreased from 1990 to 2019. However, an increasing trend in ASR of chlamydia was observed only in countries with high SDI. Given that there are currently few data available to monitor population-based chlamydia incidence over time, the trends found in this study should be interpreted with caution. The decreasing trend of chlamydia and gonorrhea in specific populations has been reported in studies based on the Spectrum-STI model or population surveillance in some regions, such as Morocco, Washington State, Mongolia, and Western Australia (37–40). The declines in gonorrhea and chlamydia might be attributable to a combination of factors associated with the expanded HIV/STI response, including improved treatment coverage, improved reporting of cases treated, and a fall in sexual risk behaviors, possibly in part due to testing and counseling services for HIV (37). In GBD2019, the data used to estimate the incidence of chlamydia and gonorrhea are mainly derived from community surveillance data and cross-sectional research data based on the laboratory-confirmed diagnosis. Chlamydia and gonorrhea and are divided into asymptomatic and symptomatic health states, based on assumptions about the probability and duration of symptoms, including an estimate of the proportion of experiencing epididymal-orchitis (19). Chlamydia is a largely asymptomatic infection, and reported incidence is highly dependent upon rates of test uptake, particularly among asymptomatic persons at risk of infection. In high SDI countries, governments are paying more attention to screening for asymptomatic infection among sexually active young adults, which may affect estimates of the incidence of chlamydia, and partly explain the increase in incidence in these countries.

ASR of trichomoniasis and genital herpes remained stable across SDI regions from 1990 to 2019, with the highest rates being reported in lower SDI regions. Africa and Asia remain key areas for trichomoniasis and genital herpes prevention and control, which are similar to previous global surveys (17, 41). The WHO global and regional estimates for 2012 and 2016 suggested that trichomoniasis was especially common in low-income areas (17). Unlike the other four STIs, included in this study, women are more susceptible to genital herpes than men. Since many women of childbearing age have been or will be infected with HSV, the risk of transmission from mother to fetus or newborn is a major health problem (42, 43). Neonatal herpes infection is not a reportable disease, which may be why there is a high incidence of genital herpes among people aged 10–14 years in this study. Current epidemiological evidence suggests genital herpes also increases susceptibility to HIV infection and may increase HIV infectiousness in people living with HIV (44–46). However, due to the lack of data or case reports on these two STIs to investigate trends in incidence over time at national and global levels, making it is difficult to compare our findings to previously published studies.

Our study has several limitations. First, this is a secondary analysis of data extracted from the GBD 2019. The accuracy and robustness of GBD estimates depend on the quality and quantity of data used in its creation. Comparable studies are limited, so more population-based studies are needed to externally validate the findings of this study. Different sources of data in the input DisMod models, which have been clarified in the method, may hinder the comparison of the given STIs in this study. In order to sex-split data sources reported for both sexes combined, sources reporting for each sex separately were matched by age and location for each STI. Log ratios between the prevalence of each STI in females and the prevalence of each STI in males were input into MR-BRT to estimate an adjustment factor. An adjustment factor to split both sex data points into sex-specific data points was calculated for each STI, as pooled values across all ages and geographies. Second, since limited incidence data were available, estimates for a given infection and region are therefore extrapolated from a small number of data points, and ratios were used to generate estimates for some regions. The incidence in areas where key populations contribute disproportionately to sexually transmitted infection epidemics may have been underestimated despite the applied correction factor. Third, types of available tests have changed over time or are different across countries and regions. Nucleic acid amplification tests are more sensitive and specific than older techniques and are more commonly used in high SDI countries. In the absence of data on which tests were used, it is difficult to determine if changes over time or differences between locations are true or reflect differences in tests used. Fourth, many other STIs excluding HIV, are not included in the current study. Fifth, the age patterns of the incidence in this study should be interpreted with caution due to the different age patterns and different estimation processes in the input DisMod models for the incidence of different STIs. For example, GBD 2019 estimated the incidence of gonorrhea and chlamydia in a custom process outside of DisMod. Finally, the effect of the different health systems across different countries or regions was not evaluated.

The global estimates of the incidence trends of STIs are important in the first strategic direction of the WHO Global Health Sector Strategy. Currently, the Spectrum-STI estimation tool is often used to estimate the trend of national STI incidence (17, 47, 48). Compared with the GBD study, systematic reviews in the Spectrum-STI are updated, and its age group and gender data are more accurate (17). However, due to the large differences in the quality of the data included between different countries, it may affect the comprehensive comparison of global trends. The systematic literature reviews for incidence input data on STIs in GBD 2019 were completed on April 17, 2015 (19). Although the data processing and modeling strategy were different, it still needs to be updated. Despite the use of correction factors in a representative sample of the general population, such as age groups and sex ratios, the quality of studies on the incidence of STIs needs to be improved. The comparison between GBD2017 and GBD2019 in this study shows that the difference in EAPC is essentially <0.1, and the reason for the difference can be found on the official website of GBD 2019 (http://ghdx.healthdata.org/gbd-2019). The process of generating future incidence estimates can be made more effective through continuously updated systematic reviews and continuously optimized model strategies. More population-based national monitoring data are needed to verify the accuracy of GBD estimates in STIs.

STIs remain a major public health concern globally. Efforts to combat STIs in lower-income countries are commendable. Estimates of incidence trends are essential for effective control of STIs, optimization of primary and secondary prevention strategies, including enhanced screening programs in high-risk regions, active health promotion, and construction of comprehensive STI surveillance networks. Despite the weaknesses, this study will fill a gap where actual data on STIs burden are sparse or unavailable.

## Data Availability Statement

All data are available from the Global Health Data Exchange query tool (http://ghdx.healthdata.org/gbd-results-tool).

## Author Contributions

HZ conceived the study and designed the protocol with LF. LF, YS, MH, and YZ performed analyses of the Global Burden of Disease data. LF, YS, MH, and BW contributed to statistical analysis and interpretation of data. LF, TY, PL, YG, and CF drafted the manuscript with all authors critically revising the manuscript. All authors contributed to the article and approved the submitted version.

## Funding

This study was supported by the Natural Science Foundation of China Excellent Young Scientists Fund (82022064), Natural Science Foundation of China International/Regional Research Collaboration Project (72061137001), Natural Science Foundation of China Young Scientist Fund (81703278), the Australian National Health and Medical Research Commission (NHMRC) Early Career Fellowship (APP1092621), the National Science and Technology Major Project of China (2018ZX10721102), the Sanming Project of Medicine in Shenzhen (SZSM201811071), the High Level Project of Medicine in Longhua, Shenzhen (HLPM201907020105), the National Key Research and Development Program of China (2020YFC0840900), and the Fundamental Research Funds for the Central Universities (51000-42180001). All funding parties did not have any role in the design of the study or in the explanation of the data.

## Conflict of Interest

The authors declare that the research was conducted in the absence of any commercial or financial relationships that could be construed as a potential conflict of interest.

## Publisher's Note

All claims expressed in this article are solely those of the authors and do not necessarily represent those of their affiliated organizations, or those of the publisher, the editors and the reviewers. Any product that may be evaluated in this article, or claim that may be made by its manufacturer, is not guaranteed or endorsed by the publisher.
