# Supplementary material for: Incidence Trends of Five Common Sexually Transmitted Infections Excluding HIV From 1990 to 2019 at the Global, Regional, and National Levels: Results From the Global Burden of Disease Study 2019
Source: Front Med (Lausanne). 2022 Mar 2;9:851635. doi: 10.3389/fmed.2022.851635 (PMC8924524; doi:10.3389/fmed.2022.851635)

**SUPPLEMENTARY MATERIAL**

**Syphilis**

Globally, syphilis accounted for 1.83% of total STIs incident cases in 2019 (Figure 4). The overall ASR of syphilis was 178 per 100 0000 in 2019, with the EAPC of 0.16 (95% CI 0.06 to 0.26). The absolute number of incident cases of syphilis increased by 59.57% from 8845220 in 1990 to 14114110 in 2019 (Table S1). The most prominent incident cases happened in India, China and Nigeria, and the corresponding change percentage were 52.28%, 19.29% and 128.56%, with country-specific details in Table S6 and Figure S2. For SDI regions, the ASR were increased in high- and high-middle-SDI and decreased in the other three lower SDI regions. For geographical regions, the highest increase of the syphilis cases observed in Western Sub-Saharan Africa (133.05 %), followed by Central Sub-Saharan Africa (129.38%) (Figure 4). The top 2 EAPCs observed in Caribbean (0.49, 95% CI 0.43 to 0.55) and High-income Asia Pacific (0.28, 95% CI 0.22 to 0.34) (Table S1). The EAPC of syphilis in males was much higher than that in females. (Table S1)

**Chlamydia**

In 2019, chlamydia increased nearly 30.21% of the total number of STIs (Figure 4). The global incident cases of chlamydia increased by 53.29% from 1990 to 2019 (Table S2). The highest incident cases were in China, followed by India, Indonesia and Brazil, and the corresponding change percentage were 29.94%, 84.46%, 60.47% and 64.14%. The overall ASR was 2884 per 100 000 in 2019, with the highest ASR in Southern Sub-Saharan Africa (5324 per 100 000) and the corresponding EAPC was 0.09 (95%CI -0.07 to 0.26) in this period (Table S2, 6). For SDI regions, the most pronounced increase was noted high-SDI regions (EAPC = 0.37, 95% CI 0.29 to 0.46). For geographical regions, the most pronounced increase was observed in Western Sub-Saharan Africa (EAPC = 0.41, 95% CI 0.28 to 0.54) and significantly decreased in High-income North America (EAPC = −0.64, 95% CI −0.99 to −0.30). (Table S2).

**Gonorrhoea**

In 2019, about 11.42% of total STIs incident cases was ascribed to gonorrhoea (Figure 4). The incident cases of gonorrhoea increased by 29.85% from 1990 to 2019 (Table S3). The most prominent incident cases were observed in India, China and Indonesia, and the corresponding change percentage were 62.64%, −18.32% and 38.55%, with country-specific details in Table S6. The global ASR was 1124 per 100 000 in 2019, with the highest ASR in Southern Sub-Saharan Africa (3870 per 100 000 in 2019). The overall ASR of gonorrhoea decreased in this period, with the EAPC of −0.14 (95% CI −0.19 to −0.08). For SDI regions, the ASR decreased in all five SDI regions. For geographical regions, the most obvious increase and decrease were noted in Oceania and East Asia, with the EAPC of 0.37 (95% CI 0.26 to 0.48) and −0.54 (95% CI −0.59 to −0.49), respectively. (Table S3). The EAPC of gonorrhoea in males was much higher than that in females. (Table S3)

**Trichomoniasis**

In 2019, STIs due to trichomoniasis accounted for 46.04% of total cases (Figure 4). Globally, the ASR of trichomoniasis remained stable from 1990 to 2019 (EAPC = 0.06, 95% CI 0.03 to 0.09), though the absolute number of incident cases increased by 72.53% in this period (Table S4). With respect to countries, the maximum number of cases were noted in China, India and USA, and the corresponding change percentage were 49.02%, 93.28% and 22.70%, with country-specific details in Table S6. For SDI regions, trichomoniasis incident cases increased across all 5 regions, while the ASR relatively stable in all 5 regions (Table S4). For geographical regions, the most obvious increase and decrease trend of ASR was observed in Oceania (EAPC = 0.24, 95% CI 0.17 to 0.32) and Southern Sub-Saharan Africa (EAPC = −0.49, 95% CI −0.63 to −0.35). (Table S4). The relatively higher ASR were observed in United Republic of Tanzania, Zambia and Ethiopia. The EAPC of trichomoniasis in females was no different from that in males. (Table S4)

**Genital herpes**

Globally, genital herpes accounted for 10.49% of total STIs incident cases in 2019 (Figure 4). The overall ASR of genital herpes was 1022 per 100 0000 in 2019, with the EAPC of 0.09 (95% CI 0.05 to 0.13). The absolute number of incident cases of genital herpes increased by 52.28% from 53051595 in 1990 to 80784427 in 2019 (Table S5). The most prominent incident cases were in China, India and Brazil, and the corresponding change percentage were 17.14%, 100.86% and 41.8%, with country-specific details in Table S6 and Figure S14. For SDI regions, the ASR were increased in low-middle- and middle-SDI regions, decreased in the other three SDI regions. For geographical regions, the highest increase of the genital herpes cases observed in Western Sub-Saharan Africa (151.97%), followed by Central Sub-Saharan Africa (148.79%) (Figure 3). The most obvious increase trend of ASR was noted in South Asia (EAPC = 0.25, 95% CI 0.19 to 0.32) and Southern Sub-Saharan Africa (EAPC = 0.25, 95% CI 0.15 to 0.35). The most pronounced decrease was observed in Eastern Sub-Saharan Africa and Australasia, with the EAPC of −0.60 (95% CI −0.77 to −0.43) and −0.59 (95% CI −1.03 to −0.15), respectively. (Table S5)

**Table S1. Incident cases and age-standardized incidence rates of syphilis in 1990 and 2019 and estimated annual percentage change from 1990 to 2019.**

| **Characteristics** | **1990** | | **2019** | | **1990-2019** |
| --- | --- | --- | --- | --- | --- |
|  | **Incident cases**  **No. ×10^3^ (95% UI)** | **ASR per 100,000**  **No. (95% UI)** | **Incident cases**  **No. ×10^3^ (95% UI)** | **ASR per 100,000**  **No. (95% UI)** | **EAPC**  **No. (95% CI)** |
| **Overall** | 8845.22 (6562.51-11588.86) | 160.03 (120.66-208.10) | 14114.11 (10648.49-18415.97) | 178.48 (134.94-232.34) | 0.16 (0.06 to 0.26) |
| **Sex** |  |  |  |  |  |
| **Male** | 5489.77 (4066.36-7208.42) | 199.14 (149.81-260.75) | 9289.25 (6919.20-12282.79) | 231.31 (171.88-305.30) | 0.43 (0.38 to 0.47) |
| **Female** | 3355.45 (2502.21-4388.06) | 119.95 (90.17-156.34) | 4824.86 (3725.83-6182.48) | 124.98 (96.68-160.25) | -0.30 (-0.50 to -0.10) |
| **SDI category** |  |  |  |  |  |
| **Low-SDI** | 2267.02 (1720.58-2889.82) | 467.40 (356.95-593.73) | 4116.13 (3146.92-5287.15) | 372.53 (287.4-475.57) | -0.67 (-0.75 to -0.58) |
| **Low-middle-SDI** | 2459.57 (1804.25-3253.48) | 224.35 (166.26-294.69) | 4042.00 (3000.00-5301.94) | 214.66 (160.77-280.80) | -0.33 (-0.46 to -0.20) |
| **Middle-SDI** | 2477.83 (1809.89-3280.76) | 136.26 (102.10-179.16) | 3310.60 (2471.37-4388.12) | 130.13 (96.65-171.91) | -0.14 (-0.28 to 0.00) |
| **High-middle-SDI** | 1011.91 (754.28-1333.58) | 82.22 (61.68-108.01) | 1348.09 (1026.99-1774.22) | 90.96 (68.81-118.20) | 0.09 (-0.01 to 0.20) |
| **High-SDI** | 623.30 (471.90-826.55) | 71.52 (54.07-94.72) | 721.92 (548.59-951.88) | 74.05 (55.61-98.92) | 0.16 (0.10 to 0.22) |
| **Region** |  |  |  |  |  |
| **East Asia** | 1224.26 (895.45-1631.22) | 89.28 (66.65-118.27) | 1459.54 (1092.02-1958.96) | 93.43 (69.27-123.92) | -0.08 (-0.22 to 0.05) |
| **Southeast Asia** | 487.64 (352.37-649.91) | 101.17 (74.45-134.79) | 722.67 (535.24-961.37) | 99.61 (73.45-131.74) | -0.14 (-0.19 to -0.09) |
| **Oceania** | 28.67 (20.98-37.65) | 430.43 (320.80-560.81) | 57.91 (42.04-76.97) | 415.80 (301.62-551.31) | -0.56 (-0.74 to -0.37) |
| **Central Asia** | 37.39 (28.05-49.21) | 54.85 (41.88-71.72) | 51.63 (39.50-66.88) | 52.10 (40.26-67.34) | -0.36 (-0.42 to -0.30) |
| **Central Europe** | 54.15 (41.35-71.11) | 43.38 (32.95-56.96) | 47.32 (36.32-61.63) | 43.28 (32.96-56.54) | -0.02 (-0.05 to 0.01) |
| **Eastern Europe** | 125.74 (96.17-165.15) | 53.10 (40.90-68.98) | 102.35 (78.85-136.35) | 47.97 (37.05-62.56) | -0.56 (-0.65 to -0.47) |
| **High-income Asia Pacific** | 145.87 (110.95-192.31) | 78.31 (59.18-103.74) | 136.11 (103.15-181.01) | 81.36 (60.92-109.14) | 0.28 (0.22 to 0.34) |
| **Australasia** | 14.10 (10.46-18.96) | 65.58 (48.71-87.54) | 17.71 (13.32-23.46) | 64.07 (47.75-85.84) | -0.12 (-0.13 to -0.10) |
| **Western Europe** | 269.53 (201.47-355.93) | 68.34 (51.17-90.38) | 263.00 (199.22-348.20) | 67.63 (50.12-90.19) | -0.03 (-0.04 to -0.02) |
| **Southern Latin America** | 58.65 (43.84-77.44) | 117.88 (155.84-88.25) | 88.79 (71.68-110.38) | 131.89 (106.80-163.69) | 0.26 (0.07 to 0.45) |
| **High-income North America** | 205.18 (154.80-273.09) | 68.27 (89.69-51.74) | 252.31 (192.72-331.60) | 71.76 (54.06-95.06) | 0.18 (-0.04 to 0.40) |
| **Caribbean** | 61.44 (45.70-81.46) | 165.87 (216.09-125.65) | 92.37 (75.16-113.65) | 191.99 (156.21-235.61) | 0.49 (0.43 to 0.55) |
| **Andean Latin America** | 83.04 (61.30-110.51) | 221.73 (293.67-167.05) | 138.14 (104.70-180.53) | 207.84 (158.51-270.68) | -0.45 (-0.55 to -0.35) |
| **Central Latin America** | 195.78 (143.30-259.92) | 118.92 (156.63-89.05) | 296.13 (227.21-384.26) | 111.77 (86.08-145.02) | -0.27 (-0.33 to -0.21) |
| **Tropical Latin America** | 190.64 (139.63-255.94) | 119.04 (158.58-88.38) | 332.79 (259.02-415.93) | 139.68 (109.18-174.26) | -0.66 (-1.53 to 0.21) |
| **North Africa and Middle East** | 262.02 (189.03-348.87) | 79.67 (106.06-59.21) | 562.03 (415.47-750.43) | 84.20 (62.65-111.70) | 0.15 (0.06 to 0.24) |
| **South Asia** | 2332.44 (1691.29-3098.81) | 219.04 (289.42-161.13) | 3725.19 (2730.12-4965.90) | 190.99 (140.95-254.13) | -0.71 (-0.96 to -0.46) |
| **Central Sub-Saharan Africa** | 580.89 (435.01-749.57) | 1153.28 (1482.29-880.53) | 1332.45 (1017.53-1723.69) | 1048.40 (803.52-1344.82) | -0.50 (-0.59 to -0.40) |
| **Eastern Sub-Saharan Africa** | 1181.60 (916.25-1480.69) | 669.08 (834.07-522.83) | 1992.07 (1551.25-2495.06) | 492.63 (386.33-619.27) | -1.42 (-1.62 to -1.22) |
| **Southern Sub-Saharan Africa** | 502.74 (381.66-648.48) | 916.80 (1174.15-697.82) | 571.16 (420.69-751.87) | 665.35 (496.08-872.21) | -1.19 (-1.71 to -0.67) |
| **Western Sub-Saharan Africa** | 803.45 (591.41-1043.07) | 461.93 (598.09-345.94) | 1872.43 (1377.02-2458.53) | 427.97 (318.21-556.32) | -0.26 (-0.31 to -0.20) |

**UI, uncertainty interval; ASR, age-standardized incidence rate; CI, confidential interval; EAPC, estimated annual percentage change.**

**Table S2. Incident cases and age-standardized incidence rates of chlamydia in 1990 and 2019 and estimated annual percentage change from 1990 to 2019.**

| **Characteristics** | **1990** | | **2019** | | **1990-2019** |
| --- | --- | --- | --- | --- | --- |
|  | **Incident cases**  **No. ×10^3^ (95% UI)** | **ASR per 100,000**  **No. (95% UI)** | **Incident cases**  **No. ×10^3^ (95% UI)** | **ASR per 100,000**  **No. (95% UI)** | **EAPC**  **No. (95% CI)** |
| **Overall** | 151695.68 (113998.56-199144.01) | 2867.67 (2150.60-3741.43) | 232534.84 (174269.19-303009.11) | 2883.87 (2161.21-3762.80) | -0.21 (-0.36 to -0.06) |
| **Sex** |  |  |  |  |  |
| **Male** | 83979.50 (62121.37-111433.61) | 3147.30 (2334.47-4129.53) | 125599.24 (93186.99-164518.16) | 3088.09 (2286.90-4039.30) | -0.29 (-0.45 to -0.14) |
| **Female** | 67716.18 (51485.41-88169.74) | 2579.57 (1964.61-3360.43) | 106935.60 (81029.30-139409.31) | 2677.33 (2027.51-3505.38) | -0.10 (-0.25 to 0.05) |
| **SDI category** |  |  |  |  |  |
| **Low-SDI** | 10987.51 (8267.17-14395.59) | 2501.78 (1887.37-3273.67) | 23881.03 (17864.30-31402.29) | 2359.48 (1770.16-3098.25) | -0.04 (-0.12 to 0.04) |
| **Low-middle-SDI** | 26592.63 (19958.90-34845.68) | 2613.36 (1957.08-3396.20) | 46203.05 (34745.36-60364.04) | 2524.34 (1894.78-3281.85) | -0.26 (-0.33 to -0.19) |
| **Middle-SDI** | 64740.67 (48605.29-85278.85) | 3795.24 (2844.39-4961.53) | 91514.83 (68525.50-118889.12) | 3477.61 (2601.94-4553.28) | -0.28 (-0.45 to -0.11) |
| **High-middle-SDI** | 39816.12 (29638.64-52282.05) | 3252.41 (2425.07-4244.58) | 52103.68 (38909.69-68070.89) | 3262.50 (2430.97-4273.94) | -0.36 (-0.59 to -0.14) |
| **High-SDI** | 9453.54 (7136.58-12326.73) | 1066.96 (809.15-1396.85) | 12557.26 (9503.56-16097.73) | 1241.28 (936.52-1617.46) | 0.37 (0.29 to 0.46) |
| **Region** |  |  |  |  |  |
| **East Asia** | 53473.62 (39843.02-70577.94) | 4049.64 (3024.98-5270.00) | 69208.69 (51730.09-90227.82) | 4128.96 (3082.92-5375.67) | -0.63 (-1.05 to -0.22) |
| **Southeast Asia** | 19519.57 (14690.93-25768.43) | 4330.29 (3251.49-5622.52) | 31419.20 (23507.05-40988.40) | 4252.09 (3180.39-5558.88) | 0.00 (-0.02 to 0.03) |
| **Oceania** | 263.63 (202.41-332.98) | 4212.36 (3273.43-5296.76) | 497.73 (376.55-646.71) | 3701.31 (2826.22-4759.19) | -0.52 (-0.59 to -0.44) |
| **Central Asia** | 3523.02 (2629.40-4678.71) | 5277.11 (3947.90-6912.30) | 5347.63 (4011.01-7000.18) | 5271.28 (3946.14-6857.60) | -0.03 (-0.05 to -0.02) |
| **Central Europe** | 4199.57 (3126.67-5503.93) | 3343.90 (2499.19-4405.11) | 3749.00 (2796.76-4867.00) | 3308.59 (2464.68-4333.19) | -0.04 (-0.05 to -0.04) |
| **Eastern Europe** | 8720.28 (6515.05-11356.62) | 3644.40 (2722.06-4737.11) | 8021.15 (5945.68-10566.80) | 3625.05 (2701.7-4753.04) | -0.01 (-0.02 to 0.00) |
| **High-income Asia Pacific** | 1900.40 (1430.40-2459.84) | 1005.98 (753.84-1306.50) | 1818.45 (1377.70-2352.24) | 1025.36 (767.38-1355.75) | 0.09 (0.05 to 0.13) |
| **Australasia** | 233.48 (174.59-303.88) | 1068.87 (804.24-1386.40) | 304.57 (228.75-392.63) | 1051.10 (784.76-1364.78) | -0.09 (-0.24 to 0.07) |
| **Western Europe** | 1765.79 (1345.72-2296.88) | 435.95 (331.57-568.59) | 1821.28 (1387.74-2326.50) | 429.93 (327.62-559.92) | -0.04 (-0.09 to 0.01) |
| **Southern Latin America** | 429.76 (326.76-554.61) | 885.29 (671.59-1144) | 638.05 (481.36-826.91) | 908.41 (1182.24-686) | 0.10 (0.08 to 0.13) |
| **High-income North America** | 2125.36 (1589.06-2797.12) | 695.29 (521.41-915.65) | 2640.55 (2004.91-3404.76) | 740.09 (968.78-554.75) | -0.64 (-0.99 to -0.3) |
| **Caribbean** | 1552.05 (1161.08-2032.96) | 4394.72 (3283.30-5756.82) | 2116.06 (1579.50-2760.07) | 4340.05 (5660.33-3228.3) | -0.04 (-0.04 to -0.04) |
| **Andean Latin America** | 896.76 (665.31-1171.19) | 2554.1 (1884.77-3342.26) | 1671.14 (1240.74-2196.71) | 2520.26 (3304.31-1870.63) | 0.14 (0.09 to 0.19) |
| **Central Latin America** | 5921.99 (4464.42-7722.56) | 3868.04 (2927.51-5035.54) | 10188.17 (7689.51-13366.11) | 3850 (5041.57-2903.85) | 0.19 (0.12 to 0.26) |
| **Tropical Latin America** | 6153.27 (4576.43-8178.21) | 4082.07 (3067.76-5372.83) | 10160.67 (7608.14-13245.88) | 4102.22 (5393.45-3075.72) | -0.17 (-0.30 to -0.03) |
| **North Africa and Middle East** | 11189.88 (8450.63-14569.26) | 3595.96 (2747.60-4638.74) | 22026.56 (16586.65-28782.38) | 3264.95 (4246.76-2470.04) | -0.23 (-0.27 to -0.19) |
| **South Asia** | 17312.76 (12939.60-22983.08) | 1731.73 (1290.19-2262.77) | 32645.93 (24256.65-42944.98) | 1711.93 (2239.34-1270.93) | -0.22 (-0.37 to -0.07) |
| **Central Sub-Saharan Africa** | 975.90 (724.48-1295.62) | 2134.79 (1580.51-2827.54) | 2459.76 (1808.21-3278.84) | 2111.27 (2799-1568.66) | -0.02 (-0.04 to 0.00) |
| **Eastern Sub-Saharan Africa** | 5176.09 (3899.01-6751.61) | 3328.85 (2535.14-4331.98) | 11947.26 (8919.21-15716.64) | 3223.05 (4236.64-2422.13) | -0.14 (-0.18 to -0.10) |
| **Southern Sub-Saharan Africa** | 2674.33 (2022.74-3491.08) | 5439.95 (4127.48-7073.07) | 4537.13 (3412.07-5945.06) | 5324.43 (6940.61-4039.48) | 0.09 (-0.07 to 0.26) |
| **Western Sub-Saharan Africa** | 3688.15 (2764.90-4870.38) | 2291.53 (1725.68-3009.75) | 9315.85 (6989.82-12315.14) | 2316.4 (3047.29-1741.46) | 0.41 (0.28 to 0.54) |

**UI, uncertainty interval; ASR, age-standardized incidence rate; CI, confidential interval; EAPC, estimated annual percentage change.**

**Table S3. Incident cases and age-standardized incidence rates of gonorrhoea in 1990 and 2019 and estimated annual percentage change from 1990 to 2019.**

| **Characteristics** | **1990** | | **2019** | | **1990-2019** |
| --- | --- | --- | --- | --- | --- |
|  | **Incident cases**  **No. ×10^3^ (95% UI)** | **ASR per 100,000**  **No. (95% UI)** | **Incident cases**  **No. ×10^3^ (95% CUI)** | **ASR per 100,000**  **No. (95% UI)** | **EAPC**  **No. (95% CI)** |
| **Overall** | 67732.22 (51820.12-89251.61) | 1178.58 (912.29-1536.00) | 87951.95 (68461.02-112961.84) | 1124.39 (872.97-1441.08) | -0.14 (-0.19 to -0.08) |
| **Sex** |  |  |  |  |  |
| **Male** | 43482.63 (33232.93-57318.57) | 1505.50 (1165.86-1957.26) | 59319.26 (45872.40-76110.80) | 1494.21 (1154.1-1922.58) | 0.07 (0.00 to 0.14) |
| **Female** | 24249.60 (18176.84-32574.08) | 843.96 (641.86-1118.68) | 28632.70 (22146.97-37000.15) | 746.62 (573.55-970.89) | -0.52 (-0.58 to -0.47) |
| **SDI category** |  |  |  |  |  |
| **Low-SDI** | 6493.86 (4954.93-8505.53) | 1262.07 (994.05-1617.96) | 13311.88 (10136.01-17794.05) | 1121.20 (876.87-1461.59) | -0.11 (-0.19 to -0.04) |
| **Low-middle-SDI** | 14601.88 (11111.17-19326.71) | 1238.37 (955.62-1614.98) | 22100.89 (17018.65-28800.09) | 1141.32 (883.23-1474.10) | -0.08 (-0.20 to 0.03) |
| **Middle-SDI** | 26685.20 (19832.41-36187.31) | 1367.36 (1036.82-1830.88) | 30185.57 (23252.89-39609.01) | 1225.96 (936.47-1618.37) | -0.20 (-0.25 to -0.14) |
| **High-middle-SDI** | 16032.64 (12328.77-21132.25) | 1285.36 (993.00-1683.57) | 15652.96 (12166.99-20103.29) | 1167.61 (896.78-1523.30) | -0.39 (-0.44 to -0.34) |
| **High-SDI** | 3881.18 (3109.10-4948.05) | 462.86 (369.07-587.06) | 4193.30 (3379.69-5265.41) | 458.63 (365.83-581.52) | -0.13 (-0.17 to -0.09) |
| **Region** |  |  |  |  |  |
| **East Asia** | 18170.09 (12540.69-26214.00) | 1232.18 (870.03-1743.36) | 14929.58 (10824.81-20281.77) | 1088.78 (765.14-1508.29) | -0.54 (-0.59 to -0.49) |
| **Southeast Asia** | 6751.48 (5297.40-8562.08) | 1341.76 (1073.29-1687.69) | 9590.32 (7623.98-12122.15) | 1335.19 (1061.49-1687.72) | -0.14 (-0.19 to -0.09) |
| **Oceania** | 122.08 (80.19-188.98) | 1719.15 (1184.77-2563.92) | 244.93 (161.32-377.42) | 1693.15 (1139.36-2590.01) | 0.37 (0.26 to 0.48) |
| **Central Asia** | 1835.57 (1220.28-2750.02) | 2472.40 (1688.06-3659.72) | 2192.50 (1500.13-3278.28) | 2269.24 (1542.52-3364.37) | -0.44 (-0.50 to -0.39) |
| **Central Europe** | 2257.75 (1729.32-3053.13) | 1912.48 (1442.88-2618.76) | 1671.53 (1310.71-2183.02) | 1816.54 (1375.73-2454.40) | -0.24 (-0.26 to -0.23) |
| **Eastern Europe** | 4660.52 (3431.44-6400.48) | 2155.48 (1564.29-3003.93) | 3608.59 (2639.90-4898.40) | 2115.43 (1511.49-2972.55) | -0.28 (-0.33 to -0.23) |
| **High-income Asia Pacific** | 1248.93 (995.48-1561.99) | 679.57 (539.62-856.22) | 1051.07 (855.02-1317.75) | 671.08 (530.73-845.19) | -0.06 (-0.08 to -0.03) |
| **Australasia** | 67.59 (51.53-88.83) | 322.63 (245.25-424.84) | 81.25 (62.30-106.35) | 306.23 (230.23-408.85) | -0.20 (-0.22 to -0.18) |
| **Western Europe** | 530.59 (427.60-661.07) | 136.33 (108.60-172.43) | 518.61 (422.09-638.67) | 130.45 (104.25-165.78) | -0.29 (-0.33 to -0.25) |
| **Southern Latin America** | 219.50 (155.93-311.84) | 438.40 (315.10-616.12) | 289.06 (209.85-400.24) | 430.95 (309.16-610.91) | -0.08 (-0.10 to -0.07) |
| **High-income North America** | 1064.05 (795.09-1413.07) | 372.72 (277.25-498.14) | 1266.29 (956.62-1679.17) | 375.03 (277.70-501.02) | -0.28 (-0.43 to -0.14) |
| **Caribbean** | 479.03 (322.38-733.91) | 1205.31 (832.24-1791.07) | 575.18 (399.66-855.10) | 1199.47 (829.79-1791.33) | -0.18 (-0.27 to -0.10) |
| **Andean Latin America** | 104.32 (71.40-154.59) | 259.59 (184.84-369.47) | 170.41 (120.24-244.58) | 254.51 (180.90-363.56) | -0.05 (-0.16 to 0.07) |
| **Central Latin America** | 1421.82 (1088.63-1879.11) | 788.91 (615.39-1011.34) | 2060.35 (1606.45-2650.38) | 772.79 (604.45-993.38) | -0.08(-0.10 to -0.06) |
| **Tropical Latin America** | 1577.37 (1080.32-2290.51) | 932.92 (651.29-1331.07) | 2111.25 (1470.97-2974.90) | 912.93 (631.86-1293.01) | -0.29 (-0.49 to -0.09) |
| **North Africa and Middle East** | 4782.86 (3445.48-6760.76) | 1302.37 (973.98-1769.34) | 7664.76 (5628.62-10813.33) | 1164.03 (853.33-1642.04) | -0.34 (-0.37 to -0.31) |
| **South Asia** | 14044.60 (9932.30-20014.49) | 1237.69 (885.46-1730.36) | 22793.16 (16284.78-32261.40) | 1134.27 (814.83-1598.03) | 0.05 (-0.14 to 0.25) |
| **Central Sub-Saharan Africa** | 671.99 (455.63-994.90) | 1238.79 (878.90-1760.98) | 1608.74 (1094.81-2396.95) | 1187.74 (842.19-1721.81) | -0.16 (-0.19 to -0.13) |
| **Eastern Sub-Saharan Africa** | 2711.81 (2005.51-3677.88) | 1434.45 (1116.98-1888.56) | 6055.17 (4502.20-8305.34) | 1363.62 (1054.26-1804.90) | -0.16 (-0.21 to -0.10) |
| **Southern Sub-Saharan Africa** | 2398.06 (1788.86-3210.42) | 4141.94 (3158.45-5461.20) | 3316.11 (2520.35-4404.29) | 3869.55 (2945.69-5086.97) | -0.42 (-0.57 to -0.27) |
| **Western Sub-Saharan Africa** | 2612.18 (2040.39-3385.85) | 1413.27 (1132.45-1780.93) | 6153.10 (4781.85-8077.31) | 1318.35 (1046.77-1674.62) | -0.25 (-0.27 to -0.22) |

**UI, uncertainty interval; ASR, age-standardized incidence rate; CI, confidential interval; EAPC, estimated annual percentage change.**

**Table S4. Incident cases and age-standardized incidence rates of trichomoniasis in 1990 and 2019 and estimated annual percentage change from 1990 to 2019.**

| **Characteristics** | **1990** | | **2019** | | **1990-2019** |
| --- | --- | --- | --- | --- | --- |
|  | **Incident cases**  **No. ×10^3^ (95% UI)** | **ASR per 100,000**  **No. (95% UI)** | **Incident cases**  **No. ×10^3^ (95% UI)** | **ASR per 100,000**  **No. (95% UI)** | **EAPC**  **No. (95% CI)** |
| **Overall** | 205446.49 (151261.12-273107.88) | 4157.14 (3061.97-5439.34) | 354466.58 (260117.34-461359.68) | 4327.29 (3176.53-5645.76) | 0.06 (0.03 to 0.09) |
| **Sex** |  |  |  |  |  |
| **Male** | 117931.15 (87212.23-157404.94) | 4783.30 (3546.74-6265.41) | 200627.08 (147915.66-262592.84) | 4879.67 (3610.00-6366.99) | 0.06 (0.05 to 0.07) |
| **Female** | 87515.34 (64115.94-116821.70) | 3526.67 (2549.78-4680.89) | 153839.49 (110414.26-202493.34) | 3781.34 (2719.77-5010.59) | 0.08 (0.00 to 0.16) |
| **SDI category** |  |  |  |  |  |
| **Low-SDI** | 1417.84 (1049.53-1850.02) | 6205.09 (4630.64-8055.23) | 50979.93 (37726.77-67927.47) | 5748.06 (4236.67-7502.65) | -0.05 (-0.15 to 0.05) |
| **Low-middle-SDI** | 10516.36 (7889.17-14018.11) | 3943.19 (2908.65-5161.43) | 70589.53 (51840.01-93425.62) | 4051.78 (2978.09-5277.78) | 0.04 (0.02 to 0.07) |
| **Middle-SDI** | 69999.74 (51501.40-93455.03) | 4561.33 (3350.11-5960.63) | 114940.44 (83136.09-149423.6) | 4267.97 (3116.15-5565.46) | -0.08 (-0.15 to -0.01) |
| **High-middle-SDI** | 27503.13 (20138.86-37028.79) | 3762.35 (2754.87-4936.43) | 66379.85 (48427.61-85845.12) | 3829.67 (2796.45-4987.86) | -0.03 (-0.07 to 0.02) |
| **High-SDI** | 7809.61 (5668.28-10461.71) | 3395.55 (2497.77-4457.18) | 40190.36 (29714.89-51420.48) | 3481.39 (2552.86-4554.16) | 0.07 (0.06 to 0.09) |
| **Region** |  |  |  |  |  |
| **East Asia** | 54526.57 (39873.29-72786.08) | 4475.04 (3264.13-5874.81) | 81206.63 (59123.78-105278.99) | 4403.83 (3195.76-5762.80) | -0.17 (-0.25 to -0.09) |
| **Southeast Asia** | 8362.00 (6196.65-11160.86) | 4449.71 (3303.43-5796.64) | 32948.13 (23834.02-43183.15) | 4455.60 (3257.20-5836.66) | -0.02 (-0.04 to 0.00) |
| **Oceania** | 362.86 (274.02-486.88) | 6821.62 (5163.17-8868.33) | 861.99 (639.72-1133.49) | 7046.62 (5258.47-9082.33) | 0.24 (0.17 to 0.32) |
| **Central Asia** | 4495.78 (3303.58-5858.50) | 3890.46 (2890.29-5068.60) | 3894.82 (2866.22-5146.67) | 3954.09 (2912.62-5161.83) | 0.01 (-0.02 to 0.04) |
| **Central Europe** | 2331.22 (1729.71-3107.71) | 3379.12 (2494.82-4423.50) | 4655.82 (3465.49-5986.45) | 3463.90 (2553.06-4533.86) | 0.06 (0.04 to 0.08) |
| **Eastern Europe** | 13540.65 (9769.64-17819.07) | 3120.96 (2275.14-4081.92) | 7876.49 (5747.57-10200.43) | 3170.49 (2289.66-4152.30) | 0.02 (-0.01 to 0.05) |
| **High-income Asia Pacific** | 44639.06 (32782.48-58981.20) | 3219.60 (2363.31-4214.67) | 7022.47 (5251.12-8975.23) | 3247.76 (2375.14-4239.47) | 0.01 (-0.02 to 0.03) |
| **Australasia** | 3670.48 (2709.32-4823.33) | 2811.07 (2087.71-3670.93) | 906.34 (682.76-1150.98) | 2803.25 (2080.30-3648.48) | -0.03 (-0.04 to -0.01) |
| **Western Europe** | 31046.53 (22769.39-40437.30) | 2395.05 (1786.28-3123.24) | 11847.99 (8931.24-14996.21) | 2404.86 (1790.97-3136.99) | 0.02 (0.00 to 0.03) |
| **Southern Latin America** | 7777.40 (5685.26-10115.93) | 2970.93 (2193.77-3869.12) | 2152.14 (1602.60-2788.57) | 2963.95 (2200.04-3863.44) | 0.00 (-0.02 to 0.02) |
| **High-income North America** | 35897.74 (26582.21-48091.83) | 4328.49 (3134.41-5671.09) | 16698.20 (12367.48-21352.39) | 4224.42 (3049.86-5536.13) | -0.09 (-0.12 to -0.07) |
| **Caribbean** | 23740.30 (17708.37-31547.14) | 5065.27 (3751.82-6583.66) | 2592.94 (1917.98-3351.10) | 5228.27 (3855.21-6815.48) | 0.08 (0.05 to 0.11) |
| **Andean Latin America** | 10272.00 (7743.45-13211.34) | 4498.62 (3322.38-5854.37) | 2880.37 (2107.80-3789.12) | 4490.13 (3298.29-5860.57) | -0.01 (-0.05 to 0.02) |
| **Central Latin America** | 2148.80 (1591.39-2878.02) | 6280.82 (4635.10-8164.21) | 17017.78 (12379.44-22233.78) | 6519.08 (4766.17-8493.79) | 0.10 (0.06 to 0.14) |
| **Tropical Latin America** | 17805.82 (13166.51-23884.88) | 5744.78 (4146.62-7543.43) | 14878.42 (10701.26-19485.21) | 5889.37 (4255.92-7701.51) | 0.02 (-0.03 to 0.06) |
| **North Africa and Middle East** | 13108.52 (9806.23-17367.56) | 3757.47 (2797.83-4844.18) | 23134.99 (16947.80-30356.56) | 3633.77 (2699.73-4675.87) | -0.17 (-0.23 to -0.12) |
| **South Asia** | 9866.86 (7396.20-13071.46) | 3040.75 (2225.98-3995.74) | 54130.23 (39501.79-71561.39) | 2989.08 (2182.21-3904.48) | -0.16 (-0.20 to -0.13) |
| **Central Sub-Saharan Africa** | 622.14 (460.35-812.40) | 5510.93 (4103.42-7183.52) | 5528.89 (4084.66-7375.25) | 5500.77 (4075.69-7157.30) | 0.00 (-0.04 to 0.03) |
| **Eastern Sub-Saharan Africa** | 6317.33 (4573.29-8212.48) | 10141.35 (7559.88-13080.32) | 31208.96 (23048.28-41455.55) | 10014.29 (7336.36-12908.25) | -0.12 (-0.16 to -0.07) |
| **Southern Sub-Saharan Africa** | 1373.84 (1020.52-1838.42) | 8424.86 (6211.63-10932.88) | 6299.96 (4588.35-8296.73) | 7838.36 (5733.84-10163.46) | -0.49 (-0.63 to -0.35) |
| **Western Sub-Saharan Africa** | 1617.28 (1209.99-2133.14) | 7520.49 (5610.73-9722.11) | 26723.00 (19848.35-35685.96) | 7656.26 (5652.53-9981.63) | -0.08 (-0.14 to -0.02) |

**UI, uncertainty interval; ASR, age-standardized incidence rate; CI, confidential interval; EAPC, estimated annual percentage change.**

**Table S5. Incident cases and age-standardized incidence rates of genital herpes in 1990 and 2019 and estimated annual percentage change from 1990 to 2019.**

| **Characteristics** | **1990** | | **2019** | | **1990-2019** |
| --- | --- | --- | --- | --- | --- |
|  | **Incident cases**  **No. ×10^3^ (95% UI)** | **ASR per 100,000**  **No. (95% UI)** | **Incident cases**  **No. ×10^3^ (95% UI)** | **ASR per 100,000**  **No. (95% UI)** | **EAPC**  **No. (95% CI)** |
| **Overall** | 53051.59 (45029.38-61934.04) | 960.29 (822.81-1116.81) | 80784.43 (68810.96-94200.33) | 1021.68 (869.15-1191.20) | 0.09 (0.05 to 0.13) |
| **Sex** |  |  |  |  |  |
| **Male** | 20225.30 (16909.53-23827.90) | 733.92 (620.59-864.86) | 31237.24 (26476.07-36801.24) | 778.33 (657.90-917.03) | 0.09 (0.04 to 0.14) |
| **Female** | 32826.29 (28066.35-38105.21) | 1192.43 (1027.73-1373.81) | 49547.19 (42417.73-57406.65) | 1272.13 (1084.26-1479.17) | 0.10 (0.06 to 0.13) |
| **SDI category** |  |  |  |  |  |
| **Low-SDI** | 6984.24 (5997.04-8117.01) | 1381.15 (1192.61-1585.82) | 15423.85 (13096.08-18113.34) | 1332.24 (1139.14-1537.39) | -0.19 (-0.26 to -0.11) |
| **Low-middle-SDI** | 9713.83 (8187.31-11491.74) | 887.22 (756.91-1034.59) | 17879.52 (15120.82-20828.79) | 951.22 (810.95-1108.43) | 0.20 (0.15 to 0.24) |
| **Middle-SDI** | 17684.07 (14969.97-20668.81) | 970.48 (830.83-1125.14) | 24944.85 (21164.99-29188.02) | 983.59 (834.60-1149.82) | 0.04 (0.00 to 0.09) |
| **High-middle-SDI** | 10560.19 (8891.01-12403.57) | 856.48 (728.85-1001.48) | 12906.30 (10984.14-15223.24) | 871.68 (739.91-1022.12) | -0.10 (-0.18 to -0.02) |
| **High-SDI** | 8069.12 (6890.1-9374.77) | 943.30 (808.92-1097.75) | 8141.61 (6945.86-9549.75) | 862.23 (729.35-1013.89) | -0.27 (-0.33 to -0.21) |
| **Region** |  |  |  |  |  |
| **East Asia** | 10330.94 (8538.86-12261.69) | 761.34 (637.11-905.63) | 12099.98 (10180.78-14394.45) | 761.71 (640.16-907.48) | -0.43 (-0.65 to -0.21) |
| **Southeast Asia** | 5397.90 (4591.20-6334.95) | 1103.72 (945.57-1275.96) | 7868.72 (6643.66-9246.56) | 1088.42 (915.94-1276.73) | -0.11 (-0.12 to -0.09) |
| **Oceania** | 94.14 (78.70-112.43) | 1404.58 (1186.23-1657.29) | 200.39 (168.56-236.72) | 1428.70 (1205.75-1669.20) | -0.01 (-0.17 to 0.14) |
| **Central Asia** | 469.49 (388.29-560.09) | 689.13 (579.09-816.46) | 692.11 (579.27-828.18) | 689.14 (579.50-820.98) | -0.01 (-0.01 to 0.00) |
| **Central Europe** | 621.99 (526.10-735.17) | 494.38 (418.21-583.25) | 556.25 (467.26-664.59) | 494.18 (415.42-589.23) | 0.00 (-0.04 to 0.03) |
| **Eastern Europe** | 2150.80 (1812.01-2553.29) | 935.17 (786.53-1100.05) | 1851.02 (1569.09-2189.07) | 936.47 (787.28-1100.87) | 0.01 (0.00 to 0.01) |
| **High-income Asia Pacific** | 1460.50 (1315.96-1637.98) | 797.06 (717.29-890.86) | 1136.25 (961.33-1349.95) | 680.41 (571.07-805.51) | -0.31 (-0.42 to -0.19) |
| **Australasia** | 206.02 (167.18-248.79) | 978.95 (793.60-1182.83) | 211.46 (177.78-249.82) | 794.25 (666.11-945.00) | -0.59 (-1.03 to -0.15) |
| **Western Europe** | 2867.22 (2439.42-3369.26) | 732.69 (621.91-863.74) | 2739.26 (2334.58-3227.83) | 696.52 (586.28-821.86) | -0.11 (-0.18 to -0.04) |
| **Southern Latin America** | 621.61 (568.04-679.99) | 1241.94 (1135.52-1359.36) | 822.20 (701.75-955.58) | 1216.03 (1031.26-1419.28) | -0.10 (-0.12 to -0.08) |
| **High-income North America** | 3617.02 (3045.41-4256.01) | 1227.60 (1037.68-1443.43) | 3622.26 (3095.93-4232.98) | 1077.77 (906.01-1267.90) | -0.52 (-0.62 to -0.42) |
| **Caribbean** | 615.58 (522.37-718.82) | 1629.47 (1388.57-1889.43) | 760.27 (648.32-887.50) | 1581.10 (1347.15-1842.73) | -0.11 (-0.12 to -0.10) |
| **Andean Latin America** | 705.57 (617.73-809.55) | 1744.10 (1537.00-1976.47) | 1168.68 (999.05-1351.49) | 1741.01 (1491.84-2008.59) | -0.12 (-0.18 to -0.05) |
| **Central Latin America** | 2644.87 (2282.09-3066.33) | 1534.24 (1337.35-1752.73) | 3977.09 (3390.14-4624.08) | 1502.79 (1282.43-1746.05) | 0.09 (-0.02 to 0.20) |
| **Tropical Latin America** | 3179.56 (2705.36-3700.49) | 1930.74 (1651.19-2229.14) | 4545.92 (3914.56-5251.34) | 1911.52 (1637.13-2214.89) | 0.05 (-0.09 to 0.19) |
| **North Africa and Middle East** | 2743.40 (2324.38-3250.38) | 817.35 (694.65-957.94) | 5291.00 (4457.33-6313.04) | 799.14 (675.26-948.70) | -0.06 (-0.09 to -0.03) |
| **South Asia** | 5979.32 (4980.66-7170.08) | 589.20 (494.54-704.83) | 11993.16 (10070.18-14347.08) | 626.23 (527.02-744.30) | 0.25 (0.19 to 0.32) |
| **Central Sub-Saharan Africa** | 1273.31 (1099.50-1457.05) | 2252.02 (1977.50-2538.88) | 3167.91 (2749.28-3626.10) | 2245.43 (1973.68-2530.90) | -0.01 (-0.02 to 0.00) |
| **Eastern Sub-Saharan Africa** | 3848.31 (3300.77-4454.20) | 2024.14 (1760.53-2312.94) | 8539.28 (7223.96-10055.09) | 1939.71 (1662.66-2250.26) | -0.60 (-0.77 to -0.43) |
| **Southern Sub-Saharan Africa** | 1230.30 (1055.21-1423.56) | 2166.56 (1884.67-2475.94) | 1997.94 (1730.62-2288.97) | 2275.42 (1990.26-2605.72) | 0.25 (0.15 to 0.35) |
| **Western Sub-Saharan Africa** | 2993.75 (2534.34-3511.28) | 1631.96 (1399.92-1890.87) | 7543.31 (6368.75-8870.60) | 1639.92 (1404.48-1895.52) | -0.08 (-0.12 to -0.04) |

**UI, uncertainty interval; ASR, age-standardized incidence rate; CI, confidential interval; EAPC, estimated annual percentage change.**

**Table S6. Comparison of incidence and ASR of sexually transmitted infections excluding HIV between 2017 and 2019.**

| **Regions** | **GBD 2017** | | | **GBD 2019** | | | |
| --- | --- | --- | --- | --- | --- | --- | --- |
|  | **1990** | **2017** | **1990-2017** | **1990** | **2017** | **1990-2017** | **1990-2019** |
|  | Incident No. ×10^3^  ASR per 100,000 | Incident No. ×10^3^  ASR per 100,000 | EAPC | Incident No. ×10^3^  ASR per 100,000 | Incident No. ×10^3^  ASR per 100,000 | EAPC | EAPC |
| **Global** | 539288.71  9811.46 | 767169.13  9767.90 | -0.05* | 486771.20  9323.71 | 742547.55  9373.83 | -0.04 | -0.04 |
| **Low-SDI** | 61304.61  9863.51 | 125806.50  10049.02 | 0.01 | 50472.93  11817.48 | 101797.29  10968.03 | -0.10* | -0.09* |
| **Low-middle-SDI** | 90873.76  9111.53 | 163209.96  9285.56 | 0.02 | 89265.65  8906.49 | 153549.12  8771.55 | -0.05* | -0.05* |
| **Middle-SDI** | 166988.97  10196.65 | 248035.03  9995.14 | -0.10* | 181587.51  10830.67 | 257349.20  9929.85 | -0.17* | -0.15* |
| **High-middle-SDI** | 122484.76  10321.61 | 167988.25  10150.87 | -0.08* | 112059.93  9238.83 | 143632.54  8922.29 | -0.24* | -0.20* |
| **High-SDI** | 94883.61  9400.57 | 100168.29  9373.17 | -0.04 | 53073.66  5940.18 | 64649.16  6039.27 | 0.06* | 0.06* |

ASR, age-standardized incidence rate; EAPC, estimated annual percentage change; GBD, Global Burden of Disease Study.

*95% confidential interval does not include 0.

**Table S7. The change of STIs excluding HIV cases between 1990 and 2019 at national level.**

| **Region** | **STIs** | **Case in 1990** | **Case in 2019** | **Change in absolute number (%)** | **EAPC**  **No. (95% CI)** |
| --- | --- | --- | --- | --- | --- |
| Afghanistan | Sexually transmitted infections excluding HIV | 733389.49 | 2743118.33 | 274.03 | 0.04(0.03 to 0.05) |
| Afghanistan | Syphilis | 9012.74 | 37632.22 | 317.54 | 0.25(0.2 to 0.3) |
| Afghanistan | Chlamydial infection | 247027.80 | 999085.25 | 304.44 | 0.08(0.06 to 0.1) |
| Afghanistan | Gonococcal infection | 132631.94 | 477828.83 | 260.27 | -0.11(-0.16 to -0.05) |
| Afghanistan | Trichomoniasis | 269030.51 | 954510.54 | 254.80 | 0.07(0.06 to 0.09) |
| Afghanistan | Genital herpes | 75686.51 | 274061.50 | 262.10 | -0.11(-0.13 to -0.09) |
| Albania | Sexually transmitted infections excluding HIV | 306690.78 | 253186.03 | -17.45 | -0.1(-0.12 to -0.09) |
| Albania | Syphilis | 1355.68 | 1075.14 | -20.69 | -0.1(-0.15 to -0.06) |
| Albania | Chlamydial infection | 115025.82 | 89547.09 | -22.15 | -0.08(-0.1 to -0.05) |
| Albania | Gonococcal infection | 72390.17 | 49621.14 | -31.45 | -0.27(-0.32 to -0.21) |
| Albania | Trichomoniasis | 100297.16 | 98786.18 | -1.51 | -0.05(-0.07 to -0.04) |
| Albania | Genital herpes | 17621.95 | 14156.48 | -19.67 | 0(-0.02 to 0.01) |
| Algeria | Sexually transmitted infections excluding HIV | 1793900.72 | 3578791.88 | 99.50 | -0.05(-0.12 to 0.02) |
| Algeria | Syphilis | 20292.31 | 36445.44 | 79.60 | -0.24(-0.4 to -0.09) |
| Algeria | Chlamydial infection | 725764.87 | 1301730.55 | 79.36 | -0.02(-0.22 to 0.18) |
| Algeria | Gonococcal infection | 206956.28 | 311872.01 | 50.69 | -0.15(-0.17 to -0.13) |
| Algeria | Trichomoniasis | 645393.07 | 1575437.84 | 144.11 | -0.05(-0.07 to -0.04) |
| Algeria | Genital herpes | 195494.20 | 353306.04 | 80.72 | -0.01(-0.01 to 0) |
| American Samoa | Sexually transmitted infections excluding HIV | 5560.85 | 6613.99 | 18.94 | 0.02(0 to 0.03) |
| American Samoa | Syphilis | 153.22 | 169.17 | 10.41 | -0.03(-0.05 to -0.01) |
| American Samoa | Chlamydial infection | 1407.84 | 1571.72 | 11.64 | 0.01(-0.01 to 0.03) |
| American Samoa | Gonococcal infection | 563.23 | 591.12 | 4.95 | -0.31(-0.36 to -0.26) |
| American Samoa | Trichomoniasis | 2752.98 | 3536.49 | 28.46 | 0.07(0.04 to 0.1) |
| American Samoa | Genital herpes | 683.58 | 745.49 | 9.06 | 0.02(0 to 0.03) |
| Andorra | Sexually transmitted infections excluding HIV | 2390.82 | 3552.18 | 48.58 | -0.07(-0.08 to -0.06) |
| Andorra | Syphilis | 46.25 | 54.82 | 18.53 | -0.16(-0.18 to -0.13) |
| Andorra | Chlamydial infection | 228.56 | 313.88 | 37.33 | -0.04(-0.05 to -0.03) |
| Andorra | Gonococcal infection | 77.61 | 104.01 | 34.02 | -0.06(-0.08 to -0.04) |
| Andorra | Trichomoniasis | 1604.59 | 2523.62 | 57.28 | -0.11(-0.12 to -0.09) |
| Andorra | Genital herpes | 433.81 | 555.85 | 28.13 | 0.03(0.02 to 0.05) |
| Angola | Sexually transmitted infections excluding HIV | 1107300.69 | 3097370.87 | 179.72 | -0.15(-0.17 to -0.13) |
| Angola | Syphilis | 117220.07 | 307093.72 | 161.98 | -0.5(-0.55 to -0.45) |
| Angola | Chlamydial infection | 197365.42 | 541926.44 | 174.58 | -0.16(-0.17 to -0.15) |
| Angola | Gonococcal infection | 133377.09 | 354370.42 | 165.69 | -0.37(-0.39 to -0.35) |
| Angola | Trichomoniasis | 421270.82 | 1189733.86 | 182.42 | -0.11(-0.15 to -0.07) |
| Angola | Genital herpes | 238067.29 | 704246.43 | 195.82 | 0.08(0.05 to 0.1) |
| Antigua and Barbuda | Sexually transmitted infections excluding HIV | 7618.09 | 12603.39 | 65.44 | 0.01(0 to 0.03) |
| Antigua and Barbuda | Syphilis | 84.54 | 128.93 | 52.50 | -0.04(-0.09 to 0.02) |
| Antigua and Barbuda | Chlamydial infection | 2786.77 | 4436.18 | 59.19 | 0(-0.01 to 0) |
| Antigua and Barbuda | Gonococcal infection | 820.80 | 1103.41 | 34.43 | -0.21(-0.27 to -0.15) |
| Antigua and Barbuda | Trichomoniasis | 2852.85 | 5417.06 | 89.88 | 0.09(0.06 to 0.12) |
| Antigua and Barbuda | Genital herpes | 1073.13 | 1517.81 | 41.44 | -0.03(-0.05 to 0) |
| Argentina | Sexually transmitted infections excluding HIV | 1812139.92 | 2688505.81 | 48.36 | 0(-0.02 to 0.02) |
| Argentina | Syphilis | 42108.33 | 70779.03 | 68.09 | 0.54(0.21 to 0.87) |
| Argentina | Chlamydial infection | 278142.02 | 430639.49 | 54.83 | 0.17(0.13 to 0.2) |
| Argentina | Gonococcal infection | 141958.12 | 198772.20 | 40.02 | -0.01(-0.03 to 0) |
| Argentina | Trichomoniasis | 942315.70 | 1427568.93 | 51.50 | -0.01(-0.04 to 0.01) |
| Argentina | Genital herpes | 407615.75 | 560746.16 | 37.57 | -0.14(-0.16 to -0.11) |
| Armenia | Sexually transmitted infections excluding HIV | 435741.40 | 387369.52 | -11.10 | -0.16(-0.19 to -0.13) |
| Armenia | Syphilis | 1797.25 | 1397.25 | -22.26 | -0.7(-0.8 to -0.61) |
| Armenia | Chlamydial infection | 187853.22 | 168477.66 | -10.31 | -0.04(-0.06 to -0.03) |
| Armenia | Gonococcal infection | 91067.23 | 58739.75 | -35.50 | -0.74(-0.84 to -0.65) |
| Armenia | Trichomoniasis | 130230.09 | 136874.67 | 5.10 | 0.01(-0.02 to 0.05) |
| Armenia | Genital herpes | 24793.61 | 21880.19 | -11.75 | 0(-0.01 to 0.01) |
| Australia | Sexually transmitted infections excluding HIV | 934657.83 | 1263425.89 | 35.18 | -0.16(-0.25 to -0.06) |
| Australia | Syphilis | 11626.25 | 14987.25 | 28.91 | -0.11(-0.13 to -0.09) |
| Australia | Chlamydial infection | 171588.50 | 231743.43 | 35.06 | -0.07(-0.12 to -0.02) |
| Australia | Gonococcal infection | 54580.45 | 67212.43 | 23.14 | -0.19(-0.21 to -0.16) |
| Australia | Trichomoniasis | 513279.03 | 763476.60 | 48.74 | -0.01(-0.02 to 0) |
| Australia | Genital herpes | 183583.60 | 186006.18 | 1.32 | -0.69(-1.19 to -0.18) |
| Austria | Sexually transmitted infections excluding HIV | 299167.47 | 343271.43 | 14.74 | 0(-0.01 to 0.01) |
| Austria | Syphilis | 5491.33 | 5496.92 | 0.10 | -0.08(-0.1 to -0.05) |
| Austria | Chlamydial infection | 29129.55 | 31279.46 | 7.38 | 0.01(0 to 0.02) |
| Austria | Gonococcal infection | 10080.60 | 10010.46 | -0.70 | -0.11(-0.17 to -0.05) |
| Austria | Trichomoniasis | 202317.65 | 242806.87 | 20.01 | 0.01(-0.01 to 0.03) |
| Austria | Genital herpes | 52148.33 | 53677.71 | 2.93 | -0.03(-0.05 to -0.01) |
| Azerbaijan | Sexually transmitted infections excluding HIV | 933489.41 | 1444453.25 | 54.74 | -0.15(-0.17 to -0.13) |
| Azerbaijan | Syphilis | 4280.59 | 5925.59 | 38.43 | -0.5(-0.58 to -0.42) |
| Azerbaijan | Chlamydial infection | 401040.64 | 634339.79 | 58.17 | -0.09(-0.11 to -0.07) |
| Azerbaijan | Gonococcal infection | 213716.37 | 244573.67 | 14.44 | -0.5(-0.56 to -0.45) |
| Azerbaijan | Trichomoniasis | 262004.56 | 478622.95 | 82.68 | -0.03(-0.06 to 0) |
| Azerbaijan | Genital herpes | 52447.25 | 80991.24 | 54.42 | -0.02(-0.03 to -0.01) |
| Bahamas | Sexually transmitted infections excluding HIV | 34048.70 | 53539.26 | 57.24 | 0.02(0 to 0.03) |
| Bahamas | Syphilis | 509.43 | 739.27 | 45.12 | 0.4(0.29 to 0.51) |
| Bahamas | Chlamydial infection | 12554.68 | 18920.50 | 50.70 | 0(0 to 0) |
| Bahamas | Gonococcal infection | 3712.54 | 4682.47 | 26.13 | -0.27(-0.33 to -0.2) |
| Bahamas | Trichomoniasis | 12386.91 | 22597.38 | 82.43 | 0.08(0.05 to 0.11) |
| Bahamas | Genital herpes | 4885.14 | 6599.64 | 35.10 | 0.01(0 to 0.03) |
| Bahrain | Sexually transmitted infections excluding HIV | 50174.98 | 168207.39 | 235.24 | 0.03(0.01 to 0.05) |
| Bahrain | Syphilis | 608.81 | 1740.30 | 185.85 | 0.08(0.03 to 0.13) |
| Bahrain | Chlamydial infection | 18920.25 | 56976.32 | 201.14 | 0.04(0 to 0.09) |
| Bahrain | Gonococcal infection | 6693.20 | 15348.37 | 129.31 | -0.16(-0.23 to -0.08) |
| Bahrain | Trichomoniasis | 19473.57 | 81405.98 | 318.03 | 0.09(0.07 to 0.11) |
| Bahrain | Genital herpes | 4479.14 | 12736.43 | 184.35 | -0.06(-0.09 to -0.03) |
| Bangladesh | Sexually transmitted infections excluding HIV | 5762137.24 | 10213258.87 | 77.25 | -0.19(-0.21 to -0.17) |
| Bangladesh | Syphilis | 257418.29 | 370366.56 | 43.88 | -1.13(-1.34 to -0.93) |
| Bangladesh | Chlamydial infection | 1810076.57 | 3217844.15 | 77.77 | -0.02(-0.03 to -0.01) |
| Bangladesh | Gonococcal infection | 871432.31 | 1155534.07 | 32.60 | -0.68(-0.75 to -0.62) |
| Bangladesh | Trichomoniasis | 2269030.85 | 4489302.83 | 97.85 | -0.13(-0.16 to -0.11) |
| Bangladesh | Genital herpes | 554179.23 | 980211.26 | 76.88 | 0.02(0.02 to 0.03) |
| Barbados | Sexually transmitted infections excluding HIV | 36944.32 | 42534.20 | 15.13 | 0.01(-0.05 to 0.08) |
| Barbados | Syphilis | 305.38 | 327.35 | 7.19 | -0.04(-0.06 to -0.03) |
| Barbados | Chlamydial infection | 15347.83 | 16640.81 | 8.42 | 0(-0.14 to 0.15) |
| Barbados | Gonococcal infection | 3613.02 | 3389.38 | -6.19 | -0.16(-0.29 to -0.03) |
| Barbados | Trichomoniasis | 13161.31 | 17732.48 | 34.73 | 0.07(0.04 to 0.1) |
| Barbados | Genital herpes | 4516.78 | 4444.19 | -1.61 | 0.02(0.01 to 0.04) |
| Belarus | Sexually transmitted infections excluding HIV | 1039542.40 | 917276.48 | -11.76 | -0.1(-0.11 to -0.08) |
| Belarus | Syphilis | 4823.22 | 3976.83 | -17.55 | -0.44(-0.51 to -0.37) |
| Belarus | Chlamydial infection | 408734.19 | 365016.30 | -10.70 | 0(-0.01 to 0.01) |
| Belarus | Gonococcal infection | 197472.76 | 134737.12 | -31.77 | -0.55(-0.61 to -0.49) |
| Belarus | Trichomoniasis | 328815.58 | 331946.59 | 0.95 | 0.05(0.02 to 0.08) |
| Belarus | Genital herpes | 99696.65 | 81599.64 | -18.15 | 0(-0.01 to 0) |
| Belgium | Sexually transmitted infections excluding HIV | 376809.85 | 412535.70 | 9.48 | -0.03(-0.1 to 0.04) |
| Belgium | Syphilis | 6857.57 | 6745.23 | -1.64 | -0.13(-0.15 to -0.12) |
| Belgium | Chlamydial infection | 46296.68 | 48765.56 | 5.33 | -0.07(-0.27 to 0.13) |
| Belgium | Gonococcal infection | 12907.87 | 12259.40 | -5.02 | -0.37(-0.39 to -0.34) |
| Belgium | Trichomoniasis | 241753.17 | 273719.57 | 13.22 | -0.06(-0.22 to 0.09) |
| Belgium | Genital herpes | 68994.55 | 71045.94 | 2.97 | 0.16(0.08 to 0.24) |
| Belize | Sexually transmitted infections excluding HIV | 19431.88 | 54120.69 | 178.51 | 0.01(0 to 0.03) |
| Belize | Syphilis | 229.16 | 586.08 | 155.75 | -0.15(-0.2 to -0.1) |
| Belize | Chlamydial infection | 7182.53 | 19808.38 | 175.79 | 0.01(0.01 to 0.01) |
| Belize | Gonococcal infection | 2324.74 | 5478.23 | 135.65 | -0.21(-0.28 to -0.13) |
| Belize | Trichomoniasis | 6714.64 | 20752.71 | 209.07 | 0.07(0.04 to 0.09) |
| Belize | Genital herpes | 2980.81 | 7495.29 | 151.45 | 0.03(0.02 to 0.05) |
| Benin | Sexually transmitted infections excluding HIV | 396923.39 | 1147001.91 | 188.97 | 0.14(0.04 to 0.24) |
| Benin | Syphilis | 10542.05 | 27308.40 | 159.04 | -1.11(-1.49 to -0.72) |
| Benin | Chlamydial infection | 63954.54 | 185102.94 | 189.43 | 0.18(0.1 to 0.25) |
| Benin | Gonococcal infection | 61659.54 | 179022.78 | 190.34 | -0.18(-0.33 to -0.02) |
| Benin | Trichomoniasis | 193283.69 | 564073.37 | 191.84 | 0.21(0.02 to 0.39) |
| Benin | Genital herpes | 67483.57 | 191494.41 | 183.76 | 0.32(0.12 to 0.51) |
| Bermuda | Sexually transmitted infections excluding HIV | 8728.45 | 8133.06 | -6.82 | 0.01(0 to 0.03) |
| Bermuda | Syphilis | 99.94 | 85.13 | -14.82 | 0.05(0.02 to 0.08) |
| Bermuda | Chlamydial infection | 3164.38 | 2674.05 | -15.50 | 0(-0.01 to 0) |
| Bermuda | Gonococcal infection | 727.27 | 541.88 | -25.49 | -0.32(-0.38 to -0.26) |
| Bermuda | Trichomoniasis | 3633.61 | 3977.07 | 9.45 | 0.1(0.08 to 0.12) |
| Bermuda | Genital herpes | 1103.26 | 854.94 | -22.51 | 0.02(0 to 0.04) |
| Bhutan | Sexually transmitted infections excluding HIV | 33476.91 | 51542.59 | 53.96 | -0.1(-0.12 to -0.08) |
| Bhutan | Syphilis | 1938.90 | 2651.86 | 36.77 | -0.47(-0.64 to -0.3) |
| Bhutan | Chlamydial infection | 10458.62 | 16032.09 | 53.29 | -0.06(-0.07 to -0.05) |
| Bhutan | Gonococcal infection | 4835.93 | 5670.99 | 17.27 | -0.31(-0.34 to -0.28) |
| Bhutan | Trichomoniasis | 12990.08 | 22326.77 | 71.88 | -0.05(-0.07 to -0.04) |
| Bhutan | Genital herpes | 3253.39 | 4860.88 | 49.41 | -0.01(-0.02 to 0.01) |
| Bolivia (Plurinational State of) | Sexually transmitted infections excluding HIV | 503266.85 | 1085006.63 | 115.59 | -0.03(-0.05 to 0) |
| Bolivia (Plurinational State of) | Syphilis | 15492.26 | 30201.30 | 94.94 | -0.52(-0.65 to -0.39) |
| Bolivia (Plurinational State of) | Chlamydial infection | 135930.75 | 293028.70 | 115.57 | 0(-0.03 to 0.02) |
| Bolivia (Plurinational State of) | Gonococcal infection | 17412.72 | 32994.41 | 89.48 | -0.11(-0.13 to -0.09) |
| Bolivia (Plurinational State of) | Trichomoniasis | 221857.04 | 509645.38 | 129.72 | -0.01(-0.05 to 0.03) |
| Bolivia (Plurinational State of) | Genital herpes | 112574.07 | 219136.84 | 94.66 | -0.03(-0.04 to -0.03) |
| Bosnia and Herzegovina | Sexually transmitted infections excluding HIV | 447761.58 | 303461.33 | -32.23 | -0.03(-0.04 to -0.02) |
| Bosnia and Herzegovina | Syphilis | 1948.12 | 1260.72 | -35.29 | 0.03(0 to 0.06) |
| Bosnia and Herzegovina | Chlamydial infection | 168144.52 | 106202.54 | -36.84 | -0.04(-0.05 to -0.03) |
| Bosnia and Herzegovina | Gonococcal infection | 93305.79 | 51633.24 | -44.66 | -0.16(-0.19 to -0.13) |
| Bosnia and Herzegovina | Trichomoniasis | 158530.18 | 127492.25 | -19.58 | 0.05(0.02 to 0.07) |
| Bosnia and Herzegovina | Genital herpes | 25832.96 | 16872.57 | -34.69 | -0.01(-0.02 to 0.01) |
| Botswana | Sexually transmitted infections excluding HIV | 184262.39 | 396646.58 | 115.26 | -0.24(-0.29 to -0.18) |
| Botswana | Syphilis | 11030.68 | 15556.01 | 41.02 | -1.89(-2.44 to -1.33) |
| Botswana | Chlamydial infection | 38300.43 | 86142.23 | 124.91 | -0.18(-0.38 to 0.02) |
| Botswana | Gonococcal infection | 34377.93 | 60985.04 | 77.40 | -0.07(-0.19 to 0.06) |
| Botswana | Trichomoniasis | 71140.51 | 172062.26 | 141.86 | -0.3(-0.46 to -0.13) |
| Botswana | Genital herpes | 29412.84 | 61901.04 | 110.46 | 0.19(0.04 to 0.34) |
| Brazil | Sexually transmitted infections excluding HIV | 18462834.71 | 31101136.38 | 68.45 | -0.07(-0.13 to 0) |
| Brazil | Syphilis | 182995.43 | 315944.76 | 72.65 | -0.81(-1.73 to 0.12) |
| Brazil | Chlamydial infection | 5992731.12 | 9836525.52 | 64.14 | -0.17(-0.31 to -0.04) |
| Brazil | Gonococcal infection | 1541663.22 | 2047542.87 | 32.81 | -0.29(-0.49 to -0.09) |
| Brazil | Trichomoniasis | 7641883.21 | 14500391.94 | 89.75 | 0.02(-0.03 to 0.06) |
| Brazil | Genital herpes | 3103561.74 | 4400731.30 | 41.80 | 0.05(-0.09 to 0.19) |
| Brunei Darussalam | Sexually transmitted infections excluding HIV | 15067.44 | 29837.56 | 98.03 | 0.01(-0.01 to 0.03) |
| Brunei Darussalam | Syphilis | 224.02 | 404.66 | 80.64 | -0.02(-0.07 to 0.03) |
| Brunei Darussalam | Chlamydial infection | 3167.42 | 5903.00 | 86.37 | -0.02(-0.04 to 0.01) |
| Brunei Darussalam | Gonococcal infection | 1993.96 | 3453.52 | 73.20 | -0.03(-0.05 to 0) |
| Brunei Darussalam | Trichomoniasis | 7603.67 | 16314.75 | 114.56 | 0.04(0 to 0.07) |
| Brunei Darussalam | Genital herpes | 2078.37 | 3761.63 | 80.99 | 0(-0.04 to 0.03) |
| Bulgaria | Sexually transmitted infections excluding HIV | 842967.88 | 648535.10 | -23.07 | -0.11(-0.15 to -0.07) |
| Bulgaria | Syphilis | 3136.20 | 2328.24 | -25.76 | -0.03(-0.05 to -0.01) |
| Bulgaria | Chlamydial infection | 322392.05 | 242789.96 | -24.69 | -0.15(-0.21 to -0.1) |
| Bulgaria | Gonococcal infection | 144777.87 | 90309.03 | -37.62 | -0.17(-0.23 to -0.11) |
| Bulgaria | Trichomoniasis | 314409.67 | 270142.88 | -14.08 | 0.05(0.03 to 0.07) |
| Bulgaria | Genital herpes | 58252.09 | 42964.99 | -26.24 | -0.42(-0.58 to -0.27) |
| Burkina Faso | Sexually transmitted infections excluding HIV | 1019694.25 | 2464972.32 | 141.74 | -0.36(-0.45 to -0.27) |
| Burkina Faso | Syphilis | 26137.33 | 71083.76 | 171.96 | 0.35(0.23 to 0.47) |
| Burkina Faso | Chlamydial infection | 138790.57 | 363741.26 | 162.08 | -0.07(-0.09 to -0.05) |
| Burkina Faso | Gonococcal infection | 136855.55 | 328906.00 | 140.33 | -0.26(-0.34 to -0.18) |
| Burkina Faso | Trichomoniasis | 584289.09 | 1356695.80 | 132.20 | -0.51(-0.66 to -0.36) |
| Burkina Faso | Genital herpes | 133621.71 | 344545.49 | 157.85 | -0.17(-0.21 to -0.13) |
| Burundi | Sexually transmitted infections excluding HIV | 728117.89 | 1626518.54 | 123.39 | -0.02(-0.05 to 0) |
| Burundi | Syphilis | 20717.70 | 38261.51 | 84.68 | -0.85(-1.15 to -0.55) |
| Burundi | Chlamydial infection | 151415.25 | 337271.43 | 122.75 | 0.02(0.01 to 0.03) |
| Burundi | Gonococcal infection | 71664.28 | 155100.00 | 116.43 | -0.16(-0.19 to -0.13) |
| Burundi | Trichomoniasis | 373564.70 | 850204.89 | 127.59 | 0.02(-0.03 to 0.08) |
| Burundi | Genital herpes | 110755.96 | 245680.70 | 121.82 | -0.08(-0.1 to -0.05) |
| Cabo Verde | Sexually transmitted infections excluding HIV | 32718.87 | 72900.76 | 122.81 | -0.02(-0.05 to 0.01) |
| Cabo Verde | Syphilis | 1634.71 | 3504.21 | 114.36 | 0.17(0.1 to 0.24) |
| Cabo Verde | Chlamydial infection | 5248.94 | 11314.39 | 115.56 | -0.03(-0.05 to -0.02) |
| Cabo Verde | Gonococcal infection | 4011.09 | 7207.63 | 79.69 | 0.04(0 to 0.07) |
| Cabo Verde | Trichomoniasis | 16535.89 | 41032.68 | 148.14 | -0.02(-0.08 to 0.03) |
| Cabo Verde | Genital herpes | 5288.25 | 9841.84 | 86.11 | -0.08(-0.09 to -0.07) |
| Cambodia | Sexually transmitted infections excluding HIV | 950705.82 | 1934336.08 | 103.46 | -0.07(-0.09 to -0.04) |
| Cambodia | Syphilis | 3940.02 | 7560.11 | 91.88 | -0.29(-0.43 to -0.14) |
| Cambodia | Chlamydial infection | 386308.40 | 782722.42 | 102.62 | -0.04(-0.06 to -0.01) |
| Cambodia | Gonococcal infection | 131052.82 | 228686.05 | 74.50 | -0.3(-0.38 to -0.22) |
| Cambodia | Trichomoniasis | 317191.67 | 707634.80 | 123.09 | -0.03(-0.05 to -0.01) |
| Cambodia | Genital herpes | 112212.91 | 207732.69 | 85.12 | -0.03(-0.04 to -0.03) |
| Cameroon | Sexually transmitted infections excluding HIV | 1224027.39 | 3752577.94 | 206.58 | -0.27(-0.34 to -0.19) |
| Cameroon | Syphilis | 76776.23 | 186334.01 | 142.70 | -0.84(-0.96 to -0.73) |
| Cameroon | Chlamydial infection | 175904.99 | 551576.35 | 213.56 | 0.01(-0.08 to 0.09) |
| Cameroon | Gonococcal infection | 201747.59 | 601974.90 | 198.38 | -0.29(-0.36 to -0.23) |
| Cameroon | Trichomoniasis | 578309.20 | 1810779.22 | 213.12 | -0.15(-0.2 to -0.09) |
| Cameroon | Genital herpes | 191289.38 | 601913.46 | 214.66 | -0.74(-1.04 to -0.43) |
| Canada | Sexually transmitted infections excluding HIV | 1628214.13 | 2049791.98 | 25.89 | 0.03(0 to 0.07) |
| Canada | Syphilis | 15541.75 | 17766.08 | 14.31 | -0.09(-0.21 to 0.02) |
| Canada | Chlamydial infection | 100768.76 | 117374.02 | 16.48 | -0.84(-1.36 to -0.32) |
| Canada | Gonococcal infection | 79263.79 | 88275.00 | 11.37 | -0.11(-0.13 to -0.1) |
| Canada | Trichomoniasis | 1232268.85 | 1596767.32 | 29.58 | -0.02(-0.03 to 0) |
| Canada | Genital herpes | 200370.97 | 229609.55 | 14.59 | 0.74(0.43 to 1.05) |
| Central African Republic | Sexually transmitted infections excluding HIV | 302085.45 | 613473.66 | 103.08 | -0.04(-0.05 to -0.02) |
| Central African Republic | Syphilis | 31379.35 | 66910.21 | 113.23 | -0.22(-0.3 to -0.14) |
| Central African Republic | Chlamydial infection | 55167.75 | 110689.66 | 100.64 | -0.04(-0.07 to -0.02) |
| Central African Republic | Gonococcal infection | 38255.33 | 75836.27 | 98.24 | -0.1(-0.15 to -0.06) |
| Central African Republic | Trichomoniasis | 111861.72 | 229012.14 | 104.73 | 0.06(0.02 to 0.09) |
| Central African Republic | Genital herpes | 65421.29 | 131025.38 | 100.28 | -0.11(-0.16 to -0.06) |
| Chad | Sexually transmitted infections excluding HIV | 552243.08 | 1455064.58 | 163.48 | -0.06(-0.08 to -0.03) |
| Chad | Syphilis | 26053.07 | 67928.69 | 160.73 | -0.39(-0.52 to -0.26) |
| Chad | Chlamydial infection | 90540.87 | 239625.52 | 164.66 | -0.05(-0.07 to -0.03) |
| Chad | Gonococcal infection | 74455.16 | 193713.28 | 160.17 | -0.27(-0.3 to -0.23) |
| Chad | Trichomoniasis | 287243.26 | 748672.75 | 160.64 | 0.01(-0.04 to 0.06) |
| Chad | Genital herpes | 73950.72 | 205124.34 | 177.38 | -0.07(-0.19 to 0.05) |
| Chile | Sexually transmitted infections excluding HIV | 761384.86 | 1103862.84 | 44.98 | -0.03(-0.04 to -0.02) |
| Chile | Syphilis | 12671.11 | 14149.95 | 11.67 | -1.13(-1.5 to -0.76) |
| Chile | Chlamydial infection | 124378.10 | 176351.47 | 41.79 | -0.01(-0.03 to 0) |
| Chile | Gonococcal infection | 63667.87 | 76286.76 | 19.82 | -0.21(-0.24 to -0.18) |
| Chile | Trichomoniasis | 383290.05 | 615339.44 | 60.54 | 0.01(-0.01 to 0.02) |
| Chile | Genital herpes | 177377.74 | 221735.23 | 25.01 | -0.01(-0.02 to 0) |
| China | Sexually transmitted infections excluding HIV | 132930710.74 | 172833232.59 | 30.02 | -0.42(-0.59 to -0.25) |
| China | Syphilis | 1183220.42 | 1411512.57 | 19.29 | -0.09(-0.22 to 0.05) |
| China | Chlamydial infection | 51171505.50 | 66494724.66 | 29.94 | -0.66(-1.09 to -0.23) |
| China | Gonococcal infection | 17761059.91 | 14506774.59 | -18.32 | -0.55(-0.6 to -0.5) |
| China | Trichomoniasis | 52823552.06 | 78716454.49 | 49.02 | -0.18(-0.26 to -0.1) |
| China | Genital herpes | 9991372.84 | 11703766.28 | 17.14 | -0.44(-0.67 to -0.22) |
| Colombia | Sexually transmitted infections excluding HIV | 3649780.15 | 5997994.59 | 64.34 | -0.01(-0.06 to 0.03) |
| Colombia | Syphilis | 64792.83 | 94041.09 | 45.14 | 0.01(-0.03 to 0.04) |
| Colombia | Chlamydial infection | 976244.42 | 1581686.30 | 62.02 | 0.22(0.16 to 0.28) |
| Colombia | Gonococcal infection | 183803.35 | 253976.64 | 38.18 | -0.03(-0.07 to 0) |
| Colombia | Trichomoniasis | 1714259.72 | 3207193.65 | 87.09 | 0.11(0.07 to 0.16) |
| Colombia | Genital herpes | 710679.83 | 861096.91 | 21.17 | -0.8(-0.93 to -0.66) |
| Comoros | Sexually transmitted infections excluding HIV | 60981.62 | 116433.79 | 90.93 | -0.06(-0.1 to -0.02) |
| Comoros | Syphilis | 2471.94 | 3347.36 | 35.41 | -1.22(-1.4 to -1.05) |
| Comoros | Chlamydial infection | 12159.62 | 23408.77 | 92.51 | 0.04(0.03 to 0.05) |
| Comoros | Gonococcal infection | 6149.59 | 9714.20 | 57.96 | -0.16(-0.19 to -0.13) |
| Comoros | Trichomoniasis | 30934.23 | 64490.90 | 108.48 | -0.02(-0.09 to 0.05) |
| Comoros | Genital herpes | 9266.24 | 15472.56 | 66.98 | -0.03(-0.04 to -0.01) |
| Congo | Sexually transmitted infections excluding HIV | 252801.77 | 621898.70 | 146.00 | -0.03(-0.04 to -0.01) |
| Congo | Syphilis | 24277.80 | 55119.49 | 127.04 | -0.22(-0.25 to -0.18) |
| Congo | Chlamydial infection | 44551.09 | 111520.50 | 150.32 | -0.05(-0.08 to -0.03) |
| Congo | Gonococcal infection | 31111.43 | 64777.30 | 108.21 | -0.23(-0.26 to -0.19) |
| Congo | Trichomoniasis | 93739.71 | 263477.13 | 181.07 | 0.05(0.01 to 0.08) |
| Congo | Genital herpes | 59121.75 | 127004.27 | 114.82 | 0.01(-0.01 to 0.02) |
| Cook Islands | Sexually transmitted infections excluding HIV | 2176.57 | 2216.28 | 1.82 | 0.13(0.1 to 0.16) |
| Cook Islands | Syphilis | 58.20 | 49.01 | -15.79 | -0.07(-0.09 to -0.04) |
| Cook Islands | Chlamydial infection | 527.19 | 512.66 | -2.76 | 0.25(0.22 to 0.29) |
| Cook Islands | Gonococcal infection | 203.25 | 158.33 | -22.10 | -0.14(-0.16 to -0.13) |
| Cook Islands | Trichomoniasis | 1125.69 | 1277.97 | 13.53 | 0.15(0.1 to 0.19) |
| Cook Islands | Genital herpes | 262.24 | 218.31 | -16.75 | 0.01(0 to 0.03) |
| Costa Rica | Sexually transmitted infections excluding HIV | 352673.38 | 640060.32 | 81.49 | 0.03(0 to 0.05) |
| Costa Rica | Syphilis | 3590.52 | 5840.69 | 62.67 | -0.02(-0.03 to -0.01) |
| Costa Rica | Chlamydial infection | 110094.31 | 187055.41 | 69.90 | -0.02(-0.02 to -0.01) |
| Costa Rica | Gonococcal infection | 24955.03 | 36264.19 | 45.32 | -0.12(-0.17 to -0.07) |
| Costa Rica | Trichomoniasis | 158638.54 | 328657.37 | 107.17 | 0.13(0.09 to 0.17) |
| Costa Rica | Genital herpes | 55394.98 | 82242.66 | 48.47 | -0.19(-0.25 to -0.12) |
| Croatia | Sexually transmitted infections excluding HIV | 479189.33 | 386828.78 | -19.27 | 0.06(0.01 to 0.11) |
| Croatia | Syphilis | 2021.50 | 1577.02 | -21.99 | -0.01(-0.03 to 0.01) |
| Croatia | Chlamydial infection | 181572.68 | 140764.58 | -22.47 | 0.16(0.05 to 0.27) |
| Croatia | Gonococcal infection | 84763.91 | 59478.57 | -29.83 | -0.17(-0.2 to -0.13) |
| Croatia | Trichomoniasis | 181879.73 | 162316.79 | -10.76 | 0.05(0.02 to 0.08) |
| Croatia | Genital herpes | 28951.52 | 22691.83 | -21.62 | 0.2(0.02 to 0.37) |
| Cuba | Sexually transmitted infections excluding HIV | 1464704.19 | 1490307.20 | 1.75 | 0.02(0 to 0.03) |
| Cuba | Syphilis | 16482.65 | 14270.34 | -13.42 | -0.4(-0.64 to -0.16) |
| Cuba | Chlamydial infection | 534012.99 | 503855.41 | -5.65 | 0(-0.01 to 0) |
| Cuba | Gonococcal infection | 145966.16 | 110661.50 | -24.19 | -0.2(-0.3 to -0.1) |
| Cuba | Trichomoniasis | 567294.48 | 695878.83 | 22.67 | 0.1(0.07 to 0.13) |
| Cuba | Genital herpes | 200947.91 | 165641.11 | -17.57 | 0(-0.02 to 0.01) |
| Cyprus | Sexually transmitted infections excluding HIV | 28988.58 | 54639.73 | 88.49 | -0.02(-0.03 to -0.01) |
| Cyprus | Syphilis | 547.35 | 912.42 | 66.70 | -0.12(-0.14 to -0.11) |
| Cyprus | Chlamydial infection | 2876.18 | 5077.02 | 76.52 | -0.03(-0.04 to -0.02) |
| Cyprus | Gonococcal infection | 1018.65 | 1626.10 | 59.63 | -0.12(-0.16 to -0.08) |
| Cyprus | Trichomoniasis | 19007.37 | 37630.89 | 97.98 | -0.03(-0.04 to -0.01) |
| Cyprus | Genital herpes | 5539.02 | 9393.29 | 69.58 | 0.02(0.01 to 0.02) |
| Czechia | Sexually transmitted infections excluding HIV | 950570.19 | 954181.73 | 0.38 | -0.01(-0.02 to 0) |
| Czechia | Syphilis | 4115.10 | 3973.30 | -3.45 | 0.02(0.01 to 0.04) |
| Czechia | Chlamydial infection | 351910.21 | 343013.30 | -2.53 | -0.02(-0.03 to 0) |
| Czechia | Gonococcal infection | 181503.25 | 139203.69 | -23.31 | -0.2(-0.23 to -0.17) |
| Czechia | Trichomoniasis | 373222.49 | 420337.78 | 12.62 | 0.06(0.05 to 0.08) |
| Czechia | Genital herpes | 39819.14 | 47653.68 | 19.68 | 0.26(0.1 to 0.42) |
| Côte d'Ivoire | Sexually transmitted infections excluding HIV | 1205719.78 | 2855713.27 | 136.85 | -0.05(-0.08 to -0.01) |
| Côte d'Ivoire | Syphilis | 49195.71 | 99260.80 | 101.77 | -0.23(-0.37 to -0.09) |
| Côte d'Ivoire | Chlamydial infection | 233949.24 | 537257.90 | 129.65 | -0.24(-0.33 to -0.16) |
| Côte d'Ivoire | Gonococcal infection | 145488.97 | 309376.24 | 112.65 | -0.31(-0.38 to -0.24) |
| Côte d'Ivoire | Trichomoniasis | 586138.86 | 1477983.12 | 152.16 | 0.08(-0.01 to 0.16) |
| Côte d'Ivoire | Genital herpes | 190947.00 | 431835.22 | 126.15 | -0.04(-0.06 to -0.02) |
| Democratic People's Republic of Korea | Sexually transmitted infections excluding HIV | 2250631.66 | 3156171.16 | 40.23 | 0(0 to 0.01) |
| Democratic People's Republic of Korea | Syphilis | 19124.92 | 25620.66 | 33.96 | 0.07(0.06 to 0.08) |
| Democratic People's Republic of Korea | Chlamydial infection | 1028342.12 | 1396760.29 | 35.83 | -0.03(-0.04 to -0.02) |
| Democratic People's Republic of Korea | Gonococcal infection | 202863.96 | 242859.01 | 19.72 | -0.01(-0.05 to 0.04) |
| Democratic People's Republic of Korea | Trichomoniasis | 834583.23 | 1277509.73 | 53.07 | 0.07(0.05 to 0.08) |
| Democratic People's Republic of Korea | Genital herpes | 165717.42 | 213421.45 | 28.79 | -0.11(-0.12 to -0.1) |
| Democratic Republic of the Congo | Sexually transmitted infections excluding HIV | 3840282.55 | 9383230.44 | 144.34 | -0.05(-0.07 to -0.02) |
| Democratic Republic of the Congo | Syphilis | 393174.13 | 865114.63 | 120.03 | -0.54(-0.67 to -0.4) |
| Democratic Republic of the Congo | Chlamydial infection | 652924.23 | 1627934.63 | 149.33 | 0.03(0.01 to 0.05) |
| Democratic Republic of the Congo | Gonococcal infection | 451842.13 | 1070632.08 | 136.95 | -0.09(-0.13 to -0.06) |
| Democratic Republic of the Congo | Trichomoniasis | 1464371.82 | 3696606.09 | 152.44 | 0.02(-0.02 to 0.06) |
| Democratic Republic of the Congo | Genital herpes | 877970.24 | 2122943.00 | 141.80 | -0.02(-0.03 to -0.01) |
| Denmark | Sexually transmitted infections excluding HIV | 218213.81 | 227127.03 | 4.08 | -0.05(-0.06 to -0.04) |
| Denmark | Syphilis | 3372.57 | 3268.12 | -3.10 | -0.19(-0.23 to -0.15) |
| Denmark | Chlamydial infection | 28782.97 | 29004.04 | 0.77 | -0.09(-0.23 to 0.05) |
| Denmark | Gonococcal infection | 6545.53 | 6489.96 | -0.85 | -0.27(-0.32 to -0.22) |
| Denmark | Trichomoniasis | 137247.83 | 148638.05 | 8.30 | -0.01(-0.02 to 0.01) |
| Denmark | Genital herpes | 42264.90 | 39726.85 | -6.01 | -0.11(-0.21 to -0.01) |
| Djibouti | Sexually transmitted infections excluding HIV | 65623.67 | 197250.12 | 200.58 | -0.07(-0.09 to -0.04) |
| Djibouti | Syphilis | 1051.62 | 2695.15 | 156.29 | -0.06(-0.18 to 0.05) |
| Djibouti | Chlamydial infection | 14595.58 | 41598.71 | 185.01 | -0.1(-0.12 to -0.08) |
| Djibouti | Gonococcal infection | 6922.19 | 15571.73 | 124.95 | -0.22(-0.26 to -0.18) |
| Djibouti | Trichomoniasis | 34275.68 | 115323.91 | 236.46 | -0.06(-0.11 to -0.01) |
| Djibouti | Genital herpes | 8778.61 | 22060.61 | 151.30 | 0.05(0.03 to 0.06) |
| Dominica | Sexually transmitted infections excluding HIV | 8489.81 | 9061.13 | 6.73 | 0.01(0 to 0.03) |
| Dominica | Syphilis | 158.01 | 144.73 | -8.40 | 0(-0.04 to 0.05) |
| Dominica | Chlamydial infection | 3004.58 | 3127.68 | 4.10 | 0(-0.01 to 0) |
| Dominica | Gonococcal infection | 1003.71 | 852.88 | -15.03 | -0.21(-0.28 to -0.14) |
| Dominica | Trichomoniasis | 3135.63 | 3832.84 | 22.24 | 0.07(0.04 to 0.1) |
| Dominica | Genital herpes | 1187.89 | 1103.00 | -7.15 | 0.03(0.02 to 0.05) |
| Dominican Republic | Sexually transmitted infections excluding HIV | 853707.16 | 1463706.13 | 71.45 | 0(-0.01 to 0.02) |
| Dominican Republic | Syphilis | 13371.38 | 23254.61 | 73.91 | 0.14(0.01 to 0.27) |
| Dominican Republic | Chlamydial infection | 311030.89 | 523408.20 | 68.28 | 0(-0.01 to 0) |
| Dominican Republic | Gonococcal infection | 106720.88 | 146441.24 | 37.22 | -0.23(-0.29 to -0.18) |
| Dominican Republic | Trichomoniasis | 295642.25 | 580839.23 | 96.47 | 0.08(0.05 to 0.11) |
| Dominican Republic | Genital herpes | 126941.76 | 189762.85 | 49.49 | -0.06(-0.07 to -0.05) |
| Ecuador | Sexually transmitted infections excluding HIV | 789850.68 | 1568558.52 | 98.59 | 0(-0.02 to 0.02) |
| Ecuador | Syphilis | 22542.21 | 41557.91 | 84.36 | -0.02(-0.04 to 0) |
| Ecuador | Chlamydial infection | 190511.27 | 372264.32 | 95.40 | 0.02(-0.02 to 0.05) |
| Ecuador | Gonococcal infection | 27015.87 | 46480.54 | 72.05 | -0.13(-0.18 to -0.08) |
| Ecuador | Trichomoniasis | 362484.36 | 780058.06 | 115.20 | 0(-0.02 to 0.03) |
| Ecuador | Genital herpes | 187296.96 | 328197.69 | 75.23 | -0.02(-0.03 to -0.01) |
| Egypt | Sexually transmitted infections excluding HIV | 5115308.28 | 9488070.96 | 85.48 | -0.37(-0.43 to -0.31) |
| Egypt | Syphilis | 28625.31 | 55971.65 | 95.53 | 0.21(0.12 to 0.31) |
| Egypt | Chlamydial infection | 2067540.56 | 3644779.78 | 76.29 | -0.47(-0.56 to -0.38) |
| Egypt | Gonococcal infection | 788354.20 | 1336019.47 | 69.47 | -0.54(-0.69 to -0.4) |
| Egypt | Trichomoniasis | 1796595.25 | 3628702.09 | 101.98 | -0.29(-0.38 to -0.19) |
| Egypt | Genital herpes | 434192.95 | 822597.98 | 89.45 | -0.01(-0.02 to -0.01) |
| El Salvador | Sexually transmitted infections excluding HIV | 558724.91 | 798416.84 | 42.90 | 0.04(0.01 to 0.06) |
| El Salvador | Syphilis | 4856.74 | 6012.97 | 23.81 | -0.31(-0.36 to -0.26) |
| El Salvador | Chlamydial infection | 175659.37 | 239501.34 | 36.34 | -0.05(-0.06 to -0.04) |
| El Salvador | Gonococcal infection | 43476.26 | 50295.93 | 15.69 | -0.24(-0.28 to -0.19) |
| El Salvador | Trichomoniasis | 244744.44 | 389928.46 | 59.32 | 0.13(0.08 to 0.19) |
| El Salvador | Genital herpes | 89988.11 | 112678.14 | 25.21 | 0.01(0 to 0.03) |
| Equatorial Guinea | Sexually transmitted infections excluding HIV | 42156.40 | 167232.41 | 296.70 | -0.06(-0.12 to -0.01) |
| Equatorial Guinea | Syphilis | 4427.67 | 19615.46 | 343.02 | 0.09(-0.05 to 0.22) |
| Equatorial Guinea | Chlamydial infection | 7161.76 | 29668.23 | 314.26 | 0.03(-0.01 to 0.07) |
| Equatorial Guinea | Gonococcal infection | 4948.96 | 20732.55 | 318.93 | -0.12(-0.18 to -0.06) |
| Equatorial Guinea | Trichomoniasis | 16220.28 | 60172.33 | 270.97 | -0.02(-0.09 to 0.05) |
| Equatorial Guinea | Genital herpes | 9397.72 | 37043.83 | 294.18 | -0.31(-0.33 to -0.29) |
| Eritrea | Sexually transmitted infections excluding HIV | 392937.76 | 993839.42 | 152.93 | -0.07(-0.1 to -0.03) |
| Eritrea | Syphilis | 13133.17 | 27811.88 | 111.77 | -0.67(-0.74 to -0.6) |
| Eritrea | Chlamydial infection | 83525.54 | 212571.35 | 154.50 | -0.02(-0.03 to -0.01) |
| Eritrea | Gonococcal infection | 41932.78 | 95970.53 | 128.87 | -0.22(-0.25 to -0.19) |
| Eritrea | Trichomoniasis | 203421.79 | 529343.46 | 160.22 | -0.05(-0.12 to 0.02) |
| Eritrea | Genital herpes | 50924.48 | 128142.20 | 151.63 | 0.05(0.03 to 0.06) |
| Estonia | Sexually transmitted infections excluding HIV | 155395.77 | 121570.70 | -21.77 | -0.09(-0.11 to -0.08) |
| Estonia | Syphilis | 772.86 | 590.42 | -23.61 | -0.12(-0.2 to -0.05) |
| Estonia | Chlamydial infection | 60180.02 | 47677.08 | -20.78 | -0.01(-0.02 to 0) |
| Estonia | Gonococcal infection | 29973.94 | 17844.16 | -40.47 | -0.5(-0.59 to -0.42) |
| Estonia | Trichomoniasis | 49956.97 | 44633.65 | -10.66 | 0.09(0.07 to 0.11) |
| Estonia | Genital herpes | 14511.98 | 10825.39 | -25.40 | -0.15(-0.22 to -0.09) |
| Eswatini | Sexually transmitted infections excluding HIV | 109865.76 | 186803.38 | 70.03 | -0.06(-0.09 to -0.03) |
| Eswatini | Syphilis | 5356.21 | 5861.57 | 9.44 | -1.12(-1.32 to -0.93) |
| Eswatini | Chlamydial infection | 23094.93 | 39949.74 | 72.98 | -0.07(-0.08 to -0.06) |
| Eswatini | Gonococcal infection | 22658.12 | 34016.49 | 50.13 | -0.2(-0.25 to -0.15) |
| Eswatini | Trichomoniasis | 41535.54 | 75948.22 | 82.85 | -0.04(-0.12 to 0.03) |
| Eswatini | Genital herpes | 17220.96 | 31027.35 | 80.17 | 0.32(0.26 to 0.39) |
| Ethiopia | Sexually transmitted infections excluding HIV | 6540122.31 | 14583237.99 | 122.98 | -0.29(-0.38 to -0.2) |
| Ethiopia | Syphilis | 217575.26 | 241268.27 | 10.89 | -2.67(-2.98 to -2.36) |
| Ethiopia | Chlamydial infection | 1384482.13 | 3137679.31 | 126.63 | -0.11(-0.12 to -0.09) |
| Ethiopia | Gonococcal infection | 511329.27 | 1098382.28 | 114.81 | -0.28(-0.32 to -0.24) |
| Ethiopia | Trichomoniasis | 3679449.33 | 8362504.77 | 127.28 | -0.09(-0.13 to -0.04) |
| Ethiopia | Genital herpes | 747286.33 | 1743403.36 | 133.30 | -1.55(-2.2 to -0.9) |
| Fiji | Sexually transmitted infections excluding HIV | 110117.49 | 129185.23 | 17.32 | -0.14(-0.23 to -0.06) |
| Fiji | Syphilis | 2509.52 | 2918.63 | 16.30 | -0.05(-0.1 to 0) |
| Fiji | Chlamydial infection | 44718.18 | 44183.37 | -1.20 | -0.44(-0.7 to -0.18) |
| Fiji | Gonococcal infection | 10668.25 | 10703.00 | 0.33 | -0.28(-0.31 to -0.25) |
| Fiji | Trichomoniasis | 41219.34 | 58739.24 | 42.50 | 0.09(0.06 to 0.12) |
| Fiji | Genital herpes | 11002.20 | 12640.99 | 14.90 | 0.02(0.01 to 0.03) |
| Finland | Sexually transmitted infections excluding HIV | 208478.70 | 203739.93 | -2.27 | -0.12(-0.18 to -0.07) |
| Finland | Syphilis | 3467.08 | 3202.33 | -7.64 | -0.05(-0.06 to -0.03) |
| Finland | Chlamydial infection | 21066.01 | 20049.41 | -4.83 | -0.04(-0.18 to 0.1) |
| Finland | Gonococcal infection | 6389.89 | 5710.71 | -10.63 | -0.32(-0.34 to -0.3) |
| Finland | Trichomoniasis | 135950.97 | 142164.18 | 4.57 | 0.03(0.02 to 0.04) |
| Finland | Genital herpes | 41604.75 | 32613.31 | -21.61 | -0.65(-0.98 to -0.31) |
| France | Sexually transmitted infections excluding HIV | 2284975.92 | 2456293.28 | 7.50 | -0.06(-0.08 to -0.04) |
| France | Syphilis | 39198.52 | 37601.86 | -4.07 | -0.09(-0.11 to -0.07) |
| France | Chlamydial infection | 212834.31 | 218041.80 | 2.45 | 0.01(-0.06 to 0.08) |
| France | Gonococcal infection | 73815.34 | 72093.39 | -2.33 | -0.2(-0.24 to -0.17) |
| France | Trichomoniasis | 1559518.14 | 1728895.02 | 10.86 | -0.04(-0.06 to -0.03) |
| France | Genital herpes | 399609.61 | 399661.20 | 0.01 | -0.14(-0.19 to -0.08) |
| Gabon | Sexually transmitted infections excluding HIV | 106261.41 | 214532.00 | 101.89 | -0.22(-0.26 to -0.18) |
| Gabon | Syphilis | 10406.43 | 18592.04 | 78.66 | -0.97(-1.14 to -0.8) |
| Gabon | Chlamydial infection | 18725.60 | 38020.75 | 103.04 | -0.15(-0.17 to -0.13) |
| Gabon | Gonococcal infection | 12453.82 | 22391.45 | 79.80 | -0.32(-0.36 to -0.29) |
| Gabon | Trichomoniasis | 41339.47 | 89884.83 | 117.43 | -0.07(-0.11 to -0.04) |
| Gabon | Genital herpes | 23336.09 | 45642.94 | 95.59 | -0.23(-0.37 to -0.09) |
| Gambia | Sexually transmitted infections excluding HIV | 90180.45 | 223909.42 | 148.29 | -0.03(-0.06 to 0.01) |
| Gambia | Syphilis | 3485.98 | 8296.27 | 137.99 | -0.4(-0.53 to -0.27) |
| Gambia | Chlamydial infection | 11274.43 | 27352.45 | 142.61 | 0.07(0.01 to 0.13) |
| Gambia | Gonococcal infection | 12422.58 | 29546.22 | 137.84 | -0.21(-0.24 to -0.18) |
| Gambia | Trichomoniasis | 49333.71 | 124669.15 | 152.71 | -0.03(-0.09 to 0.03) |
| Gambia | Genital herpes | 13663.75 | 34045.33 | 149.17 | 0.16(0.12 to 0.21) |
| Georgia | Sexually transmitted infections excluding HIV | 692531.95 | 438759.96 | -36.64 | -0.08(-0.09 to -0.06) |
| Georgia | Syphilis | 3215.50 | 1893.12 | -41.13 | -0.62(-0.82 to -0.43) |
| Georgia | Chlamydial infection | 296381.82 | 185425.31 | -37.44 | -0.08(-0.09 to -0.06) |
| Georgia | Gonococcal infection | 128429.88 | 66669.71 | -48.09 | -0.21(-0.28 to -0.14) |
| Georgia | Trichomoniasis | 225410.15 | 160580.73 | -28.76 | 0(-0.03 to 0.02) |
| Georgia | Genital herpes | 39094.60 | 24191.10 | -38.12 | -0.03(-0.04 to -0.02) |
| Germany | Sexually transmitted infections excluding HIV | 3582267.81 | 3444660.91 | -3.84 | -0.07(-0.18 to 0.04) |
| Germany | Syphilis | 54755.44 | 48707.24 | -11.05 | -0.12(-0.15 to -0.09) |
| Germany | Chlamydial infection | 461403.43 | 434485.65 | -5.83 | 0.15(-0.02 to 0.32) |
| Germany | Gonococcal infection | 105430.98 | 94246.99 | -10.61 | -0.23(-0.24 to -0.21) |
| Germany | Trichomoniasis | 2167167.08 | 2261404.78 | 4.35 | 0.01(0 to 0.01) |
| Germany | Genital herpes | 793510.88 | 605816.26 | -23.65 | -0.4(-0.76 to -0.03) |
| Ghana | Sexually transmitted infections excluding HIV | 1817753.99 | 4407116.55 | 142.45 | 0.05(0.01 to 0.09) |
| Ghana | Syphilis | 58583.07 | 133750.18 | 128.31 | -0.29(-0.35 to -0.24) |
| Ghana | Chlamydial infection | 436820.59 | 1058250.41 | 142.26 | 0.27(0.16 to 0.38) |
| Ghana | Gonococcal infection | 301505.05 | 660027.20 | 118.91 | 0.02(-0.11 to 0.14) |
| Ghana | Trichomoniasis | 787688.17 | 2002494.74 | 154.22 | -0.03(-0.08 to 0.02) |
| Ghana | Genital herpes | 233157.12 | 552594.02 | 137.00 | 0.03(0.02 to 0.04) |
| Greece | Sexually transmitted infections excluding HIV | 425647.19 | 429013.59 | 0.79 | 0.13(0 to 0.26) |
| Greece | Syphilis | 4476.69 | 5923.33 | 32.31 | 3.38(2.5 to 4.26) |
| Greece | Chlamydial infection | 54549.52 | 51858.62 | -4.93 | 0.21(-0.04 to 0.47) |
| Greece | Gonococcal infection | 12868.85 | 10683.05 | -16.99 | -0.12(-0.17 to -0.07) |
| Greece | Trichomoniasis | 292695.85 | 306811.68 | 4.82 | 0.09(-0.08 to 0.26) |
| Greece | Genital herpes | 61056.29 | 53736.91 | -11.99 | 0.01(-0.12 to 0.13) |
| Greenland | Sexually transmitted infections excluding HIV | 3593.12 | 3397.34 | -5.45 | 0(-0.01 to 0.01) |
| Greenland | Syphilis | 40.63 | 33.21 | -18.25 | -0.17(-0.18 to -0.15) |
| Greenland | Chlamydial infection | 355.74 | 298.26 | -16.16 | -0.11(-0.13 to -0.1) |
| Greenland | Gonococcal infection | 202.68 | 164.13 | -19.02 | -0.3(-0.35 to -0.25) |
| Greenland | Trichomoniasis | 2422.79 | 2417.64 | -0.21 | 0.03(0.01 to 0.04) |
| Greenland | Genital herpes | 571.28 | 484.10 | -15.26 | 0.04(0.04 to 0.05) |
| Grenada | Sexually transmitted infections excluding HIV | 9455.82 | 14252.91 | 50.73 | 0.01(0 to 0.02) |
| Grenada | Syphilis | 162.61 | 232.47 | 42.96 | 0.32(0.23 to 0.41) |
| Grenada | Chlamydial infection | 3395.45 | 5003.27 | 47.35 | -0.02(-0.03 to -0.01) |
| Grenada | Gonococcal infection | 1134.83 | 1406.72 | 23.96 | -0.2(-0.25 to -0.14) |
| Grenada | Trichomoniasis | 3409.23 | 5852.65 | 71.67 | 0.09(0.08 to 0.11) |
| Grenada | Genital herpes | 1353.70 | 1757.80 | 29.85 | -0.02(-0.03 to 0) |
| Guam | Sexually transmitted infections excluding HIV | 17454.10 | 20371.78 | 16.72 | 0.06(0.02 to 0.1) |
| Guam | Syphilis | 493.40 | 513.62 | 4.10 | -0.02(-0.05 to 0.02) |
| Guam | Chlamydial infection | 4179.11 | 4608.21 | 10.27 | 0.08(0.01 to 0.15) |
| Guam | Gonococcal infection | 1574.22 | 1562.49 | -0.75 | -0.28(-0.34 to -0.21) |
| Guam | Trichomoniasis | 9067.94 | 11488.78 | 26.70 | 0.11(0.07 to 0.15) |
| Guam | Genital herpes | 2139.43 | 2198.69 | 2.77 | 0.02(0.01 to 0.03) |
| Guatemala | Sexually transmitted infections excluding HIV | 813577.24 | 2252698.83 | 176.89 | 0(-0.03 to 0.02) |
| Guatemala | Syphilis | 7655.33 | 20334.65 | 165.63 | -0.28(-0.33 to -0.22) |
| Guatemala | Chlamydial infection | 247393.73 | 666416.07 | 169.37 | -0.12(-0.14 to -0.1) |
| Guatemala | Gonococcal infection | 89183.85 | 208786.48 | 134.11 | -0.48(-0.54 to -0.43) |
| Guatemala | Trichomoniasis | 344493.20 | 1024237.53 | 197.32 | 0.15(0.11 to 0.2) |
| Guatemala | Genital herpes | 124851.13 | 332924.10 | 166.66 | -0.01(-0.03 to 0.01) |
| Guinea | Sexually transmitted infections excluding HIV | 603293.41 | 1266266.37 | 109.89 | -0.02(-0.05 to 0) |
| Guinea | Syphilis | 24146.82 | 48111.63 | 99.25 | -0.46(-0.61 to -0.3) |
| Guinea | Chlamydial infection | 96259.43 | 204486.76 | 112.43 | -0.02(-0.04 to -0.01) |
| Guinea | Gonococcal infection | 74028.97 | 154700.11 | 108.97 | -0.21(-0.25 to -0.18) |
| Guinea | Trichomoniasis | 319763.36 | 658725.82 | 106.00 | 0.01(-0.04 to 0.06) |
| Guinea | Genital herpes | 89094.84 | 200242.05 | 124.75 | 0.08(0.06 to 0.09) |
| Guinea-Bissau | Sexually transmitted infections excluding HIV | 99294.36 | 208466.64 | 109.95 | -0.09(-0.11 to -0.07) |
| Guinea-Bissau | Syphilis | 5610.50 | 9319.94 | 66.12 | -1.13(-1.54 to -0.73) |
| Guinea-Bissau | Chlamydial infection | 15915.67 | 34171.92 | 114.71 | -0.03(-0.05 to -0.02) |
| Guinea-Bissau | Gonococcal infection | 13146.86 | 25543.39 | 94.29 | -0.25(-0.28 to -0.22) |
| Guinea-Bissau | Trichomoniasis | 49334.62 | 106898.46 | 116.68 | -0.02(-0.07 to 0.03) |
| Guinea-Bissau | Genital herpes | 15286.71 | 32532.94 | 112.82 | 0.03(0.02 to 0.04) |
| Guyana | Sexually transmitted infections excluding HIV | 94950.59 | 104160.25 | 9.70 | 0(-0.01 to 0.01) |
| Guyana | Syphilis | 1287.87 | 1300.39 | 0.97 | -0.08(-0.12 to -0.04) |
| Guyana | Chlamydial infection | 35122.62 | 37616.88 | 7.10 | -0.01(-0.01 to 0) |
| Guyana | Gonococcal infection | 11774.20 | 11124.04 | -5.52 | -0.25(-0.34 to -0.17) |
| Guyana | Trichomoniasis | 32856.95 | 40326.38 | 22.73 | 0.07(0.04 to 0.1) |
| Guyana | Genital herpes | 13908.95 | 13792.56 | -0.84 | 0.02(0 to 0.03) |
| Haiti | Sexually transmitted infections excluding HIV | 661561.32 | 1510964.57 | 128.39 | 0(-0.02 to 0.01) |
| Haiti | Syphilis | 14044.75 | 34649.09 | 146.70 | 0.39(0.16 to 0.62) |
| Haiti | Chlamydial infection | 222499.29 | 508120.52 | 128.37 | 0.03(0.01 to 0.05) |
| Haiti | Gonococcal infection | 83983.36 | 170018.30 | 102.44 | -0.36(-0.47 to -0.25) |
| Haiti | Trichomoniasis | 251148.11 | 602314.95 | 139.82 | 0.05(0.02 to 0.08) |
| Haiti | Genital herpes | 89885.81 | 195861.71 | 117.90 | -0.04(-0.05 to -0.02) |
| Honduras | Sexually transmitted infections excluding HIV | 473395.11 | 1243511.36 | 162.68 | 0.07(0.01 to 0.14) |
| Honduras | Syphilis | 4060.69 | 9392.75 | 131.31 | -0.48(-0.57 to -0.39) |
| Honduras | Chlamydial infection | 145177.36 | 372445.21 | 156.54 | -0.07(-0.08 to -0.05) |
| Honduras | Gonococcal infection | 38071.83 | 88905.78 | 133.52 | -0.17(-0.23 to -0.11) |
| Honduras | Trichomoniasis | 195513.21 | 553690.52 | 183.20 | 0.11(0.06 to 0.17) |
| Honduras | Genital herpes | 90572.02 | 219077.09 | 141.88 | 0.32(-0.08 to 0.72) |
| Hungary | Sexually transmitted infections excluding HIV | 947410.02 | 862776.22 | -8.93 | 0(-0.02 to 0.02) |
| Hungary | Syphilis | 4188.45 | 3724.88 | -11.07 | 0.02(0.01 to 0.02) |
| Hungary | Chlamydial infection | 338775.14 | 303826.27 | -10.32 | 0(-0.01 to 0.01) |
| Hungary | Gonococcal infection | 183310.66 | 137461.96 | -25.01 | -0.24(-0.27 to -0.2) |
| Hungary | Trichomoniasis | 377470.68 | 378811.73 | 0.36 | 0.05(0.04 to 0.07) |
| Hungary | Genital herpes | 43665.08 | 38951.38 | -10.80 | 0.76(0.44 to 1.07) |
| Iceland | Sexually transmitted infections excluding HIV | 9513.78 | 13157.70 | 38.30 | 0.01(-0.05 to 0.08) |
| Iceland | Syphilis | 182.22 | 224.32 | 23.11 | -0.08(-0.1 to -0.06) |
| Iceland | Chlamydial infection | 906.34 | 1195.41 | 31.89 | 0.01(0 to 0.03) |
| Iceland | Gonococcal infection | 336.02 | 398.37 | 18.56 | -0.28(-0.32 to -0.25) |
| Iceland | Trichomoniasis | 6167.60 | 8934.32 | 44.86 | 0.02(0 to 0.05) |
| Iceland | Genital herpes | 1921.60 | 2405.27 | 25.17 | 0.03(-0.26 to 0.31) |
| India | Sexually transmitted infections excluding HIV | 54470294.44 | 99910877.62 | 83.42 | -0.1(-0.13 to -0.07) |
| India | Syphilis | 1792136.43 | 2729026.77 | 52.28 | -0.86(-1.17 to -0.56) |
| India | Chlamydial infection | 12896025.16 | 23788057.39 | 84.46 | -0.28(-0.5 to -0.06) |
| India | Gonococcal infection | 12619369.72 | 20523836.99 | 62.64 | 0.16(-0.06 to 0.38) |
| India | Trichomoniasis | 22294906.91 | 43092523.98 | 93.28 | -0.18(-0.22 to -0.13) |
| India | Genital herpes | 4867856.21 | 9777432.49 | 100.86 | 0.33(0.24 to 0.43) |
| Indonesia | Sexually transmitted infections excluding HIV | 19960258.39 | 32611321.98 | 63.38 | -0.04(-0.04 to -0.03) |
| Indonesia | Syphilis | 169850.75 | 242157.45 | 42.57 | -0.2(-0.22 to -0.18) |
| Indonesia | Chlamydial infection | 7476287.72 | 11997561.09 | 60.47 | -0.05(-0.07 to -0.04) |
| Indonesia | Gonococcal infection | 2922272.73 | 4048739.52 | 38.55 | -0.09(-0.13 to -0.04) |
| Indonesia | Trichomoniasis | 7397041.80 | 13385729.43 | 80.96 | -0.01(-0.03 to 0.01) |
| Indonesia | Genital herpes | 1994805.39 | 2937134.49 | 47.24 | -0.01(-0.02 to 0) |
| Iran (Islamic Republic of) | Sexually transmitted infections excluding HIV | 4490007.13 | 8897603.57 | 98.16 | 0.11(-0.08 to 0.3) |
| Iran (Islamic Republic of) | Syphilis | 22787.58 | 44319.24 | 94.49 | 0.21(0.07 to 0.36) |
| Iran (Islamic Republic of) | Chlamydial infection | 1613520.32 | 3440463.82 | 113.23 | 0.49(0.12 to 0.86) |
| Iran (Islamic Republic of) | Gonococcal infection | 722468.88 | 1014058.88 | 40.36 | 0.02(-0.09 to 0.12) |
| Iran (Islamic Republic of) | Trichomoniasis | 1605945.24 | 3487926.42 | 117.19 | -0.2(-0.37 to -0.03) |
| Iran (Islamic Republic of) | Genital herpes | 525285.10 | 910835.20 | 73.40 | -0.02(-0.04 to 0) |
| Iraq | Sexually transmitted infections excluding HIV | 1484584.19 | 3950026.93 | 166.07 | -0.47(-0.56 to -0.38) |
| Iraq | Syphilis | 12943.57 | 38295.91 | 195.87 | -0.06(-0.08 to -0.04) |
| Iraq | Chlamydial infection | 597209.29 | 1484738.03 | 148.61 | -0.61(-0.77 to -0.45) |
| Iraq | Gonococcal infection | 196093.34 | 506359.44 | 158.22 | -0.34(-0.38 to -0.3) |
| Iraq | Trichomoniasis | 550064.41 | 1553308.57 | 182.39 | -0.47(-0.77 to -0.17) |
| Iraq | Genital herpes | 128273.58 | 367324.97 | 186.36 | 0(-0.01 to 0.01) |
| Ireland | Sexually transmitted infections excluding HIV | 127337.25 | 188395.50 | 47.95 | 0.01(-0.02 to 0.04) |
| Ireland | Syphilis | 2341.69 | 3000.62 | 28.14 | -0.09(-0.11 to -0.06) |
| Ireland | Chlamydial infection | 14252.16 | 19859.31 | 39.34 | 0.06(-0.13 to 0.25) |
| Ireland | Gonococcal infection | 4561.09 | 5490.82 | 20.38 | -0.25(-0.3 to -0.19) |
| Ireland | Trichomoniasis | 81523.79 | 128088.89 | 57.12 | 0.01(-0.01 to 0.03) |
| Ireland | Genital herpes | 24658.52 | 31955.85 | 29.59 | 0.02(0.01 to 0.03) |
| Israel | Sexually transmitted infections excluding HIV | 173237.86 | 327485.46 | 89.04 | -0.08(-0.11 to -0.05) |
| Israel | Syphilis | 3158.97 | 5687.28 | 80.04 | -0.02(-0.04 to -0.01) |
| Israel | Chlamydial infection | 24633.89 | 40322.70 | 63.69 | -0.47(-0.61 to -0.33) |
| Israel | Gonococcal infection | 6294.41 | 10743.86 | 70.69 | -0.23(-0.27 to -0.19) |
| Israel | Trichomoniasis | 106365.84 | 213633.42 | 100.85 | 0.05(0.03 to 0.06) |
| Israel | Genital herpes | 32784.75 | 57098.19 | 74.16 | -0.2(-0.24 to -0.16) |
| Italy | Sexually transmitted infections excluding HIV | 2386583.28 | 2497243.45 | 4.64 | 0.02(0.01 to 0.04) |
| Italy | Syphilis | 42792.62 | 36037.83 | -15.78 | -0.21(-0.24 to -0.18) |
| Italy | Chlamydial infection | 256860.98 | 247367.31 | -3.70 | 0(-0.05 to 0.05) |
| Italy | Gonococcal infection | 83139.48 | 73947.63 | -11.06 | -0.07(-0.1 to -0.05) |
| Italy | Trichomoniasis | 1608782.52 | 1780796.53 | 10.69 | 0.03(0.02 to 0.05) |
| Italy | Genital herpes | 395007.68 | 359094.15 | -9.09 | 0.04(0.03 to 0.05) |
| Jamaica | Sexually transmitted infections excluding HIV | 285663.45 | 398167.17 | 39.38 | -0.05(-0.07 to -0.03) |
| Jamaica | Syphilis | 4408.36 | 5559.25 | 26.11 | 0.49(0.31 to 0.67) |
| Jamaica | Chlamydial infection | 99849.50 | 139761.63 | 39.97 | -0.01(-0.02 to -0.01) |
| Jamaica | Gonococcal infection | 31697.60 | 37657.80 | 18.80 | -0.1(-0.14 to -0.05) |
| Jamaica | Trichomoniasis | 98302.05 | 158836.54 | 61.58 | 0.08(0.05 to 0.11) |
| Jamaica | Genital herpes | 51405.95 | 56351.95 | 9.62 | -0.49(-0.57 to -0.4) |
| Japan | Sexually transmitted infections excluding HIV | 7848097.60 | 7266404.58 | -7.41 | 0.04(0.02 to 0.05) |
| Japan | Syphilis | 105989.89 | 86800.61 | -18.10 | 0.03(-0.02 to 0.08) |
| Japan | Chlamydial infection | 1335772.63 | 1134688.93 | -15.05 | -0.01(-0.02 to 0) |
| Japan | Gonococcal infection | 892992.53 | 709165.84 | -20.59 | 0.04(0 to 0.07) |
| Japan | Trichomoniasis | 4771613.57 | 4702299.12 | -1.45 | 0.05(0.03 to 0.07) |
| Japan | Genital herpes | 741728.97 | 633450.07 | -14.60 | 0.07(0.01 to 0.13) |
| Jordan | Sexually transmitted infections excluding HIV | 200993.43 | 755833.49 | 276.05 | -0.02(-0.07 to 0.02) |
| Jordan | Syphilis | 3654.12 | 12870.23 | 252.21 | -0.02(-0.04 to 0) |
| Jordan | Chlamydial infection | 42354.51 | 155792.81 | 267.83 | 0.02(-0.08 to 0.12) |
| Jordan | Gonococcal infection | 42968.33 | 122702.51 | 185.56 | -0.23(-0.25 to -0.21) |
| Jordan | Trichomoniasis | 77932.02 | 348912.44 | 347.71 | 0.04(-0.02 to 0.1) |
| Jordan | Genital herpes | 34084.45 | 115555.50 | 239.03 | -0.08(-0.1 to -0.05) |
| Kazakhstan | Sexually transmitted infections excluding HIV | 2035359.83 | 2350983.23 | 15.51 | -0.08(-0.1 to -0.05) |
| Kazakhstan | Syphilis | 8647.38 | 9208.13 | 6.48 | -0.29(-0.38 to -0.21) |
| Kazakhstan | Chlamydial infection | 883207.07 | 1033323.25 | 17.00 | -0.02(-0.03 to -0.01) |
| Kazakhstan | Gonococcal infection | 415024.83 | 385949.62 | -7.01 | -0.41(-0.48 to -0.34) |
| Kazakhstan | Trichomoniasis | 611663.64 | 789204.42 | 29.03 | 0.04(0.01 to 0.07) |
| Kazakhstan | Genital herpes | 116816.91 | 133297.80 | 14.11 | 0.01(0 to 0.02) |
| Kenya | Sexually transmitted infections excluding HIV | 2843966.41 | 7724018.29 | 171.59 | -0.14(-0.21 to -0.07) |
| Kenya | Syphilis | 106894.43 | 191609.76 | 79.25 | -1.6(-1.97 to -1.23) |
| Kenya | Chlamydial infection | 540978.71 | 1456085.76 | 169.16 | 0.11(0.03 to 0.19) |
| Kenya | Gonococcal infection | 298639.44 | 708622.24 | 137.28 | 0.52(0.24 to 0.8) |
| Kenya | Trichomoniasis | 1419446.28 | 4206014.48 | 196.31 | -0.13(-0.23 to -0.03) |
| Kenya | Genital herpes | 478007.54 | 1161686.06 | 143.03 | -0.63(-0.87 to -0.4) |
| Kiribati | Sexually transmitted infections excluding HIV | 8614.28 | 14874.57 | 72.67 | 0(-0.04 to 0.04) |
| Kiribati | Syphilis | 274.22 | 459.92 | 67.72 | 0.11(0.06 to 0.17) |
| Kiribati | Chlamydial infection | 2548.96 | 4238.10 | 66.27 | -0.1(-0.22 to 0.02) |
| Kiribati | Gonococcal infection | 633.80 | 1011.88 | 59.65 | -0.19(-0.3 to -0.09) |
| Kiribati | Trichomoniasis | 4125.88 | 7479.83 | 81.29 | 0.07(0.04 to 0.09) |
| Kiribati | Genital herpes | 1031.42 | 1684.84 | 63.35 | 0.02(0 to 0.04) |
| Kuwait | Sexually transmitted infections excluding HIV | 172131.02 | 472846.91 | 174.70 | -0.22(-0.28 to -0.16) |
| Kuwait | Syphilis | 1971.30 | 4874.11 | 147.25 | -0.29(-0.37 to -0.2) |
| Kuwait | Chlamydial infection | 65303.86 | 171113.00 | 162.03 | -0.27(-0.37 to -0.18) |
| Kuwait | Gonococcal infection | 25661.77 | 51224.42 | 99.61 | -0.24(-0.49 to 0) |
| Kuwait | Trichomoniasis | 63482.47 | 202792.73 | 219.45 | -0.25(-0.28 to -0.22) |
| Kuwait | Genital herpes | 15711.61 | 42842.66 | 172.68 | 0.12(0.07 to 0.17) |
| Kyrgyzstan | Sexually transmitted infections excluding HIV | 505896.40 | 823695.07 | 62.82 | -0.06(-0.09 to -0.03) |
| Kyrgyzstan | Syphilis | 1996.56 | 2901.44 | 45.32 | -0.56(-0.69 to -0.43) |
| Kyrgyzstan | Chlamydial infection | 219355.84 | 361440.47 | 64.77 | -0.02(-0.02 to -0.01) |
| Kyrgyzstan | Gonococcal infection | 111143.68 | 157620.44 | 41.82 | -0.3(-0.41 to -0.2) |
| Kyrgyzstan | Trichomoniasis | 143964.59 | 254521.19 | 76.79 | 0.03(0 to 0.06) |
| Kyrgyzstan | Genital herpes | 29435.73 | 47211.53 | 60.39 | 0(-0.01 to 0.01) |
| Lao People's Democratic Republic | Sexually transmitted infections excluding HIV | 394347.73 | 850827.41 | 115.76 | -0.04(-0.05 to -0.02) |
| Lao People's Democratic Republic | Syphilis | 2846.19 | 6100.47 | 114.34 | 0.13(0.12 to 0.15) |
| Lao People's Democratic Republic | Chlamydial infection | 158613.94 | 345605.77 | 117.89 | -0.02(-0.04 to -0.01) |
| Lao People's Democratic Republic | Gonococcal infection | 54687.77 | 105178.23 | 92.32 | -0.23(-0.31 to -0.15) |
| Lao People's Democratic Republic | Trichomoniasis | 133325.32 | 302690.67 | 127.03 | 0.01(-0.01 to 0.03) |
| Lao People's Democratic Republic | Genital herpes | 44874.51 | 91252.27 | 103.35 | -0.03(-0.04 to -0.02) |
| Latvia | Sexually transmitted infections excluding HIV | 264554.77 | 174622.35 | -33.99 | -0.11(-0.13 to -0.09) |
| Latvia | Syphilis | 1358.53 | 837.64 | -38.34 | -0.36(-0.44 to -0.29) |
| Latvia | Chlamydial infection | 103125.56 | 67950.49 | -34.11 | -0.01(-0.02 to 0) |
| Latvia | Gonococcal infection | 50230.17 | 26007.31 | -48.22 | -0.62(-0.71 to -0.53) |
| Latvia | Trichomoniasis | 85245.96 | 64511.14 | -24.32 | 0.07(0.04 to 0.09) |
| Latvia | Genital herpes | 24594.55 | 15315.77 | -37.73 | -0.01(-0.01 to -0.01) |
| Lebanon | Sexually transmitted infections excluding HIV | 211327.84 | 393198.49 | 86.06 | -0.11(-0.24 to 0.02) |
| Lebanon | Syphilis | 2550.08 | 4619.47 | 81.15 | 0.01(-0.02 to 0.03) |
| Lebanon | Chlamydial infection | 66551.23 | 128099.58 | 92.48 | -0.03(-0.1 to 0.03) |
| Lebanon | Gonococcal infection | 33785.21 | 49451.20 | 46.37 | -0.17(-0.2 to -0.14) |
| Lebanon | Trichomoniasis | 83701.50 | 168670.61 | 101.51 | -0.19(-0.49 to 0.11) |
| Lebanon | Genital herpes | 24739.81 | 42357.63 | 71.21 | -0.02(-0.04 to 0) |
| Lesotho | Sexually transmitted infections excluding HIV | 254466.60 | 353271.03 | 38.83 | -0.03(-0.05 to 0) |
| Lesotho | Syphilis | 8553.85 | 9054.12 | 5.85 | -0.63(-0.82 to -0.44) |
| Lesotho | Chlamydial infection | 54441.67 | 76690.67 | 40.87 | -0.05(-0.06 to -0.05) |
| Lesotho | Gonococcal infection | 52367.11 | 65679.89 | 25.42 | -0.13(-0.18 to -0.09) |
| Lesotho | Trichomoniasis | 100017.42 | 144776.90 | 44.75 | -0.02(-0.08 to 0.04) |
| Lesotho | Genital herpes | 39086.55 | 57069.45 | 46.01 | 0.26(0.21 to 0.3) |
| Liberia | Sexually transmitted infections excluding HIV | 191726.59 | 555541.53 | 189.76 | -0.05(-0.09 to -0.02) |
| Liberia | Syphilis | 10912.16 | 34098.10 | 212.48 | 0.12(-0.03 to 0.26) |
| Liberia | Chlamydial infection | 29091.40 | 86762.11 | 198.24 | -0.05(-0.06 to -0.03) |
| Liberia | Gonococcal infection | 22516.55 | 63392.42 | 181.54 | -0.25(-0.28 to -0.22) |
| Liberia | Trichomoniasis | 101439.27 | 290537.86 | 186.42 | -0.04(-0.09 to 0.02) |
| Liberia | Genital herpes | 27767.22 | 80751.04 | 190.81 | -0.05(-0.07 to -0.04) |
| Libya | Sexually transmitted infections excluding HIV | 265076.53 | 583961.26 | 120.30 | -0.11(-0.22 to 0) |
| Libya | Syphilis | 3367.05 | 6829.10 | 102.82 | -0.09(-0.12 to -0.06) |
| Libya | Chlamydial infection | 103958.61 | 231258.30 | 122.45 | -0.09(-0.13 to -0.06) |
| Libya | Gonococcal infection | 47612.46 | 77876.46 | 63.56 | -0.17(-0.19 to -0.15) |
| Libya | Trichomoniasis | 78795.04 | 205304.82 | 160.56 | -0.15(-0.45 to 0.14) |
| Libya | Genital herpes | 31343.37 | 62692.57 | 100.02 | 0.01(0 to 0.03) |
| Lithuania | Sexually transmitted infections excluding HIV | 368325.94 | 258600.52 | -29.79 | -0.03(-0.05 to -0.02) |
| Lithuania | Syphilis | 1765.35 | 1222.26 | -30.76 | -0.14(-0.17 to -0.11) |
| Lithuania | Chlamydial infection | 144021.65 | 98991.05 | -31.27 | -0.01(-0.02 to 0) |
| Lithuania | Gonococcal infection | 72169.50 | 42003.44 | -41.80 | -0.23(-0.3 to -0.16) |
| Lithuania | Trichomoniasis | 115039.78 | 93464.91 | -18.75 | 0.05(0.03 to 0.08) |
| Lithuania | Genital herpes | 35329.66 | 22918.87 | -35.13 | -0.01(-0.01 to 0) |
| Luxembourg | Sexually transmitted infections excluding HIV | 15164.67 | 24831.78 | 63.75 | 0(-0.01 to 0.01) |
| Luxembourg | Syphilis | 286.39 | 417.34 | 45.73 | -0.23(-0.27 to -0.19) |
| Luxembourg | Chlamydial infection | 1088.70 | 1731.60 | 59.05 | 0.01(-0.03 to 0.06) |
| Luxembourg | Gonococcal infection | 517.19 | 767.16 | 48.33 | -0.23(-0.24 to -0.21) |
| Luxembourg | Trichomoniasis | 10515.13 | 17685.64 | 68.19 | 0.02(0.01 to 0.04) |
| Luxembourg | Genital herpes | 2757.26 | 4230.03 | 53.41 | -0.01(-0.02 to 0) |
| Madagascar | Sexually transmitted infections excluding HIV | 1619315.88 | 3996530.95 | 146.80 | -0.09(-0.13 to -0.05) |
| Madagascar | Syphilis | 90286.01 | 194731.31 | 115.68 | -0.88(-1.07 to -0.69) |
| Madagascar | Chlamydial infection | 323515.30 | 808259.53 | 149.84 | -0.02(-0.03 to -0.01) |
| Madagascar | Gonococcal infection | 157473.10 | 369229.07 | 134.47 | -0.12(-0.15 to -0.08) |
| Madagascar | Trichomoniasis | 808587.81 | 2048325.87 | 153.32 | -0.05(-0.11 to 0) |
| Madagascar | Genital herpes | 239453.66 | 575985.17 | 140.54 | -0.02(-0.03 to -0.01) |
| Malawi | Sexually transmitted infections excluding HIV | 1304546.17 | 2636800.31 | 102.12 | -0.15(-0.19 to -0.11) |
| Malawi | Syphilis | 57161.87 | 105884.43 | 85.24 | -0.82(-1.07 to -0.57) |
| Malawi | Chlamydial infection | 284616.02 | 564672.36 | 98.40 | -0.11(-0.15 to -0.08) |
| Malawi | Gonococcal infection | 161414.99 | 336299.39 | 108.34 | -0.29(-0.75 to 0.16) |
| Malawi | Trichomoniasis | 559271.10 | 1151948.06 | 105.97 | 0.09(-0.11 to 0.28) |
| Malawi | Genital herpes | 242082.19 | 477996.07 | 97.45 | -0.67(-0.94 to -0.4) |
| Malaysia | Sexually transmitted infections excluding HIV | 1884022.65 | 3882681.48 | 106.08 | -0.01(-0.02 to 0) |
| Malaysia | Syphilis | 12728.55 | 24878.94 | 95.46 | -0.07(-0.12 to -0.01) |
| Malaysia | Chlamydial infection | 759543.90 | 1538308.89 | 102.53 | 0(-0.02 to 0.01) |
| Malaysia | Gonococcal infection | 233919.57 | 441530.96 | 88.75 | -0.1(-0.16 to -0.04) |
| Malaysia | Trichomoniasis | 664925.03 | 1475305.78 | 121.88 | 0.01(-0.01 to 0.03) |
| Malaysia | Genital herpes | 212905.59 | 402656.92 | 89.12 | -0.02(-0.02 to -0.01) |
| Maldives | Sexually transmitted infections excluding HIV | 19413.92 | 77224.09 | 297.78 | 0.13(0.07 to 0.18) |
| Maldives | Syphilis | 142.30 | 582.07 | 309.04 | 0.46(0.29 to 0.64) |
| Maldives | Chlamydial infection | 7646.68 | 32014.53 | 318.67 | 0.19(0.12 to 0.26) |
| Maldives | Gonococcal infection | 2883.47 | 8564.39 | 197.02 | 0.11(-0.02 to 0.24) |
| Maldives | Trichomoniasis | 6410.06 | 28970.36 | 351.95 | 0.13(0.08 to 0.17) |
| Maldives | Genital herpes | 2331.40 | 7092.74 | 204.23 | -0.12(-0.14 to -0.1) |
| Mali | Sexually transmitted infections excluding HIV | 959661.22 | 2368610.61 | 146.82 | -0.3(-0.38 to -0.22) |
| Mali | Syphilis | 36804.66 | 101089.31 | 174.66 | 0.26(0.18 to 0.33) |
| Mali | Chlamydial infection | 148272.72 | 377678.79 | 154.72 | -0.25(-0.32 to -0.18) |
| Mali | Gonococcal infection | 96215.30 | 248181.54 | 157.94 | -0.25(-0.28 to -0.21) |
| Mali | Trichomoniasis | 518707.64 | 1265017.19 | 143.88 | -0.29(-0.39 to -0.19) |
| Mali | Genital herpes | 159660.90 | 376643.78 | 135.90 | -0.59(-0.7 to -0.48) |
| Malta | Sexually transmitted infections excluding HIV | 14228.92 | 16902.07 | 18.79 | 0.03(0.03 to 0.04) |
| Malta | Syphilis | 246.63 | 263.04 | 6.65 | -0.01(-0.02 to 0.01) |
| Malta | Chlamydial infection | 1358.33 | 1502.09 | 10.58 | 0.02(0 to 0.04) |
| Malta | Gonococcal infection | 484.04 | 485.18 | 0.23 | -0.19(-0.21 to -0.18) |
| Malta | Trichomoniasis | 9540.91 | 11913.95 | 24.87 | 0.06(0.05 to 0.07) |
| Malta | Genital herpes | 2599.02 | 2737.82 | 5.34 | -0.02(-0.02 to -0.01) |
| Marshall Islands | Sexually transmitted infections excluding HIV | 3623.34 | 5941.12 | 63.97 | -0.07(-0.15 to 0.01) |
| Marshall Islands | Syphilis | 124.26 | 189.39 | 52.41 | 0.42(-0.08 to 0.93) |
| Marshall Islands | Chlamydial infection | 331.10 | 512.60 | 54.82 | -1.47(-2.68 to -0.25) |
| Marshall Islands | Gonococcal infection | 495.14 | 633.72 | 27.99 | -0.2(-0.25 to -0.16) |
| Marshall Islands | Trichomoniasis | 2093.64 | 3781.76 | 80.63 | 0.05(0.02 to 0.08) |
| Marshall Islands | Genital herpes | 579.19 | 823.66 | 42.21 | 0.02(0 to 0.03) |
| Mauritania | Sexually transmitted infections excluding HIV | 207846.19 | 432766.73 | 108.21 | -0.09(-0.11 to -0.06) |
| Mauritania | Syphilis | 9875.37 | 18886.95 | 91.25 | -0.29(-0.37 to -0.21) |
| Mauritania | Chlamydial infection | 33079.01 | 68387.69 | 106.74 | -0.07(-0.08 to -0.06) |
| Mauritania | Gonococcal infection | 26637.73 | 51561.85 | 93.57 | -0.35(-0.38 to -0.32) |
| Mauritania | Trichomoniasis | 107343.60 | 228390.77 | 112.77 | -0.06(-0.1 to -0.02) |
| Mauritania | Genital herpes | 30910.50 | 65539.47 | 112.03 | 0.03(0.02 to 0.04) |
| Mauritius | Sexually transmitted infections excluding HIV | 130716.34 | 153657.54 | 17.55 | -0.04(-0.05 to -0.03) |
| Mauritius | Syphilis | 1197.98 | 1356.53 | 13.24 | 0.41(0.31 to 0.5) |
| Mauritius | Chlamydial infection | 52686.85 | 58040.75 | 10.16 | -0.04(-0.06 to -0.02) |
| Mauritius | Gonococcal infection | 15548.39 | 14786.02 | -4.90 | -0.3(-0.34 to -0.26) |
| Mauritius | Trichomoniasis | 46930.68 | 64717.78 | 37.90 | 0.02(0 to 0.05) |
| Mauritius | Genital herpes | 14352.45 | 14756.45 | 2.81 | 0.01(0 to 0.01) |
| Mexico | Sexually transmitted infections excluding HIV | 9833811.95 | 17504353.77 | 78.00 | 0.17(0.12 to 0.22) |
| Mexico | Syphilis | 70796.30 | 94665.25 | 33.71 | -0.83(-1.02 to -0.63) |
| Mexico | Chlamydial infection | 3365306.33 | 5656600.68 | 68.09 | 0.27(0.15 to 0.39) |
| Mexico | Gonococcal infection | 817984.69 | 1108512.69 | 35.52 | -0.11(-0.13 to -0.08) |
| Mexico | Trichomoniasis | 4448344.98 | 8935460.61 | 100.87 | 0.09(0.05 to 0.12) |
| Mexico | Genital herpes | 1131379.65 | 1709114.54 | 51.06 | 0.53(0.27 to 0.8) |
| Micronesia (Federated States of) | Sexually transmitted infections excluding HIV | 10565.52 | 12665.65 | 19.88 | -0.02(-0.04 to 0.01) |
| Micronesia (Federated States of) | Syphilis | 350.89 | 413.13 | 17.74 | 0.41(0.3 to 0.52) |
| Micronesia (Federated States of) | Chlamydial infection | 2635.23 | 3025.27 | 14.80 | -0.1(-0.13 to -0.08) |
| Micronesia (Federated States of) | Gonococcal infection | 1174.15 | 1203.77 | 2.52 | -0.3(-0.34 to -0.26) |
| Micronesia (Federated States of) | Trichomoniasis | 5061.30 | 6548.87 | 29.39 | 0.04(0 to 0.07) |
| Micronesia (Federated States of) | Genital herpes | 1343.95 | 1474.62 | 9.72 | 0.04(0.02 to 0.05) |
| Monaco | Sexually transmitted infections excluding HIV | 1178.36 | 1343.85 | 14.04 | -0.01(-0.03 to 0) |
| Monaco | Syphilis | 18.20 | 19.27 | 5.87 | -0.07(-0.09 to -0.05) |
| Monaco | Chlamydial infection | 103.92 | 114.44 | 10.12 | 0.01(-0.01 to 0.02) |
| Monaco | Gonococcal infection | 33.57 | 38.29 | 14.07 | -0.06(-0.07 to -0.05) |
| Monaco | Trichomoniasis | 831.68 | 963.39 | 15.84 | -0.02(-0.04 to 0.01) |
| Monaco | Genital herpes | 191.00 | 208.47 | 9.15 | 0(0 to 0.01) |
| Mongolia | Sexually transmitted infections excluding HIV | 240196.74 | 457144.05 | 90.32 | -0.01(-0.1 to 0.08) |
| Mongolia | Syphilis | 1894.42 | 4080.65 | 115.40 | 1.55(1.25 to 1.86) |
| Mongolia | Chlamydial infection | 103416.59 | 202925.78 | 96.22 | 0(-0.14 to 0.13) |
| Mongolia | Gonococcal infection | 57557.70 | 77274.04 | 34.25 | -0.13(-0.28 to 0.02) |
| Mongolia | Trichomoniasis | 63447.55 | 147170.02 | 131.96 | 0.02(0 to 0.05) |
| Mongolia | Genital herpes | 13880.48 | 25693.56 | 85.11 | 0.01(0 to 0.01) |
| Montenegro | Sexually transmitted infections excluding HIV | 58928.98 | 57629.83 | -2.20 | -0.03(-0.04 to -0.02) |
| Montenegro | Syphilis | 261.56 | 247.12 | -5.52 | -0.02(-0.04 to 0) |
| Montenegro | Chlamydial infection | 21556.08 | 20140.58 | -6.57 | -0.04(-0.05 to -0.02) |
| Montenegro | Gonococcal infection | 12400.81 | 10493.64 | -15.38 | -0.12(-0.16 to -0.09) |
| Montenegro | Trichomoniasis | 21316.04 | 23522.29 | 10.35 | 0.03(0.01 to 0.05) |
| Montenegro | Genital herpes | 3394.49 | 3226.19 | -4.96 | 0(-0.01 to 0.01) |
| Morocco | Sexually transmitted infections excluding HIV | 2697337.42 | 3846231.38 | 42.59 | -0.46(-0.52 to -0.41) |
| Morocco | Syphilis | 47244.59 | 69801.18 | 47.74 | 0.31(-0.08 to 0.7) |
| Morocco | Chlamydial infection | 1273113.61 | 1642842.47 | 29.04 | -1(-1.16 to -0.83) |
| Morocco | Gonococcal infection | 329366.68 | 419379.98 | 27.33 | -0.37(-0.41 to -0.33) |
| Morocco | Trichomoniasis | 758453.57 | 1382734.82 | 82.31 | 0.18(0.05 to 0.32) |
| Morocco | Genital herpes | 289158.97 | 331472.93 | 14.63 | -0.68(-0.76 to -0.59) |
| Mozambique | Sexually transmitted infections excluding HIV | 2047528.71 | 4352589.61 | 112.58 | -0.36(-0.44 to -0.29) |
| Mozambique | Syphilis | 148494.56 | 214339.67 | 44.34 | -1.55(-1.73 to -1.37) |
| Mozambique | Chlamydial infection | 353618.91 | 833294.63 | 135.65 | -0.24(-0.32 to -0.15) |
| Mozambique | Gonococcal infection | 201000.29 | 463411.93 | 130.55 | -0.46(-0.56 to -0.35) |
| Mozambique | Trichomoniasis | 1068559.11 | 2144554.55 | 100.70 | -0.36(-0.48 to -0.24) |
| Mozambique | Genital herpes | 275855.84 | 696988.82 | 152.66 | 0.05(0.02 to 0.07) |
| Myanmar | Sexually transmitted infections excluding HIV | 4362776.73 | 6495316.29 | 48.88 | -0.08(-0.09 to -0.07) |
| Myanmar | Syphilis | 85184.23 | 121610.10 | 42.76 | 0.26(0.05 to 0.47) |
| Myanmar | Chlamydial infection | 1738248.09 | 2538146.79 | 46.02 | -0.07(-0.08 to -0.05) |
| Myanmar | Gonococcal infection | 593749.33 | 744537.10 | 25.40 | -0.38(-0.44 to -0.31) |
| Myanmar | Trichomoniasis | 1462501.08 | 2423532.16 | 65.71 | -0.03(-0.05 to 0) |
| Myanmar | Genital herpes | 483094.01 | 667490.15 | 38.17 | 0.01(0 to 0.02) |
| Namibia | Sexually transmitted infections excluding HIV | 200873.90 | 386921.82 | 92.62 | -0.07(-0.09 to -0.05) |
| Namibia | Syphilis | 7291.73 | 12484.22 | 71.21 | -1.07(-1.43 to -0.72) |
| Namibia | Chlamydial infection | 42933.71 | 83334.90 | 94.10 | -0.05(-0.06 to -0.05) |
| Namibia | Gonococcal infection | 42493.07 | 68861.26 | 62.05 | -0.38(-0.42 to -0.33) |
| Namibia | Trichomoniasis | 76840.45 | 161948.20 | 110.76 | 0.03(-0.04 to 0.1) |
| Namibia | Genital herpes | 31314.94 | 60293.24 | 92.54 | 0.16(0.12 to 0.2) |
| Nauru | Sexually transmitted infections excluding HIV | 1128.10 | 1300.73 | 15.30 | 0.02(-0.01 to 0.04) |
| Nauru | Syphilis | 30.82 | 34.81 | 12.94 | -0.01(-0.03 to 0) |
| Nauru | Chlamydial infection | 277.99 | 320.35 | 15.24 | 0.02(0 to 0.04) |
| Nauru | Gonococcal infection | 105.51 | 116.30 | 10.23 | -0.14(-0.17 to -0.11) |
| Nauru | Trichomoniasis | 576.48 | 673.93 | 16.90 | 0.04(0 to 0.08) |
| Nauru | Genital herpes | 137.30 | 155.34 | 13.14 | 0.02(0 to 0.03) |
| Nepal | Sexually transmitted infections excluding HIV | 1019772.25 | 1827627.01 | 79.22 | -0.15(-0.16 to -0.14) |
| Nepal | Syphilis | 43526.83 | 75045.83 | 72.41 | -0.24(-0.27 to -0.21) |
| Nepal | Chlamydial infection | 323876.72 | 583422.01 | 80.14 | -0.07(-0.08 to -0.06) |
| Nepal | Gonococcal infection | 132019.68 | 209415.05 | 58.62 | -0.48(-0.52 to -0.44) |
| Nepal | Trichomoniasis | 420526.20 | 776175.80 | 84.57 | -0.16(-0.19 to -0.14) |
| Nepal | Genital herpes | 99822.82 | 183568.32 | 83.89 | 0.03(0.02 to 0.04) |
| Netherlands | Sexually transmitted infections excluding HIV | 613562.53 | 637040.18 | 3.83 | 0.09(0.01 to 0.17) |
| Netherlands | Syphilis | 10999.79 | 10195.43 | -7.31 | -0.14(-0.16 to -0.11) |
| Netherlands | Chlamydial infection | 79415.61 | 67511.75 | -14.99 | -0.26(-0.4 to -0.11) |
| Netherlands | Gonococcal infection | 20265.39 | 19765.82 | -2.47 | -0.29(-0.35 to -0.24) |
| Netherlands | Trichomoniasis | 370642.10 | 412575.81 | 11.31 | 0.12(0.01 to 0.22) |
| Netherlands | Genital herpes | 132239.64 | 126991.37 | -3.97 | 0.3(0.11 to 0.48) |
| New Zealand | Sexually transmitted infections excluding HIV | 208667.40 | 257902.63 | 23.60 | -0.03(-0.21 to 0.15) |
| New Zealand | Syphilis | 2469.88 | 2722.40 | 10.22 | -0.11(-0.15 to -0.08) |
| New Zealand | Chlamydial infection | 61895.28 | 72829.61 | 17.67 | 0.08(-0.53 to 0.7) |
| New Zealand | Gonococcal infection | 13006.99 | 14034.48 | 7.90 | -0.19(-0.22 to -0.16) |
| New Zealand | Trichomoniasis | 108858.84 | 142861.76 | 31.24 | -0.06(-0.11 to -0.01) |
| New Zealand | Genital herpes | 22436.40 | 25454.39 | 13.45 | -0.04(-0.09 to 0.01) |
| Nicaragua | Sexually transmitted infections excluding HIV | 403860.16 | 861314.22 | 113.27 | -0.08(-0.11 to -0.04) |
| Nicaragua | Syphilis | 3582.69 | 6458.22 | 80.26 | -0.63(-0.83 to -0.43) |
| Nicaragua | Chlamydial infection | 128483.37 | 259284.14 | 101.80 | -0.21(-0.25 to -0.17) |
| Nicaragua | Gonococcal infection | 47220.90 | 75855.79 | 60.64 | -0.42(-0.45 to -0.38) |
| Nicaragua | Trichomoniasis | 161582.65 | 400781.15 | 148.03 | 0.07(0.02 to 0.11) |
| Nicaragua | Genital herpes | 62990.54 | 118934.92 | 88.81 | -0.04(-0.05 to -0.04) |
| Niger | Sexually transmitted infections excluding HIV | 763217.00 | 2045291.22 | 167.98 | -0.12(-0.15 to -0.09) |
| Niger | Syphilis | 20818.12 | 55344.56 | 165.85 | -0.32(-0.36 to -0.27) |
| Niger | Chlamydial infection | 119435.77 | 323193.32 | 170.60 | -0.12(-0.14 to -0.09) |
| Niger | Gonococcal infection | 123973.86 | 315135.55 | 154.20 | -0.49(-0.54 to -0.44) |
| Niger | Trichomoniasis | 384341.56 | 1020630.84 | 165.55 | -0.05(-0.1 to 0) |
| Niger | Genital herpes | 114647.69 | 330986.95 | 188.70 | -0.01(-0.02 to 0) |
| Nigeria | Sexually transmitted infections excluding HIV | 10112449.70 | 25236951.19 | 149.56 | 0.09(0.03 to 0.14) |
| Nigeria | Syphilis | 382161.09 | 873480.43 | 128.56 | -0.19(-0.29 to -0.1) |
| Nigeria | Chlamydial infection | 1884090.59 | 4800798.35 | 154.81 | 0.81(0.58 to 1.03) |
| Nigeria | Gonococcal infection | 1185934.59 | 2697320.81 | 127.44 | -0.35(-0.37 to -0.32) |
| Nigeria | Trichomoniasis | 5225260.71 | 13272724.69 | 154.01 | -0.05(-0.12 to 0.03) |
| Nigeria | Genital herpes | 1435002.71 | 3592626.91 | 150.36 | -0.01(-0.04 to 0.02) |
| Niue | Sexually transmitted infections excluding HIV | 249.03 | 202.38 | -18.73 | -0.01(-0.04 to 0.01) |
| Niue | Syphilis | 6.29 | 4.68 | -25.62 | 0.01(-0.01 to 0.02) |
| Niue | Chlamydial infection | 58.44 | 45.50 | -22.15 | -0.03(-0.09 to 0.03) |
| Niue | Gonococcal infection | 21.44 | 14.71 | -31.39 | -0.15(-0.17 to -0.12) |
| Niue | Trichomoniasis | 134.63 | 116.98 | -13.11 | 0.01(-0.03 to 0.04) |
| Niue | Genital herpes | 28.23 | 20.52 | -27.32 | 0.01(0 to 0.03) |
| North Macedonia | Sexually transmitted infections excluding HIV | 190137.96 | 212357.91 | 11.69 | 0(-0.01 to 0.01) |
| North Macedonia | Syphilis | 825.07 | 897.93 | 8.83 | 0.07(0.05 to 0.08) |
| North Macedonia | Chlamydial infection | 70856.34 | 76994.85 | 8.66 | -0.01(-0.02 to 0) |
| North Macedonia | Gonococcal infection | 38815.59 | 36113.28 | -6.96 | -0.08(-0.1 to -0.05) |
| North Macedonia | Trichomoniasis | 68619.97 | 86554.66 | 26.14 | 0.05(0.04 to 0.07) |
| North Macedonia | Genital herpes | 11020.99 | 11797.19 | 7.04 | -0.02(-0.03 to -0.02) |
| Northern Mariana Islands | Sexually transmitted infections excluding HIV | 6640.17 | 5406.81 | -18.57 | -0.01(-0.08 to 0.07) |
| Northern Mariana Islands | Syphilis | 191.79 | 131.17 | -31.61 | -0.12(-0.14 to -0.09) |
| Northern Mariana Islands | Chlamydial infection | 1629.76 | 1183.70 | -27.37 | -0.07(-0.23 to 0.1) |
| Northern Mariana Islands | Gonococcal infection | 586.85 | 423.97 | -27.76 | -0.36(-0.44 to -0.29) |
| Northern Mariana Islands | Trichomoniasis | 3447.15 | 3130.61 | -9.18 | 0.07(0.01 to 0.13) |
| Northern Mariana Islands | Genital herpes | 784.62 | 537.36 | -31.51 | 0.01(0 to 0.03) |
| Norway | Sexually transmitted infections excluding HIV | 184650.85 | 233911.47 | 26.68 | 0.03(0 to 0.06) |
| Norway | Syphilis | 3203.14 | 3813.15 | 19.04 | -0.03(-0.05 to -0.02) |
| Norway | Chlamydial infection | 27888.11 | 34555.18 | 23.91 | 0.16(0.03 to 0.3) |
| Norway | Gonococcal infection | 6088.27 | 7752.16 | 27.33 | 0.2(0.13 to 0.26) |
| Norway | Trichomoniasis | 116871.15 | 153132.34 | 31.03 | 0.05(0.03 to 0.08) |
| Norway | Genital herpes | 30600.18 | 34658.63 | 13.26 | -0.18(-0.23 to -0.13) |
| Oman | Sexually transmitted infections excluding HIV | 157069.47 | 534943.74 | 240.58 | -0.01(-0.07 to 0.04) |
| Oman | Syphilis | 1681.14 | 5867.32 | 249.01 | 0.07(-0.11 to 0.25) |
| Oman | Chlamydial infection | 57525.47 | 202876.29 | 252.67 | 0(-0.07 to 0.07) |
| Oman | Gonococcal infection | 22479.98 | 62481.29 | 177.94 | -0.13(-0.19 to -0.07) |
| Oman | Trichomoniasis | 61151.26 | 221107.51 | 261.57 | 0.03(-0.04 to 0.1) |
| Oman | Genital herpes | 14231.62 | 42611.35 | 199.41 | -0.1(-0.19 to -0.02) |
| Pakistan | Sexually transmitted infections excluding HIV | 5886575.53 | 13284376.00 | 125.67 | -0.1(-0.16 to -0.05) |
| Pakistan | Syphilis | 237421.82 | 548102.44 | 130.86 | 0.53(0.28 to 0.77) |
| Pakistan | Chlamydial infection | 2272324.26 | 5040576.78 | 121.82 | -0.24(-0.4 to -0.08) |
| Pakistan | Gonococcal infection | 416946.91 | 898700.85 | 115.54 | -0.2(-0.27 to -0.12) |
| Pakistan | Trichomoniasis | 2505676.55 | 5749905.33 | 129.48 | -0.05(-0.07 to -0.02) |
| Pakistan | Genital herpes | 454205.99 | 1047090.60 | 130.53 | 0.01(-0.04 to 0.06) |
| Palau | Sexually transmitted infections excluding HIV | 1976.16 | 2385.44 | 20.71 | -0.15(-0.21 to -0.09) |
| Palau | Syphilis | 47.69 | 52.57 | 10.22 | 0.16(0.1 to 0.22) |
| Palau | Chlamydial infection | 482.85 | 490.85 | 1.66 | -0.37(-0.49 to -0.24) |
| Palau | Gonococcal infection | 177.10 | 159.38 | -10.01 | -0.16(-0.18 to -0.14) |
| Palau | Trichomoniasis | 1032.97 | 1448.05 | 40.18 | -0.11(-0.17 to -0.05) |
| Palau | Genital herpes | 235.55 | 234.59 | -0.41 | 0.02(0.01 to 0.03) |
| Palestine | Sexually transmitted infections excluding HIV | 136423.56 | 402035.90 | 194.70 | -0.03(-0.12 to 0.06) |
| Palestine | Syphilis | 1487.53 | 4278.91 | 187.65 | -0.05(-0.08 to -0.02) |
| Palestine | Chlamydial infection | 45952.35 | 135982.20 | 195.92 | -0.03(-0.04 to -0.02) |
| Palestine | Gonococcal infection | 22807.26 | 58206.45 | 155.21 | -0.19(-0.23 to -0.14) |
| Palestine | Trichomoniasis | 51441.85 | 162363.42 | 215.63 | 0.01(-0.17 to 0.19) |
| Palestine | Genital herpes | 14734.55 | 41204.91 | 179.65 | -0.02(-0.02 to -0.01) |
| Panama | Sexually transmitted infections excluding HIV | 277871.03 | 523571.00 | 88.42 | 0.06(0.04 to 0.07) |
| Panama | Syphilis | 3430.90 | 6467.51 | 88.51 | 0.56(0.44 to 0.67) |
| Panama | Chlamydial infection | 87173.90 | 152929.48 | 75.43 | -0.04(-0.05 to -0.04) |
| Panama | Gonococcal infection | 19012.00 | 30861.66 | 62.33 | 0.1(0.04 to 0.16) |
| Panama | Trichomoniasis | 125612.48 | 263816.84 | 110.02 | 0.11(0.07 to 0.15) |
| Panama | Genital herpes | 42641.75 | 69495.51 | 62.98 | 0(-0.01 to 0) |
| Papua New Guinea | Sexually transmitted infections excluding HIV | 574883.06 | 1424794.35 | 147.84 | -0.05(-0.1 to 0) |
| Papua New Guinea | Syphilis | 20293.45 | 45696.78 | 125.18 | -0.9(-1.14 to -0.67) |
| Papua New Guinea | Chlamydial infection | 168279.71 | 375626.49 | 123.22 | -0.6(-0.75 to -0.46) |
| Papua New Guinea | Gonococcal infection | 90379.44 | 202948.79 | 124.55 | 0.22(0.09 to 0.35) |
| Papua New Guinea | Trichomoniasis | 235023.79 | 647511.15 | 175.51 | 0.24(0.15 to 0.32) |
| Papua New Guinea | Genital herpes | 60906.68 | 153011.13 | 151.22 | -0.08(-0.28 to 0.12) |
| Paraguay | Sexually transmitted infections excluding HIV | 447616.24 | 927909.83 | 107.30 | 0(-0.01 to 0.02) |
| Paraguay | Syphilis | 7639.94 | 16844.68 | 120.48 | 1.04(0.75 to 1.33) |
| Paraguay | Chlamydial infection | 160542.88 | 324141.18 | 101.90 | -0.04(-0.05 to -0.03) |
| Paraguay | Gonococcal infection | 35711.35 | 63709.12 | 78.40 | -0.2(-0.28 to -0.12) |
| Paraguay | Trichomoniasis | 167727.10 | 378029.44 | 125.38 | 0.03(-0.01 to 0.08) |
| Paraguay | Genital herpes | 75994.97 | 145185.40 | 91.05 | -0.01(-0.02 to 0) |
| Peru | Sexually transmitted infections excluding HIV | 1870418.02 | 3375184.43 | 80.45 | 0.02(-0.01 to 0.04) |
| Peru | Syphilis | 45005.84 | 66385.21 | 47.50 | -0.71(-0.87 to -0.56) |
| Peru | Chlamydial infection | 570313.84 | 1005850.79 | 76.37 | 0.26(0.17 to 0.35) |
| Peru | Gonococcal infection | 59894.40 | 90937.65 | 51.83 | 0.02(-0.23 to 0.26) |
| Peru | Trichomoniasis | 789502.23 | 1590669.20 | 101.48 | -0.02(-0.07 to 0.02) |
| Peru | Genital herpes | 405701.70 | 621341.58 | 53.15 | -0.19(-0.31 to -0.07) |
| Philippines | Sexually transmitted infections excluding HIV | 6711259.80 | 13430758.58 | 100.12 | -0.03(-0.05 to -0.02) |
| Philippines | Syphilis | 68864.99 | 139003.09 | 101.85 | 0.43(0.35 to 0.51) |
| Philippines | Chlamydial infection | 2632705.05 | 5145983.23 | 95.46 | -0.04(-0.07 to -0.02) |
| Philippines | Gonococcal infection | 889329.41 | 1691872.50 | 90.24 | -0.06(-0.14 to 0.02) |
| Philippines | Trichomoniasis | 2450685.46 | 5190166.67 | 111.78 | -0.03(-0.05 to -0.02) |
| Philippines | Genital herpes | 669674.89 | 1263733.09 | 88.71 | 0.02(-0.01 to 0.04) |
| Poland | Sexually transmitted infections excluding HIV | 3737739.91 | 3808919.05 | 1.90 | -0.06(-0.08 to -0.05) |
| Poland | Syphilis | 16499.70 | 15803.99 | -4.22 | -0.11(-0.15 to -0.07) |
| Poland | Chlamydial infection | 1321351.98 | 1314709.91 | -0.50 | -0.03(-0.04 to -0.03) |
| Poland | Gonococcal infection | 708823.31 | 579659.06 | -18.22 | -0.36(-0.39 to -0.34) |
| Poland | Trichomoniasis | 1509395.15 | 1718119.24 | 13.83 | 0.06(0.04 to 0.08) |
| Poland | Genital herpes | 181669.76 | 180626.86 | -0.57 | 0(-0.09 to 0.09) |
| Portugal | Sexually transmitted infections excluding HIV | 401317.41 | 432574.12 | 7.79 | -0.01(-0.03 to 0.02) |
| Portugal | Syphilis | 6801.99 | 6028.31 | -11.37 | -0.2(-0.23 to -0.17) |
| Portugal | Chlamydial infection | 46613.84 | 46095.93 | -1.11 | 0.01(-0.14 to 0.17) |
| Portugal | Gonococcal infection | 13652.52 | 12057.95 | -11.68 | -0.22(-0.26 to -0.18) |
| Portugal | Trichomoniasis | 263455.44 | 303032.22 | 15.02 | 0(-0.03 to 0.02) |
| Portugal | Genital herpes | 70793.61 | 65359.71 | -7.68 | 0.01(0.01 to 0.02) |
| Puerto Rico | Sexually transmitted infections excluding HIV | 453214.39 | 441284.07 | -2.63 | 0.03(0.01 to 0.04) |
| Puerto Rico | Syphilis | 5526.87 | 4839.10 | -12.44 | 0.02(-0.01 to 0.05) |
| Puerto Rico | Chlamydial infection | 161049.70 | 146730.73 | -8.89 | 0(-0.01 to 0.01) |
| Puerto Rico | Gonococcal infection | 43641.06 | 35395.85 | -18.89 | -0.17(-0.23 to -0.11) |
| Puerto Rico | Trichomoniasis | 182276.50 | 203784.81 | 11.80 | 0.11(0.08 to 0.14) |
| Puerto Rico | Genital herpes | 60720.26 | 50533.59 | -16.78 | -0.02(-0.03 to 0) |
| Qatar | Sexually transmitted infections excluding HIV | 51698.86 | 393781.06 | 661.68 | 0.04(0 to 0.07) |
| Qatar | Syphilis | 772.62 | 6215.34 | 704.45 | 0.28(0.18 to 0.38) |
| Qatar | Chlamydial infection | 19946.79 | 150718.86 | 655.60 | 0.05(-0.01 to 0.11) |
| Qatar | Gonococcal infection | 6091.65 | 44045.26 | 623.04 | 0(-0.06 to 0.05) |
| Qatar | Trichomoniasis | 21013.42 | 165819.00 | 689.11 | 0.08(0.05 to 0.11) |
| Qatar | Genital herpes | 3874.36 | 26982.60 | 596.44 | -0.25(-0.32 to -0.18) |
| Republic of Korea | Sexually transmitted infections excluding HIV | 3009015.07 | 3480434.24 | 15.67 | -0.12(-0.18 to -0.07) |
| Republic of Korea | Syphilis | 36729.03 | 43964.44 | 19.70 | 0.89(0.72 to 1.05) |
| Republic of Korea | Chlamydial infection | 520938.03 | 607255.87 | 16.57 | 0.23(0.1 to 0.37) |
| Republic of Korea | Gonococcal infection | 331431.67 | 307453.49 | -7.23 | -0.15(-0.27 to -0.02) |
| Republic of Korea | Trichomoniasis | 1430664.28 | 2067722.19 | 44.53 | -0.02(-0.06 to 0.03) |
| Republic of Korea | Genital herpes | 689252.07 | 454038.26 | -34.13 | -0.88(-1.1 to -0.66) |
| Republic of Moldova | Sexually transmitted infections excluding HIV | 443694.76 | 384330.01 | -13.38 | -0.03(-0.04 to -0.02) |
| Republic of Moldova | Syphilis | 2507.80 | 2173.69 | -13.32 | 0.28(0.19 to 0.36) |
| Republic of Moldova | Chlamydial infection | 176895.22 | 153553.32 | -13.20 | -0.01(-0.02 to 0) |
| Republic of Moldova | Gonococcal infection | 86936.06 | 60982.84 | -29.85 | -0.26(-0.29 to -0.23) |
| Republic of Moldova | Trichomoniasis | 134259.59 | 133424.24 | -0.62 | 0.07(0.05 to 0.1) |
| Republic of Moldova | Genital herpes | 43096.07 | 34195.92 | -20.65 | -0.01(-0.02 to 0) |
| Romania | Sexually transmitted infections excluding HIV | 2133838.32 | 1722391.06 | -19.28 | -0.04(-0.06 to -0.02) |
| Romania | Syphilis | 13325.71 | 10440.27 | -21.65 | 0.24(0.16 to 0.31) |
| Romania | Chlamydial infection | 760265.14 | 602248.23 | -20.78 | -0.01(-0.02 to 0.01) |
| Romania | Gonococcal infection | 438188.95 | 270390.01 | -38.29 | -0.27(-0.35 to -0.19) |
| Romania | Trichomoniasis | 799584.64 | 744450.36 | -6.90 | 0.05(0.04 to 0.06) |
| Romania | Genital herpes | 122473.88 | 94862.17 | -22.54 | -0.02(-0.03 to -0.02) |
| Russian Federation | Sexually transmitted infections excluding HIV | 15805373.04 | 15015621.75 | -5.00 | -0.04(-0.05 to -0.02) |
| Russian Federation | Syphilis | 80292.03 | 73579.51 | -8.36 | -0.13(-0.16 to -0.1) |
| Russian Federation | Chlamydial infection | 5880758.23 | 5573540.60 | -5.22 | -0.01(-0.02 to 0) |
| Russian Federation | Gonococcal infection | 3139991.67 | 2579777.72 | -17.84 | -0.16(-0.21 to -0.11) |
| Russian Federation | Trichomoniasis | 5252948.59 | 5494804.26 | 4.60 | 0.01(-0.03 to 0.04) |
| Russian Federation | Genital herpes | 1451382.52 | 1293919.66 | -10.85 | 0.01(0.01 to 0.02) |
| Rwanda | Sexually transmitted infections excluding HIV | 930563.95 | 1897594.79 | 103.92 | -0.12(-0.15 to -0.09) |
| Rwanda | Syphilis | 32301.91 | 51631.67 | 59.84 | -0.86(-0.94 to -0.78) |
| Rwanda | Chlamydial infection | 184756.06 | 378504.39 | 104.87 | -0.06(-0.09 to -0.04) |
| Rwanda | Gonococcal infection | 95562.79 | 172917.28 | 80.95 | -0.37(-0.4 to -0.33) |
| Rwanda | Trichomoniasis | 477219.92 | 1016870.60 | 113.08 | -0.1(-0.16 to -0.03) |
| Rwanda | Genital herpes | 140723.27 | 277670.85 | 97.32 | 0.04(-0.06 to 0.15) |
| Saint Kitts and Nevis | Sexually transmitted infections excluding HIV | 4796.48 | 8459.29 | 76.36 | 0.01(0 to 0.02) |
| Saint Kitts and Nevis | Syphilis | 63.65 | 96.88 | 52.20 | -0.09(-0.13 to -0.06) |
| Saint Kitts and Nevis | Chlamydial infection | 1728.01 | 2950.18 | 70.73 | -0.01(-0.02 to -0.01) |
| Saint Kitts and Nevis | Gonococcal infection | 526.94 | 692.41 | 31.40 | -0.15(-0.2 to -0.1) |
| Saint Kitts and Nevis | Trichomoniasis | 1780.34 | 3717.58 | 108.81 | 0.07(0.04 to 0.09) |
| Saint Kitts and Nevis | Genital herpes | 697.53 | 1002.25 | 43.68 | 0.02(0.01 to 0.03) |
| Saint Lucia | Sexually transmitted infections excluding HIV | 15628.12 | 24980.55 | 59.84 | 0(-0.01 to 0.02) |
| Saint Lucia | Syphilis | 280.90 | 380.36 | 35.41 | 0.24(0.15 to 0.32) |
| Saint Lucia | Chlamydial infection | 5638.30 | 8712.37 | 54.52 | -0.02(-0.03 to -0.02) |
| Saint Lucia | Gonococcal infection | 1954.01 | 2212.79 | 13.24 | -0.25(-0.31 to -0.18) |
| Saint Lucia | Trichomoniasis | 5447.12 | 10714.89 | 96.71 | 0.09(0.06 to 0.12) |
| Saint Lucia | Genital herpes | 2307.80 | 2960.13 | 28.27 | -0.03(-0.04 to -0.02) |
| Saint Vincent and the Grenadines | Sexually transmitted infections excluding HIV | 12525.69 | 15097.68 | 20.53 | 0(-0.02 to 0.01) |
| Saint Vincent and the Grenadines | Syphilis | 185.78 | 179.28 | -3.50 | -0.38(-0.49 to -0.28) |
| Saint Vincent and the Grenadines | Chlamydial infection | 4516.13 | 5268.93 | 16.67 | -0.02(-0.03 to -0.01) |
| Saint Vincent and the Grenadines | Gonococcal infection | 1624.40 | 1428.66 | -12.05 | -0.25(-0.31 to -0.18) |
| Saint Vincent and the Grenadines | Trichomoniasis | 4338.58 | 6397.59 | 47.46 | 0.08(0.06 to 0.11) |
| Saint Vincent and the Grenadines | Genital herpes | 1860.80 | 1823.22 | -2.02 | 0.01(0 to 0.02) |
| Samoa | Sexually transmitted infections excluding HIV | 19005.28 | 26902.49 | 41.55 | 0.07(-0.02 to 0.17) |
| Samoa | Syphilis | 358.57 | 458.34 | 27.83 | -0.13(-0.16 to -0.1) |
| Samoa | Chlamydial infection | 6052.69 | 8391.21 | 38.64 | 0.19(-0.09 to 0.46) |
| Samoa | Gonococcal infection | 2187.23 | 2652.42 | 21.27 | -0.17(-0.25 to -0.09) |
| Samoa | Trichomoniasis | 8166.30 | 12510.09 | 53.19 | 0.06(0.03 to 0.1) |
| Samoa | Genital herpes | 2240.49 | 2890.43 | 29.01 | 0.01(0 to 0.03) |
| San Marino | Sexually transmitted infections excluding HIV | 904.00 | 1238.33 | 36.98 | -0.1(-0.11 to -0.09) |
| San Marino | Syphilis | 16.13 | 19.25 | 19.35 | -0.17(-0.2 to -0.14) |
| San Marino | Chlamydial infection | 86.46 | 112.73 | 30.40 | -0.07(-0.08 to -0.05) |
| San Marino | Gonococcal infection | 30.21 | 37.61 | 24.50 | -0.11(-0.13 to -0.1) |
| San Marino | Trichomoniasis | 603.31 | 855.00 | 41.72 | -0.15(-0.16 to -0.13) |
| San Marino | Genital herpes | 167.90 | 213.73 | 27.30 | 0.03(0.02 to 0.04) |
| Sao Tome and Principe | Sexually transmitted infections excluding HIV | 10940.39 | 23694.00 | 116.57 | -0.02(-0.05 to 0.02) |
| Sao Tome and Principe | Syphilis | 397.48 | 796.54 | 100.40 | -0.14(-0.22 to -0.05) |
| Sao Tome and Principe | Chlamydial infection | 1753.78 | 3778.21 | 115.43 | -0.03(-0.04 to -0.02) |
| Sao Tome and Principe | Gonococcal infection | 1454.84 | 2717.38 | 86.78 | -0.1(-0.13 to -0.08) |
| Sao Tome and Principe | Trichomoniasis | 5590.23 | 12922.24 | 131.16 | 0.01(-0.05 to 0.07) |
| Sao Tome and Principe | Genital herpes | 1744.07 | 3479.63 | 99.51 | -0.01(-0.02 to 0) |
| Saudi Arabia | Sexually transmitted infections excluding HIV | 1669183.11 | 4962092.77 | 197.28 | -0.21(-0.29 to -0.14) |
| Saudi Arabia | Syphilis | 13182.80 | 39336.62 | 198.39 | -0.12(-0.14 to -0.1) |
| Saudi Arabia | Chlamydial infection | 706710.59 | 1830199.69 | 158.97 | -0.38(-0.48 to -0.28) |
| Saudi Arabia | Gonococcal infection | 177107.15 | 413850.00 | 133.67 | -0.24(-0.27 to -0.21) |
| Saudi Arabia | Trichomoniasis | 648606.40 | 2331346.32 | 259.44 | -0.11(-0.24 to 0.02) |
| Saudi Arabia | Genital herpes | 123576.18 | 347360.13 | 181.09 | 0.01(-0.01 to 0.03) |
| Senegal | Sexually transmitted infections excluding HIV | 630855.08 | 1372995.12 | 117.64 | -0.14(-0.29 to 0.01) |
| Senegal | Syphilis | 34491.04 | 73844.24 | 114.10 | -0.41(-0.49 to -0.32) |
| Senegal | Chlamydial infection | 86235.41 | 153393.74 | 77.88 | -0.52(-1.42 to 0.38) |
| Senegal | Gonococcal infection | 38739.15 | 74962.20 | 93.51 | -0.16(-0.99 to 0.67) |
| Senegal | Trichomoniasis | 372812.60 | 853579.37 | 128.96 | -0.07(-0.13 to -0.02) |
| Senegal | Genital herpes | 98576.88 | 217215.57 | 120.35 | 0.01(-0.01 to 0.03) |
| Serbia | Sexually transmitted infections excluding HIV | 875136.73 | 785296.91 | -10.27 | -0.04(-0.05 to -0.03) |
| Serbia | Syphilis | 3767.66 | 3272.41 | -13.14 | -0.03(-0.05 to -0.01) |
| Serbia | Chlamydial infection | 315839.80 | 275153.74 | -12.88 | -0.04(-0.06 to -0.03) |
| Serbia | Gonococcal infection | 168157.36 | 138045.70 | -17.91 | -0.13(-0.16 to -0.09) |
| Serbia | Trichomoniasis | 337228.95 | 325132.31 | -3.59 | 0.02(0 to 0.03) |
| Serbia | Genital herpes | 50142.96 | 43692.76 | -12.86 | -0.01(-0.02 to 0) |
| Seychelles | Sexually transmitted infections excluding HIV | 7894.66 | 12976.02 | 64.36 | 0.05(0.04 to 0.07) |
| Seychelles | Syphilis | 62.79 | 90.97 | 44.87 | 0.04(-0.01 to 0.08) |
| Seychelles | Chlamydial infection | 3121.83 | 4974.54 | 59.35 | 0.07(0.05 to 0.08) |
| Seychelles | Gonococcal infection | 1045.64 | 1428.48 | 36.61 | 0.09(0.05 to 0.13) |
| Seychelles | Trichomoniasis | 2773.94 | 5279.84 | 90.34 | 0.06(0.04 to 0.08) |
| Seychelles | Genital herpes | 890.46 | 1202.19 | 35.01 | -0.05(-0.06 to -0.04) |
| Sierra Leone | Sexually transmitted infections excluding HIV | 371037.18 | 900940.94 | 142.82 | -0.06(-0.09 to -0.03) |
| Sierra Leone | Syphilis | 12988.70 | 32047.92 | 146.74 | -0.03(-0.11 to 0.05) |
| Sierra Leone | Chlamydial infection | 60004.63 | 147638.87 | 146.05 | -0.06(-0.08 to -0.04) |
| Sierra Leone | Gonococcal infection | 45235.79 | 110343.40 | 143.93 | -0.2(-0.23 to -0.16) |
| Sierra Leone | Trichomoniasis | 197011.79 | 469621.94 | 138.37 | -0.05(-0.1 to 0.01) |
| Sierra Leone | Genital herpes | 55796.27 | 141288.81 | 153.22 | -0.04(-0.06 to -0.02) |
| Singapore | Sexually transmitted infections excluding HIV | 200852.58 | 387670.52 | 93.01 | 0.03(0.02 to 0.04) |
| Singapore | Syphilis | 2924.00 | 4938.71 | 68.90 | 0.02(0 to 0.04) |
| Singapore | Chlamydial infection | 40519.99 | 70604.28 | 74.25 | 0.03(0 to 0.05) |
| Singapore | Gonococcal infection | 22516.82 | 30996.04 | 37.66 | -0.35(-0.38 to -0.31) |
| Singapore | Trichomoniasis | 107446.76 | 236133.62 | 119.77 | 0.11(0.09 to 0.13) |
| Singapore | Genital herpes | 27445.00 | 44997.88 | 63.96 | -0.01(-0.03 to 0.02) |
| Slovakia | Sexually transmitted infections excluding HIV | 485752.19 | 517756.56 | 6.59 | 0.01(0 to 0.02) |
| Slovakia | Syphilis | 1887.26 | 1955.01 | 3.59 | 0.02(0 to 0.04) |
| Slovakia | Chlamydial infection | 178197.51 | 184125.89 | 3.33 | -0.02(-0.02 to -0.01) |
| Slovakia | Gonococcal infection | 96002.31 | 83682.71 | -12.83 | -0.05(-0.08 to -0.02) |
| Slovakia | Trichomoniasis | 181398.72 | 219258.57 | 20.87 | 0.06(0.05 to 0.08) |
| Slovakia | Genital herpes | 28266.39 | 28734.38 | 1.66 | -0.02(-0.02 to -0.01) |
| Slovenia | Sexually transmitted infections excluding HIV | 173128.16 | 166602.11 | -3.77 | -0.04(-0.06 to -0.01) |
| Slovenia | Syphilis | 821.83 | 765.37 | -6.87 | 0.03(0.02 to 0.04) |
| Slovenia | Chlamydial infection | 53685.36 | 49486.48 | -7.82 | 0.07(-0.01 to 0.14) |
| Slovenia | Gonococcal infection | 35311.64 | 25436.88 | -27.96 | -0.38(-0.41 to -0.35) |
| Slovenia | Trichomoniasis | 72430.79 | 80894.68 | 11.69 | 0.06(0.05 to 0.08) |
| Slovenia | Genital herpes | 10878.54 | 10018.69 | -7.90 | -0.02(-0.03 to -0.02) |
| Solomon Islands | Sexually transmitted infections excluding HIV | 31558.71 | 71843.53 | 127.65 | 0.01(-0.01 to 0.02) |
| Solomon Islands | Syphilis | 1404.00 | 2802.37 | 99.60 | -0.11(-0.21 to -0.01) |
| Solomon Islands | Chlamydial infection | 6891.57 | 15318.02 | 122.27 | 0(-0.02 to 0.03) |
| Solomon Islands | Gonococcal infection | 3212.98 | 6051.08 | 88.33 | -0.35(-0.41 to -0.29) |
| Solomon Islands | Trichomoniasis | 15715.95 | 38655.96 | 145.97 | 0.06(0.03 to 0.09) |
| Solomon Islands | Genital herpes | 4334.20 | 9016.10 | 108.02 | 0.01(0 to 0.03) |
| Somalia | Sexually transmitted infections excluding HIV | 991936.80 | 2803667.37 | 182.65 | -0.19(-0.29 to -0.1) |
| Somalia | Syphilis | 39466.11 | 112870.99 | 185.99 | -0.07(-0.2 to 0.05) |
| Somalia | Chlamydial infection | 265392.92 | 727694.13 | 174.20 | -0.58(-0.78 to -0.37) |
| Somalia | Gonococcal infection | 54009.73 | 165459.10 | 206.35 | -0.05(-0.2 to 0.09) |
| Somalia | Trichomoniasis | 499338.45 | 1381372.56 | 176.64 | -0.08(-0.14 to -0.01) |
| Somalia | Genital herpes | 133729.59 | 416270.57 | 211.28 | 0(-0.02 to 0.01) |
| South Africa | Sexually transmitted infections excluding HIV | 8483636.11 | 13338763.84 | 57.23 | -0.29(-0.36 to -0.22) |
| South Africa | Syphilis | 428839.14 | 465084.01 | 8.45 | -1.34(-1.97 to -0.7) |
| South Africa | Chlamydial infection | 2331890.17 | 3946854.10 | 69.26 | 0.11(-0.07 to 0.29) |
| South Africa | Gonococcal infection | 2013099.04 | 2749734.51 | 36.59 | -0.47(-0.65 to -0.29) |
| South Africa | Trichomoniasis | 2832351.18 | 4777073.44 | 68.66 | -0.59(-0.76 to -0.42) |
| South Africa | Genital herpes | 877456.58 | 1400017.78 | 59.55 | 0.34(0.21 to 0.46) |
| South Sudan | Sexually transmitted infections excluding HIV | 830793.33 | 1262391.10 | 51.95 | -0.12(-0.15 to -0.1) |
| South Sudan | Syphilis | 38416.25 | 54670.64 | 42.31 | 0.15(0.03 to 0.26) |
| South Sudan | Chlamydial infection | 169443.82 | 243774.10 | 43.87 | -0.13(-0.15 to -0.12) |
| South Sudan | Gonococcal infection | 86846.85 | 125191.63 | 44.15 | -0.22(-0.26 to -0.19) |
| South Sudan | Trichomoniasis | 413269.53 | 653479.11 | 58.12 | -0.15(-0.2 to -0.11) |
| South Sudan | Genital herpes | 122816.88 | 185275.62 | 50.86 | 0.01(-0.01 to 0.02) |
| Spain | Sexually transmitted infections excluding HIV | 1456809.80 | 1838281.66 | 26.19 | 0.08(0.05 to 0.11) |
| Spain | Syphilis | 27330.74 | 26948.80 | -1.40 | -0.22(-0.24 to -0.2) |
| Spain | Chlamydial infection | 179414.44 | 210095.92 | 17.10 | 0.22(0.12 to 0.32) |
| Spain | Gonococcal infection | 65699.75 | 63227.79 | -3.76 | -0.23(-0.27 to -0.19) |
| Spain | Trichomoniasis | 951786.98 | 1278209.36 | 34.30 | 0.05(0.03 to 0.07) |
| Spain | Genital herpes | 232577.89 | 259799.80 | 11.70 | 0.21(0.13 to 0.28) |
| Sri Lanka | Sexually transmitted infections excluding HIV | 2074338.63 | 2662807.84 | 28.37 | -0.04(-0.07 to -0.02) |
| Sri Lanka | Syphilis | 5150.08 | 5646.83 | 9.65 | -0.67(-1.08 to -0.26) |
| Sri Lanka | Chlamydial infection | 775227.78 | 936460.61 | 20.80 | -0.07(-0.09 to -0.05) |
| Sri Lanka | Gonococcal infection | 264752.39 | 301884.48 | 14.03 | -0.06(-0.12 to 0.01) |
| Sri Lanka | Trichomoniasis | 814584.03 | 1170091.08 | 43.64 | -0.03(-0.07 to 0.01) |
| Sri Lanka | Genital herpes | 214624.35 | 248724.84 | 15.89 | 0.01(0 to 0.02) |
| Sudan | Sexually transmitted infections excluding HIV | 1670429.95 | 4058636.93 | 142.97 | -0.17(-0.32 to -0.01) |
| Sudan | Syphilis | 24815.89 | 60230.36 | 142.71 | -0.22(-0.4 to -0.04) |
| Sudan | Chlamydial infection | 642934.00 | 1653507.68 | 157.18 | 0.08(-0.14 to 0.3) |
| Sudan | Gonococcal infection | 291192.86 | 606054.65 | 108.13 | -0.3(-0.39 to -0.21) |
| Sudan | Trichomoniasis | 560777.34 | 1399252.77 | 149.52 | -0.38(-0.62 to -0.13) |
| Sudan | Genital herpes | 150709.87 | 339591.46 | 125.33 | -0.01(-0.02 to 0) |
| Suriname | Sexually transmitted infections excluding HIV | 47490.72 | 75925.08 | 59.87 | -0.01(-0.03 to 0.01) |
| Suriname | Syphilis | 447.20 | 644.86 | 44.20 | -0.12(-0.13 to -0.1) |
| Suriname | Chlamydial infection | 17477.10 | 26938.53 | 54.14 | -0.02(-0.03 to -0.01) |
| Suriname | Gonococcal infection | 5671.26 | 7022.03 | 23.82 | -0.33(-0.41 to -0.25) |
| Suriname | Trichomoniasis | 17248.32 | 31941.83 | 85.19 | 0.06(0.04 to 0.09) |
| Suriname | Genital herpes | 6646.85 | 9377.84 | 41.09 | 0.05(0.04 to 0.06) |
| Sweden | Sexually transmitted infections excluding HIV | 398686.50 | 458730.42 | 15.06 | -0.07(-0.14 to -0.01) |
| Sweden | Syphilis | 5936.69 | 6543.91 | 10.23 | -0.06(-0.08 to -0.03) |
| Sweden | Chlamydial infection | 73401.56 | 80633.22 | 9.85 | -0.25(-0.47 to -0.03) |
| Sweden | Gonococcal infection | 12192.00 | 13204.99 | 8.31 | -0.11(-0.14 to -0.07) |
| Sweden | Trichomoniasis | 240683.69 | 283770.73 | 17.90 | 0.02(0 to 0.04) |
| Sweden | Genital herpes | 66472.55 | 74577.56 | 12.19 | -0.19(-0.31 to -0.06) |
| Switzerland | Sexually transmitted infections excluding HIV | 287418.10 | 346501.12 | 20.56 | -0.08(-0.1 to -0.05) |
| Switzerland | Syphilis | 4952.47 | 5482.91 | 10.71 | -0.06(-0.08 to -0.04) |
| Switzerland | Chlamydial infection | 24934.66 | 28900.19 | 15.90 | -0.04(-0.06 to -0.02) |
| Switzerland | Gonococcal infection | 8795.69 | 9965.38 | 13.30 | -0.15(-0.17 to -0.13) |
| Switzerland | Trichomoniasis | 189525.53 | 241496.77 | 27.42 | 0.01(-0.01 to 0.02) |
| Switzerland | Genital herpes | 59209.75 | 60655.87 | 2.44 | -0.34(-0.42 to -0.27) |
| Syrian Arab Republic | Sexually transmitted infections excluding HIV | 828153.36 | 1127265.15 | 36.12 | -0.15(-0.17 to -0.12) |
| Syrian Arab Republic | Syphilis | 9593.32 | 11355.01 | 18.36 | -0.29(-0.37 to -0.2) |
| Syrian Arab Republic | Chlamydial infection | 297423.92 | 368528.03 | 23.91 | -0.13(-0.17 to -0.1) |
| Syrian Arab Republic | Gonococcal infection | 143431.06 | 156305.12 | 8.98 | -0.44(-0.51 to -0.38) |
| Syrian Arab Republic | Trichomoniasis | 300425.48 | 487186.92 | 62.17 | -0.12(-0.15 to -0.09) |
| Syrian Arab Republic | Genital herpes | 77279.59 | 103890.08 | 34.43 | 0.13(0.11 to 0.16) |
| Taiwan (Province of China) | Sexually transmitted infections excluding HIV | 2544137.68 | 2915010.50 | 14.58 | -0.04(-0.06 to -0.01) |
| Taiwan (Province of China) | Syphilis | 21918.92 | 22402.86 | 2.21 | -0.08(-0.1 to -0.06) |
| Taiwan (Province of China) | Chlamydial infection | 1273775.74 | 1317203.98 | 3.41 | -0.12(-0.17 to -0.07) |
| Taiwan (Province of China) | Gonococcal infection | 206164.50 | 179945.14 | -12.72 | -0.17(-0.18 to -0.15) |
| Taiwan (Province of China) | Trichomoniasis | 868431.72 | 1212666.60 | 39.64 | 0.09(0.05 to 0.13) |
| Taiwan (Province of China) | Genital herpes | 173846.79 | 182791.93 | 5.15 | 0(-0.01 to 0.01) |
| Tajikistan | Sexually transmitted infections excluding HIV | 587344.05 | 1233764.87 | 110.06 | -0.05(-0.07 to -0.03) |
| Tajikistan | Syphilis | 2715.59 | 4940.51 | 81.93 | -0.75(-0.87 to -0.62) |
| Tajikistan | Chlamydial infection | 248198.79 | 531814.55 | 114.27 | -0.03(-0.05 to -0.02) |
| Tajikistan | Gonococcal infection | 150024.91 | 270626.86 | 80.39 | -0.18(-0.21 to -0.14) |
| Tajikistan | Trichomoniasis | 153170.61 | 357461.48 | 133.37 | 0.02(-0.02 to 0.05) |
| Tajikistan | Genital herpes | 33234.15 | 68921.47 | 107.38 | -0.01(-0.02 to 0) |
| Thailand | Sexually transmitted infections excluding HIV | 7217100.14 | 8972541.44 | 24.32 | -0.06(-0.09 to -0.03) |
| Thailand | Syphilis | 84577.68 | 86969.11 | 2.83 | -0.89(-1.19 to -0.6) |
| Thailand | Chlamydial infection | 3061710.79 | 3513562.51 | 14.76 | 0.16(0.02 to 0.3) |
| Thailand | Gonococcal infection | 815986.53 | 824920.56 | 1.09 | -0.29(-0.44 to -0.14) |
| Thailand | Trichomoniasis | 2394060.04 | 3716396.88 | 55.23 | -0.02(-0.04 to 0) |
| Thailand | Genital herpes | 860765.10 | 830692.38 | -3.49 | -0.64(-0.8 to -0.48) |
| Timor-Leste | Sexually transmitted infections excluding HIV | 83081.37 | 134674.50 | 62.10 | -0.11(-0.13 to -0.1) |
| Timor-Leste | Syphilis | 735.71 | 1228.86 | 67.03 | 0.04(0.02 to 0.05) |
| Timor-Leste | Chlamydial infection | 33754.72 | 52484.86 | 55.49 | -0.12(-0.14 to -0.1) |
| Timor-Leste | Gonococcal infection | 11198.17 | 18487.31 | 65.09 | -0.42(-0.49 to -0.35) |
| Timor-Leste | Trichomoniasis | 27404.73 | 45485.18 | 65.98 | -0.04(-0.06 to -0.01) |
| Timor-Leste | Genital herpes | 9988.05 | 16988.29 | 70.09 | -0.03(-0.12 to 0.05) |
| Togo | Sexually transmitted infections excluding HIV | 323613.00 | 816187.16 | 152.21 | -0.08(-0.13 to -0.04) |
| Togo | Syphilis | 12808.90 | 27919.96 | 117.97 | -0.89(-1.09 to -0.68) |
| Togo | Chlamydial infection | 57402.38 | 141212.28 | 146.00 | -0.04(-0.05 to -0.02) |
| Togo | Gonococcal infection | 46065.72 | 99381.55 | 115.74 | -0.24(-0.28 to -0.19) |
| Togo | Trichomoniasis | 151579.09 | 417172.58 | 175.22 | -0.04(-0.13 to 0.04) |
| Togo | Genital herpes | 55756.91 | 130500.79 | 134.05 | 0.02(0 to 0.04) |
| Tokelau | Sexually transmitted infections excluding HIV | 167.72 | 160.20 | -4.49 | -0.11(-0.14 to -0.08) |
| Tokelau | Syphilis | 4.33 | 3.92 | -9.45 | 0.04(0.01 to 0.07) |
| Tokelau | Chlamydial infection | 40.75 | 37.10 | -8.96 | -0.26(-0.32 to -0.2) |
| Tokelau | Gonococcal infection | 15.56 | 12.93 | -16.88 | -0.22(-0.25 to -0.19) |
| Tokelau | Trichomoniasis | 87.47 | 88.84 | 1.56 | -0.06(-0.09 to -0.02) |
| Tokelau | Genital herpes | 19.61 | 17.40 | -11.24 | 0.02(0 to 0.03) |
| Tonga | Sexually transmitted infections excluding HIV | 10455.32 | 12242.26 | 17.09 | 0.01(-0.04 to 0.06) |
| Tonga | Syphilis | 311.98 | 336.61 | 7.89 | 0(-0.04 to 0.04) |
| Tonga | Chlamydial infection | 2790.40 | 3181.06 | 14.00 | 0.01(-0.16 to 0.18) |
| Tonga | Gonococcal infection | 1293.55 | 1263.34 | -2.34 | -0.21(-0.29 to -0.13) |
| Tonga | Trichomoniasis | 4803.32 | 6121.07 | 27.43 | 0.06(0.02 to 0.09) |
| Tonga | Genital herpes | 1256.08 | 1340.19 | 6.70 | 0.01(0 to 0.03) |
| Trinidad and Tobago | Sexually transmitted infections excluding HIV | 148724.83 | 189211.24 | 27.22 | 0.01(0 to 0.03) |
| Trinidad and Tobago | Syphilis | 1577.27 | 1685.30 | 6.85 | -0.31(-0.39 to -0.23) |
| Trinidad and Tobago | Chlamydial infection | 55155.18 | 66329.41 | 20.26 | -0.01(-0.02 to -0.01) |
| Trinidad and Tobago | Gonococcal infection | 14845.03 | 14569.45 | -1.86 | -0.22(-0.37 to -0.07) |
| Trinidad and Tobago | Trichomoniasis | 56239.44 | 84387.21 | 50.05 | 0.1(0.07 to 0.13) |
| Trinidad and Tobago | Genital herpes | 20907.90 | 22239.87 | 6.37 | 0(-0.02 to 0.01) |
| Tunisia | Sexually transmitted infections excluding HIV | 726544.23 | 1119592.99 | 54.10 | -0.1(-0.17 to -0.02) |
| Tunisia | Syphilis | 6898.61 | 9982.25 | 44.70 | -0.06(-0.07 to -0.04) |
| Tunisia | Chlamydial infection | 233456.70 | 354184.09 | 51.71 | 0.22(0.08 to 0.36) |
| Tunisia | Gonococcal infection | 139719.53 | 140889.66 | 0.84 | -0.54(-0.68 to -0.41) |
| Tunisia | Trichomoniasis | 278739.63 | 518402.81 | 85.98 | -0.2(-0.33 to -0.06) |
| Tunisia | Genital herpes | 67729.76 | 96134.18 | 41.94 | 0.01(0 to 0.01) |
| Turkey | Sexually transmitted infections excluding HIV | 5320454.00 | 7428188.30 | 39.62 | -0.41(-0.49 to -0.32) |
| Turkey | Syphilis | 29057.43 | 44732.39 | 53.94 | 0.07(-0.12 to 0.26) |
| Turkey | Chlamydial infection | 1984455.38 | 2735342.88 | 37.84 | -0.3(-0.43 to -0.17) |
| Turkey | Gonococcal infection | 1279171.93 | 1323455.93 | 3.46 | -0.33(-0.51 to -0.16) |
| Turkey | Trichomoniasis | 1604132.00 | 2709940.72 | 68.94 | -0.64(-0.74 to -0.54) |
| Turkey | Genital herpes | 423637.26 | 614716.38 | 45.10 | -0.04(-0.22 to 0.14) |
| Turkmenistan | Sexually transmitted infections excluding HIV | 421094.19 | 638328.98 | 51.59 | -0.2(-0.22 to -0.18) |
| Turkmenistan | Syphilis | 1910.53 | 2787.84 | 45.92 | -0.2(-0.24 to -0.15) |
| Turkmenistan | Chlamydial infection | 180971.62 | 281561.03 | 55.58 | -0.11(-0.12 to -0.09) |
| Turkmenistan | Gonococcal infection | 101451.09 | 111242.84 | 9.65 | -0.79(-0.86 to -0.72) |
| Turkmenistan | Trichomoniasis | 112407.40 | 206104.48 | 83.35 | 0(-0.03 to 0.03) |
| Turkmenistan | Genital herpes | 24353.55 | 36632.80 | 50.42 | -0.06(-0.06 to -0.05) |
| Tuvalu | Sexually transmitted infections excluding HIV | 1130.75 | 1439.80 | 27.33 | -0.22(-0.23 to -0.2) |
| Tuvalu | Syphilis | 24.36 | 33.86 | 39.02 | 0.09(0.06 to 0.11) |
| Tuvalu | Chlamydial infection | 284.66 | 334.26 | 17.42 | -0.49(-0.5 to -0.47) |
| Tuvalu | Gonococcal infection | 95.47 | 125.15 | 31.09 | -0.19(-0.22 to -0.17) |
| Tuvalu | Trichomoniasis | 602.42 | 779.83 | 29.45 | -0.17(-0.2 to -0.14) |
| Tuvalu | Genital herpes | 123.85 | 166.70 | 34.60 | 0.03(0.01 to 0.04) |
| Uganda | Sexually transmitted infections excluding HIV | 2498422.27 | 5816823.11 | 132.82 | -0.37(-0.46 to -0.28) |
| Uganda | Syphilis | 95444.85 | 322336.06 | 237.72 | 0.29(0.05 to 0.53) |
| Uganda | Chlamydial infection | 489586.64 | 1106798.01 | 126.07 | -0.46(-0.55 to -0.37) |
| Uganda | Gonococcal infection | 464687.24 | 1187991.34 | 155.65 | -0.18(-0.47 to 0.11) |
| Uganda | Trichomoniasis | 880911.61 | 2195516.18 | 149.23 | -0.11(-0.19 to -0.03) |
| Uganda | Genital herpes | 567791.93 | 1004181.51 | 76.86 | -1.39(-1.58 to -1.2) |
| Ukraine | Sexually transmitted infections excluding HIV | 5357856.71 | 4587571.94 | -14.38 | -0.13(-0.16 to -0.1) |
| Ukraine | Syphilis | 34221.49 | 19970.56 | -41.64 | -1.89(-2.21 to -1.57) |
| Ukraine | Chlamydial infection | 1946569.84 | 1714418.13 | -11.93 | 0(-0.02 to 0.01) |
| Ukraine | Gonococcal infection | 1083748.20 | 747234.18 | -31.05 | -0.61(-0.73 to -0.5) |
| Ukraine | Trichomoniasis | 1811128.85 | 1713704.27 | -5.38 | 0.05(0.02 to 0.08) |
| Ukraine | Genital herpes | 482188.33 | 392244.80 | -18.65 | 0(-0.01 to 0) |
| United Arab Emirates | Sexually transmitted infections excluding HIV | 199860.82 | 1303251.91 | 552.08 | -0.01(-0.03 to 0) |
| United Arab Emirates | Syphilis | 2241.52 | 12630.14 | 463.46 | -0.03(-0.12 to 0.06) |
| United Arab Emirates | Chlamydial infection | 74151.85 | 453273.50 | 511.28 | 0.03(0.01 to 0.06) |
| United Arab Emirates | Gonococcal infection | 24603.04 | 100587.08 | 308.84 | -0.12(-0.18 to -0.06) |
| United Arab Emirates | Trichomoniasis | 82834.46 | 652496.20 | 687.71 | -0.01(-0.03 to 0.02) |
| United Arab Emirates | Genital herpes | 16029.94 | 84264.98 | 425.67 | -0.07(-0.13 to 0) |
| United Kingdom | Sexually transmitted infections excluding HIV | 2194556.82 | 2582683.46 | 17.69 | -0.04(-0.07 to -0.02) |
| United Kingdom | Syphilis | 42822.55 | 46177.55 | 7.83 | 0.07(0.02 to 0.12) |
| United Kingdom | Chlamydial infection | 176197.65 | 200823.19 | 13.98 | -0.51(-0.69 to -0.34) |
| United Kingdom | Gonococcal infection | 78928.65 | 87047.53 | 10.29 | -0.84(-1.18 to -0.51) |
| United Kingdom | Trichomoniasis | 1548915.68 | 1857980.87 | 19.95 | 0.03(0.02 to 0.03) |
| United Kingdom | Genital herpes | 347692.28 | 390654.32 | 12.36 | 0.04(0.03 to 0.06) |
| United Republic of Tanzania | Sexually transmitted infections excluding HIV | 4056629.36 | 8881370.31 | 118.93 | -0.22(-0.25 to -0.19) |
| United Republic of Tanzania | Syphilis | 253579.44 | 294104.10 | 15.98 | -2.83(-3.21 to -2.45) |
| United Republic of Tanzania | Chlamydial infection | 755249.29 | 1644973.91 | 117.81 | -0.11(-0.32 to 0.1) |
| United Republic of Tanzania | Gonococcal infection | 441164.89 | 890892.44 | 101.94 | -0.29(-0.49 to -0.09) |
| United Republic of Tanzania | Trichomoniasis | 2078237.81 | 4939751.50 | 137.69 | -0.15(-0.22 to -0.08) |
| United Republic of Tanzania | Genital herpes | 528397.93 | 1111648.36 | 110.38 | 0.24(0.08 to 0.39) |
| United States of America | Sexually transmitted infections excluding HIV | 18919987.20 | 22426037.91 | 18.53 | -0.26(-0.31 to -0.21) |
| United States of America | Syphilis | 189596.30 | 234505.92 | 23.69 | 0.2(-0.04 to 0.44) |
| United States of America | Chlamydial infection | 2024190.20 | 2522834.69 | 24.63 | -0.64(-0.98 to -0.29) |
| United States of America | Gonococcal infection | 984559.83 | 1177835.07 | 19.63 | -0.3(-0.45 to -0.14) |
| United States of America | Trichomoniasis | 12305650.45 | 15098749.30 | 22.70 | -0.1(-0.13 to -0.08) |
| United States of America | Genital herpes | 3415990.41 | 3392112.92 | -0.70 | -0.6(-0.71 to -0.49) |
| United States Virgin Islands | Sexually transmitted infections excluding HIV | 13614.01 | 12427.81 | -8.71 | 0.01(-0.01 to 0.02) |
| United States Virgin Islands | Syphilis | 167.97 | 139.04 | -17.22 | -0.01(-0.04 to 0.01) |
| United States Virgin Islands | Chlamydial infection | 4828.59 | 4076.17 | -15.58 | -0.03(-0.04 to -0.02) |
| United States Virgin Islands | Gonococcal infection | 1333.50 | 1019.77 | -23.53 | -0.23(-0.3 to -0.15) |
| United States Virgin Islands | Trichomoniasis | 5538.63 | 5800.66 | 4.73 | 0.09(0.06 to 0.12) |
| United States Virgin Islands | Genital herpes | 1745.32 | 1392.17 | -20.23 | 0.01(-0.02 to 0.03) |
| Uruguay | Sexually transmitted infections excluding HIV | 173727.47 | 197677.24 | 13.79 | -0.01(-0.02 to 0.01) |
| Uruguay | Syphilis | 3863.63 | 3858.44 | -0.13 | 0.03(-0.1 to 0.16) |
| Uruguay | Chlamydial infection | 27225.27 | 31030.15 | 13.98 | 0(-0.01 to 0) |
| Uruguay | Gonococcal infection | 13866.76 | 13985.01 | 0.85 | -0.28(-0.3 to -0.26) |
| Uruguay | Trichomoniasis | 92178.90 | 109128.41 | 18.39 | 0.02(0 to 0.04) |
| Uruguay | Genital herpes | 36592.92 | 39675.24 | 8.42 | 0.02(0.01 to 0.03) |
| Uzbekistan | Sexually transmitted infections excluding HIV | 2345049.20 | 4404185.53 | 87.81 | -0.12(-0.14 to -0.09) |
| Uzbekistan | Syphilis | 10936.45 | 18499.13 | 69.15 | -0.55(-0.63 to -0.47) |
| Uzbekistan | Chlamydial infection | 1002595.44 | 1948318.97 | 94.33 | -0.02(-0.03 to -0.01) |
| Uzbekistan | Gonococcal infection | 567157.52 | 819801.31 | 44.55 | -0.59(-0.67 to -0.51) |
| Uzbekistan | Trichomoniasis | 628926.38 | 1364278.00 | 116.92 | 0.02(-0.01 to 0.05) |
| Uzbekistan | Genital herpes | 135433.40 | 253288.13 | 87.02 | 0(0 to 0.01) |
| Vanuatu | Sexually transmitted infections excluding HIV | 17847.30 | 36417.52 | 104.05 | -0.15(-0.18 to -0.12) |
| Vanuatu | Syphilis | 443.00 | 911.50 | 105.76 | 0(-0.03 to 0.03) |
| Vanuatu | Chlamydial infection | 5905.01 | 10642.11 | 80.22 | -0.53(-0.61 to -0.45) |
| Vanuatu | Gonococcal infection | 1938.84 | 3727.35 | 92.25 | -0.21(-0.3 to -0.13) |
| Vanuatu | Trichomoniasis | 7738.18 | 17390.97 | 124.74 | 0.05(0.02 to 0.08) |
| Vanuatu | Genital herpes | 1822.26 | 3745.59 | 105.55 | 0.01(-0.02 to 0.04) |
| Venezuela (Bolivarian Republic of) | Sexually transmitted infections excluding HIV | 2182768.35 | 3717594.24 | 70.32 | 0.04(0.01 to 0.06) |
| Venezuela (Bolivarian Republic of) | Syphilis | 33014.50 | 52914.28 | 60.28 | 0.42(0.34 to 0.5) |
| Venezuela (Bolivarian Republic of) | Chlamydial infection | 686458.96 | 1072249.23 | 56.20 | -0.04(-0.06 to -0.03) |
| Venezuela (Bolivarian Republic of) | Gonococcal infection | 158113.05 | 206889.30 | 30.85 | -0.19(-0.28 to -0.1) |
| Venezuela (Bolivarian Republic of) | Trichomoniasis | 968811.80 | 1914016.35 | 97.56 | 0.11(0.06 to 0.15) |
| Venezuela (Bolivarian Republic of) | Genital herpes | 336370.04 | 471525.08 | 40.18 | 0.01(0 to 0.02) |
| Viet Nam | Sexually transmitted infections excluding HIV | 6100075.87 | 11221775.77 | 83.96 | 0.08(0.06 to 0.1) |
| Viet Nam | Syphilis | 51713.66 | 84541.13 | 63.48 | 0.09(0.06 to 0.12) |
| Viet Nam | Chlamydial infection | 2407767.82 | 4432175.15 | 84.08 | 0.2(0.17 to 0.23) |
| Viet Nam | Gonococcal infection | 806079.31 | 1147142.15 | 42.31 | -0.18(-0.25 to -0.1) |
| Viet Nam | Trichomoniasis | 2064314.03 | 4388966.77 | 112.61 | 0.05(0.01 to 0.09) |
| Viet Nam | Genital herpes | 770201.05 | 1168950.56 | 51.77 | 0.04(0.02 to 0.05) |
| Yemen | Sexually transmitted infections excluding HIV | 851579.43 | 2412047.91 | 183.24 | -0.05(-0.06 to -0.05) |
| Yemen | Syphilis | 19058.54 | 53433.95 | 180.37 | -0.46(-0.62 to -0.29) |
| Yemen | Chlamydial infection | 298531.52 | 862687.44 | 188.98 | -0.01(-0.03 to 0.01) |
| Yemen | Gonococcal infection | 140450.24 | 368974.80 | 162.71 | -0.15(-0.17 to -0.13) |
| Yemen | Trichomoniasis | 302233.33 | 873867.00 | 189.14 | -0.05(-0.07 to -0.03) |
| Yemen | Genital herpes | 91305.80 | 253084.72 | 177.18 | -0.01(-0.01 to 0) |
| Zambia | Sexually transmitted infections excluding HIV | 1095742.89 | 2805906.30 | 156.07 | -0.12(-0.17 to -0.07) |
| Zambia | Syphilis | 63740.36 | 134911.02 | 111.66 | -1.12(-1.46 to -0.79) |
| Zambia | Chlamydial infection | 158957.50 | 421120.42 | 164.93 | 0.09(0.01 to 0.18) |
| Zambia | Gonococcal infection | 111023.35 | 255579.78 | 130.20 | -0.14(-0.19 to -0.1) |
| Zambia | Trichomoniasis | 572409.05 | 1524307.50 | 166.30 | -0.06(-0.11 to -0.02) |
| Zambia | Genital herpes | 189612.64 | 469987.58 | 147.87 | -0.19(-0.45 to 0.08) |
| Zimbabwe | Sexually transmitted infections excluding HIV | 1242805.02 | 2059887.12 | 65.74 | -0.15(-0.21 to -0.09) |
| Zimbabwe | Syphilis | 41664.17 | 63116.98 | 51.49 | -0.2(-0.24 to -0.15) |
| Zimbabwe | Chlamydial infection | 183673.82 | 304154.73 | 65.60 | -0.03(-0.05 to -0.02) |
| Zimbabwe | Gonococcal infection | 233061.60 | 336830.57 | 44.52 | -0.13(-0.18 to -0.07) |
| Zimbabwe | Trichomoniasis | 548593.36 | 968153.09 | 76.48 | -0.22(-0.32 to -0.13) |
| Zimbabwe | Genital herpes | 235812.07 | 387631.75 | 64.38 | -0.02(-0.14 to 0.1) |

**Fig S1. The age standardized incidence of syphilis in 2019.**


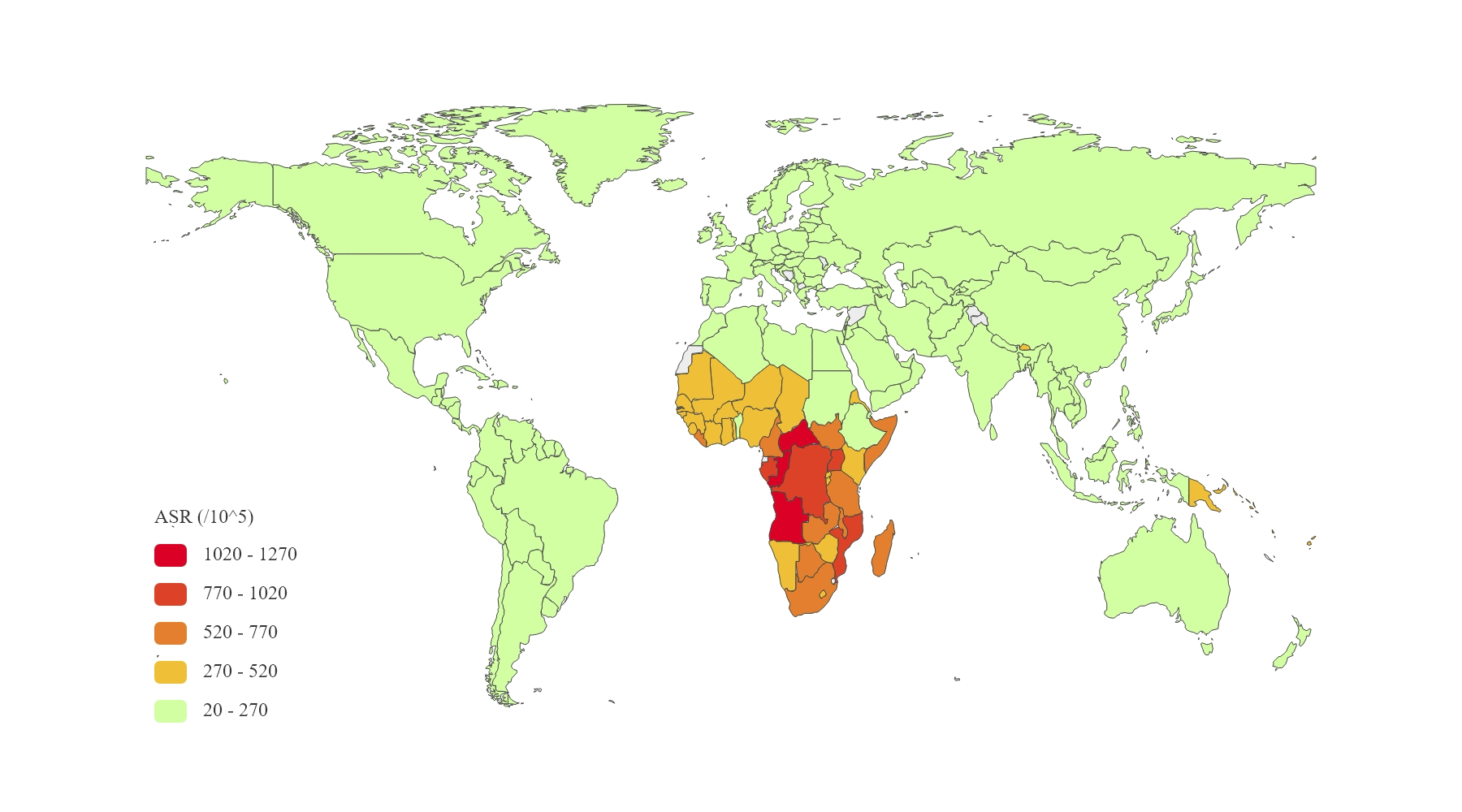


**Fig S2. The percentage change in absolute number of syphilis between 1990 and 2019.**

**
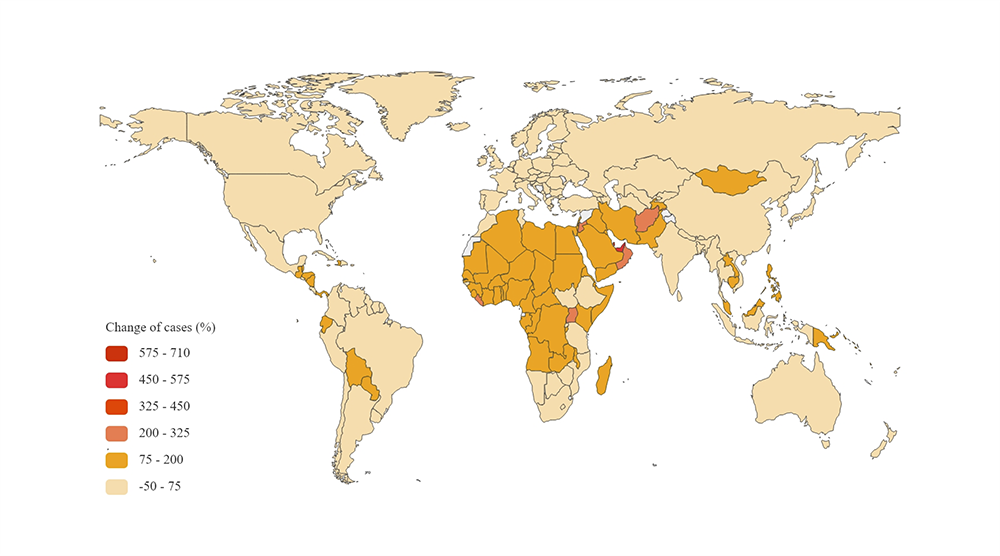
**

**Fig S3. The estimated annual percentage change in ASR of syphilis from 1990 to 2019.**

**
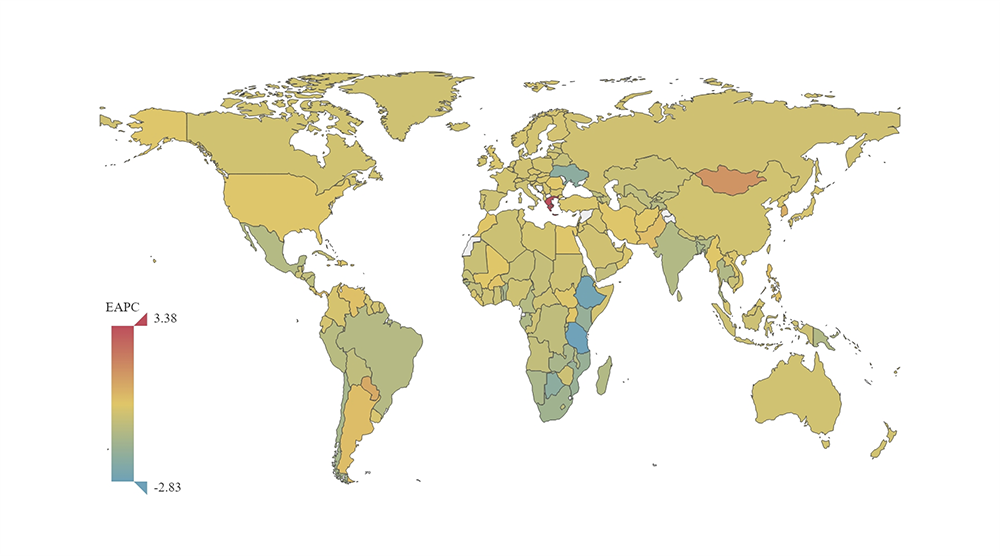
**

**Fig S4. The age standardized incidence of chlamydia in 2019.**


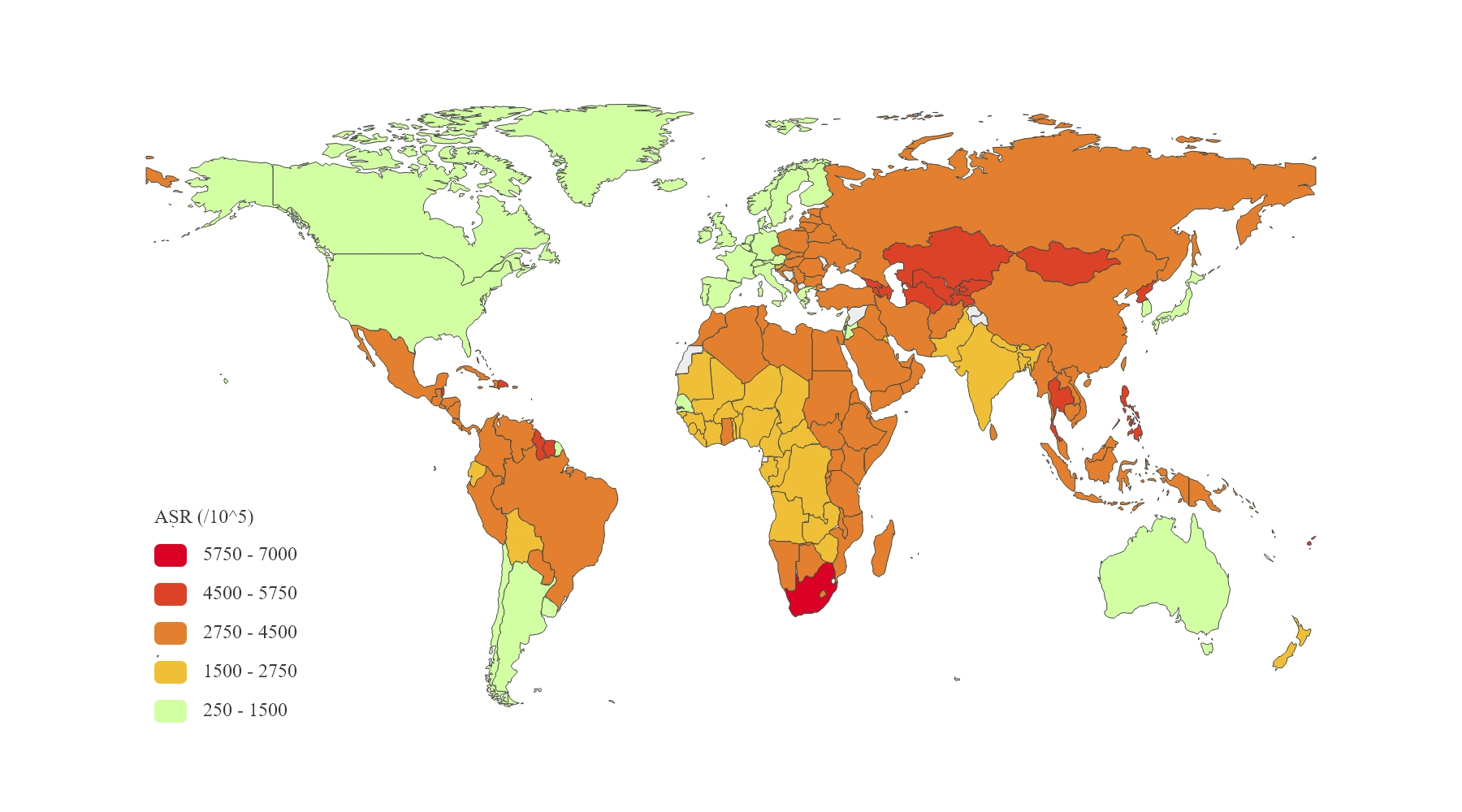


**Fig S5. The percentage change in absolute number of chlamydia between 1990 and 2019.**

**
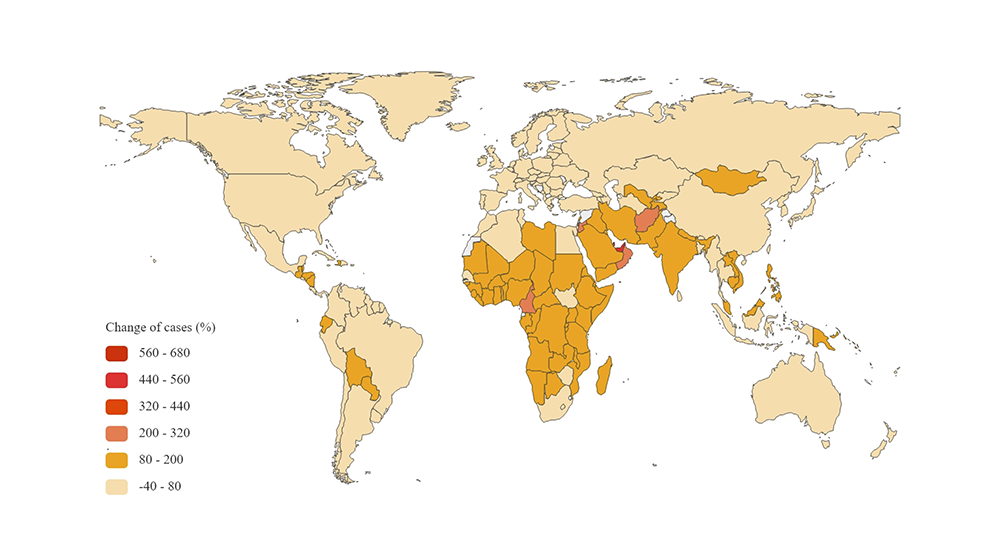
**

**Fig S6. The estimated annual percentage change in ASR of chlamydia from 1990 to 2019.**

**
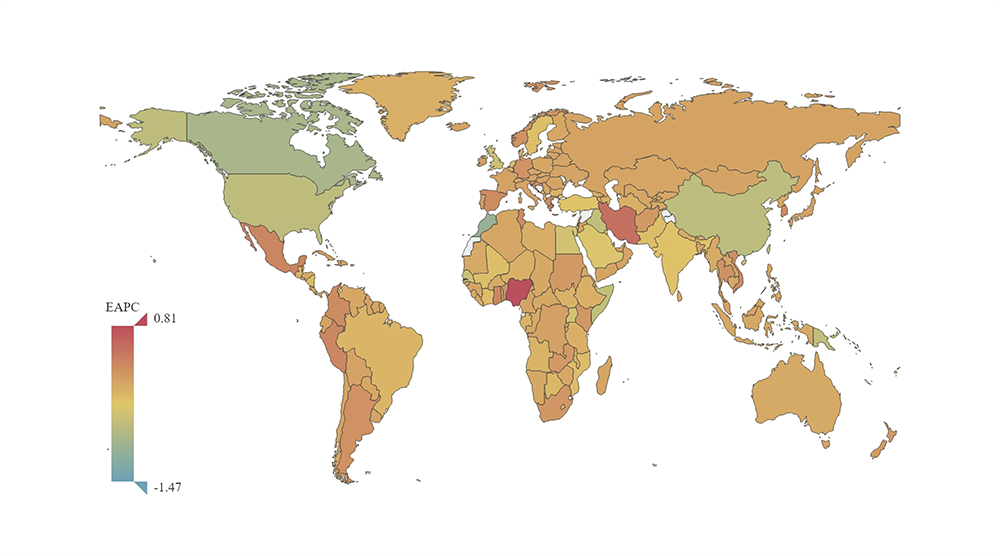
**

**Fig S7. The age standardized incidence of gonorrhoea in 2019.**


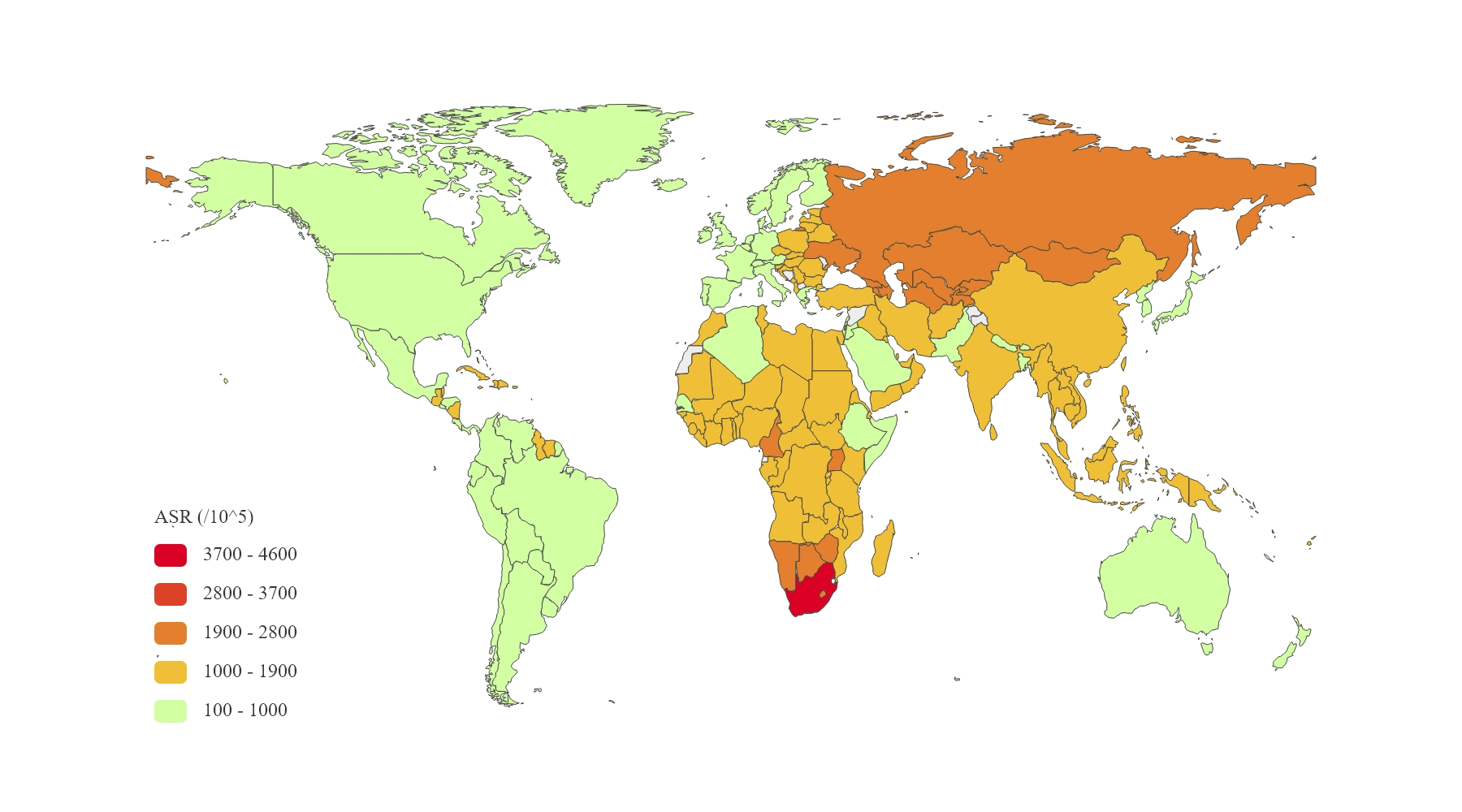


**Fig S8. The percentage change in absolute number of gonorrhoea between 1990 and 2019.**

**
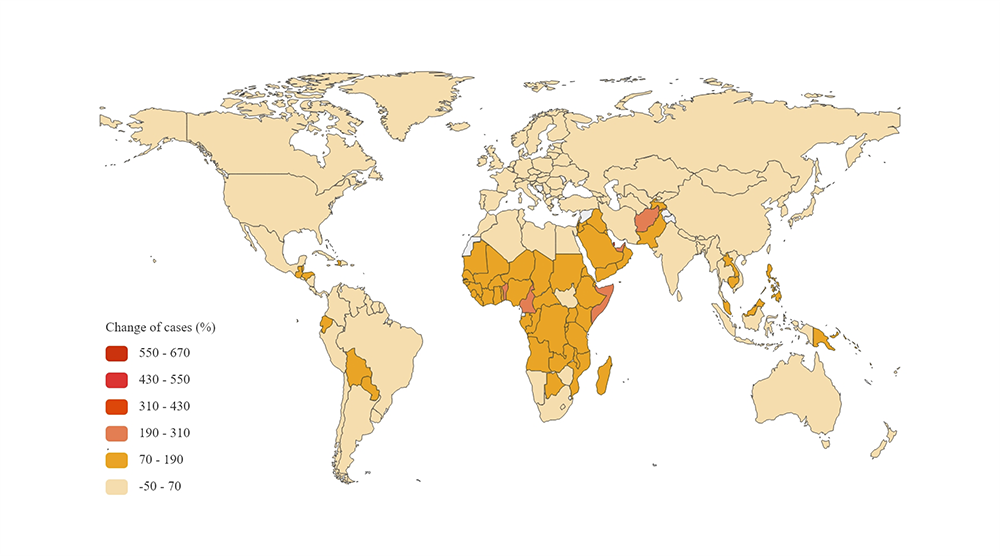
**

**Fig S9. The estimated annual percentage change in ASR of gonorrhoea from 1990 to 2019.**

**
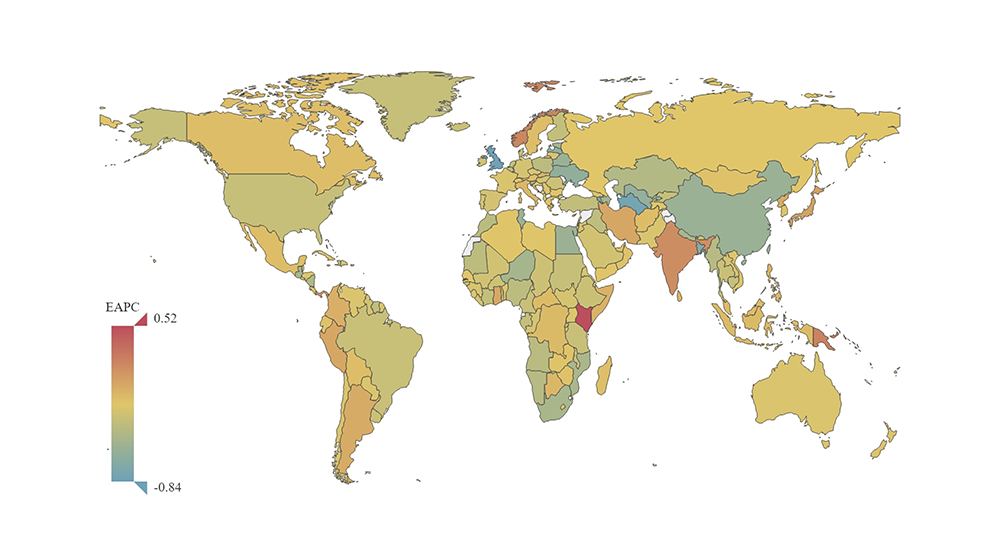
**

**Fig S10. The age standardized incidence of trichomoniasis in 2019.**


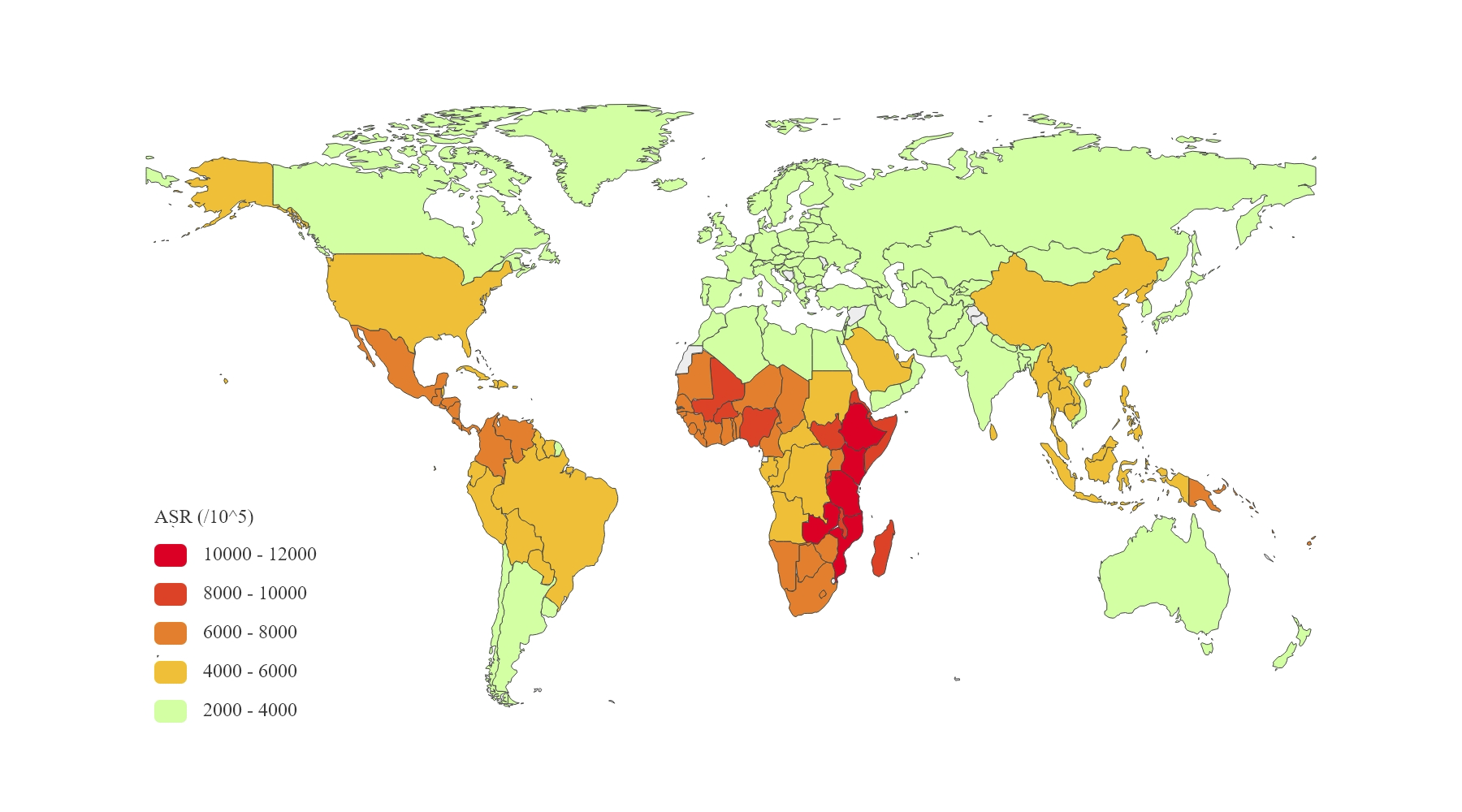


**Fig S11. The percentage change in absolute number of trichomoniasis between 1990 and 2019.**

**
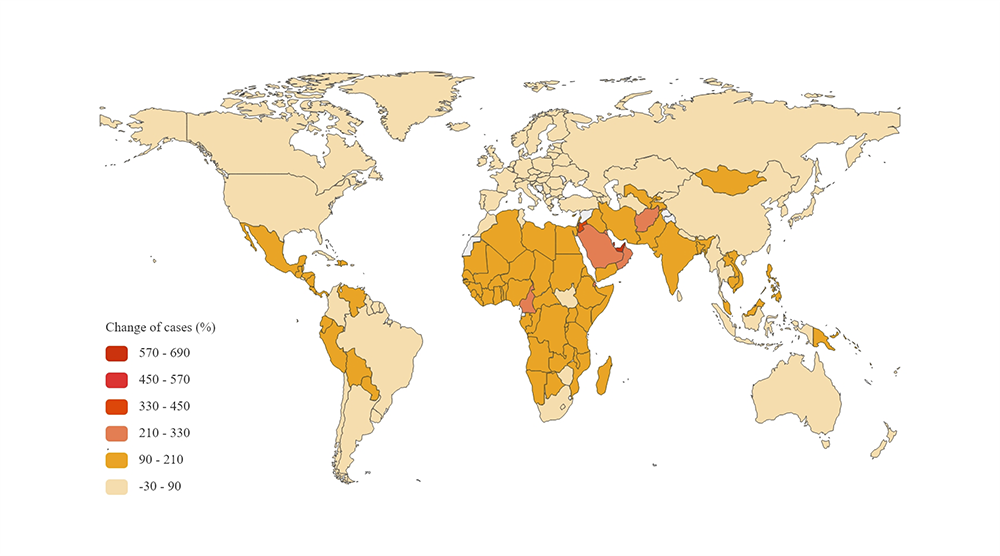
**

**Fig S12. The estimated annual percentage change in ASR of trichomoniasis from 1990 to 2019.**

**
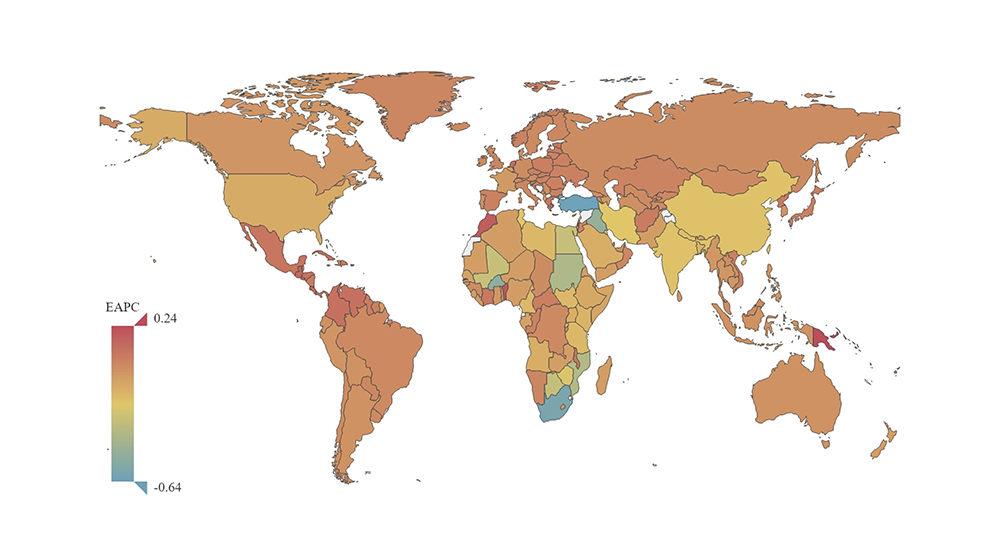
**

**Fig S13. The age standardized incidence of genital herpes in 2019.**


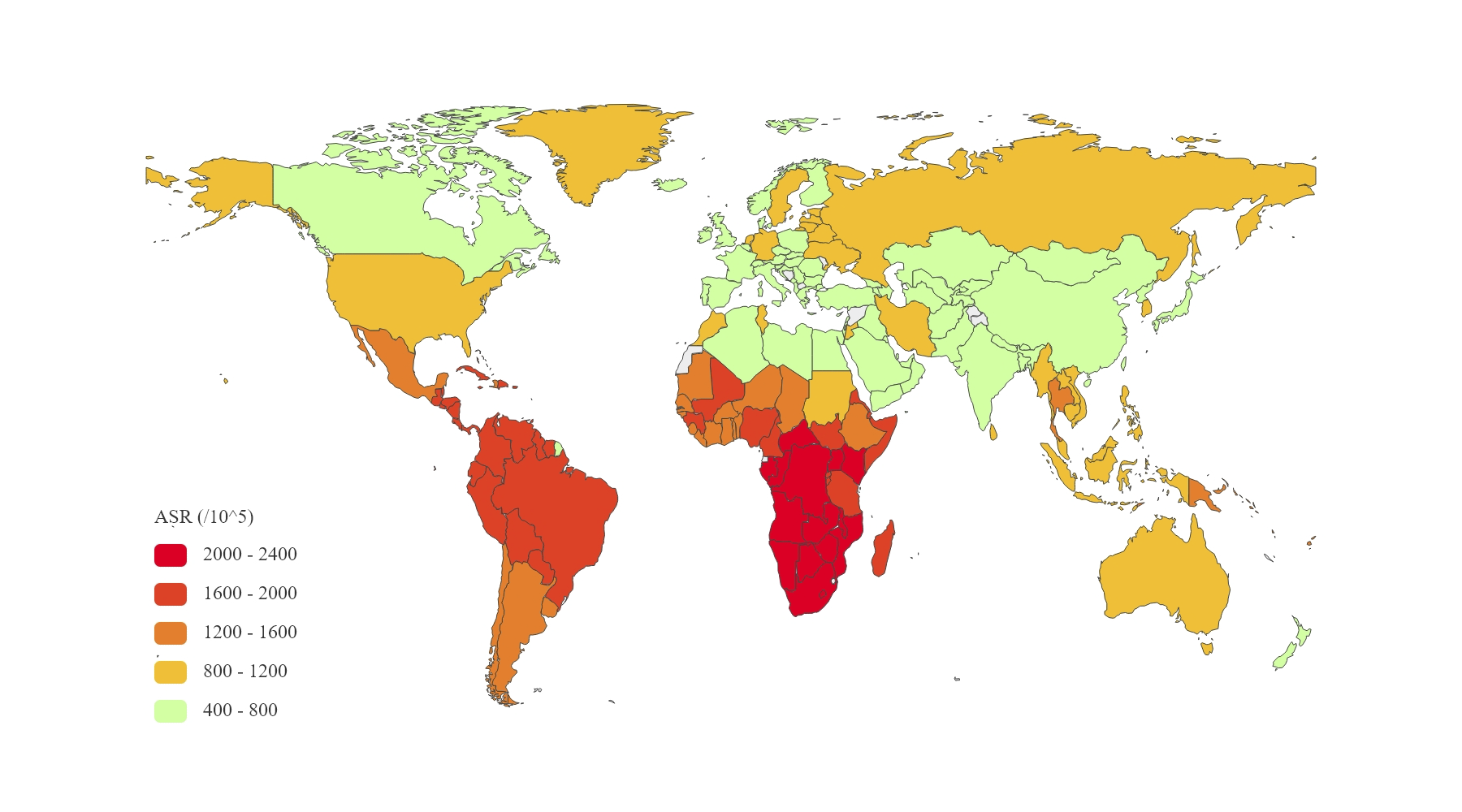


**Fig S14. The percentage change in absolute number of genital herpes between 1990 and 2019.**

**
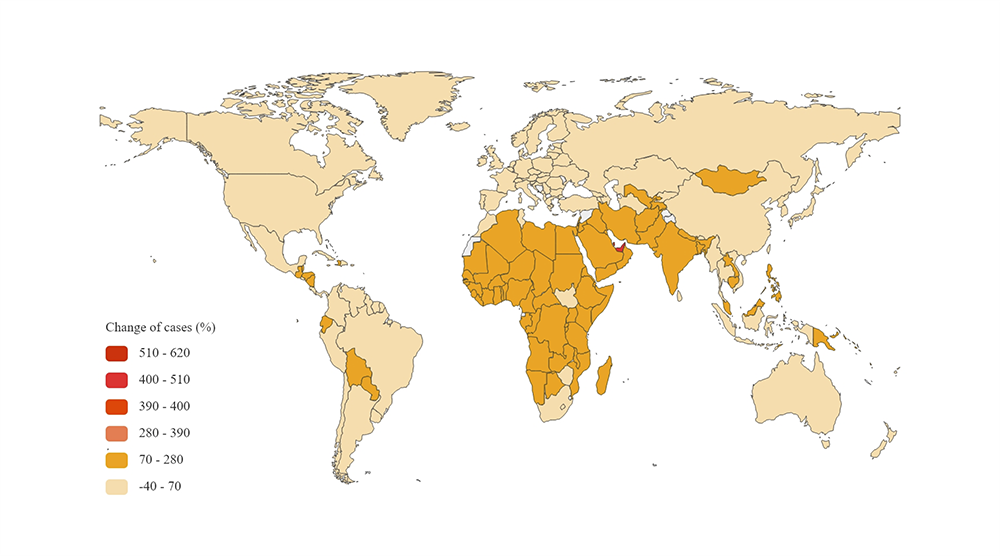
**

**Fig S15. The estimated annual percentage change in ASR of genital herpes from 1990 to 2019.**

**
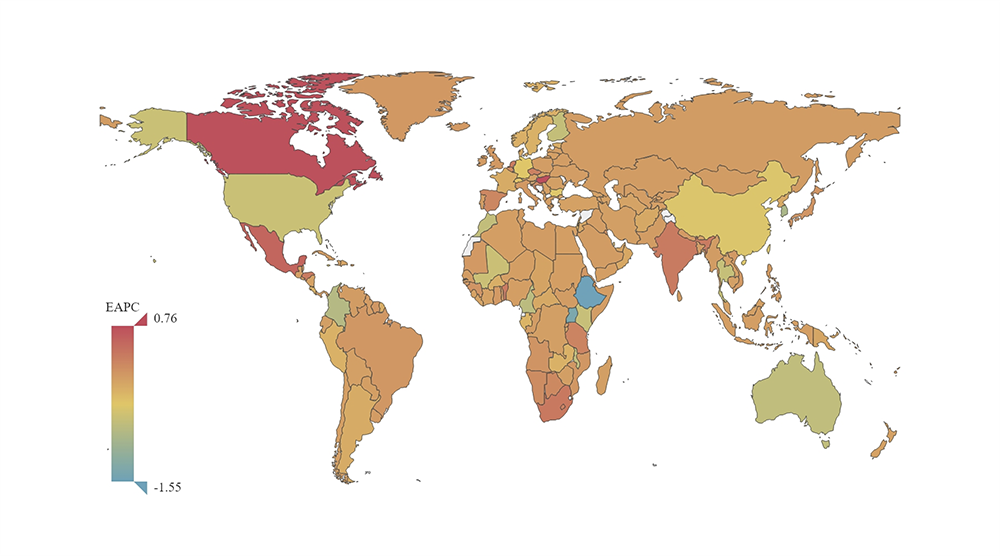
**

**Fig S16. The correlation between EAPC and ASR in 1990 (A), and HDI in 2019 (B) for syphilis. The incident cases from countries was represented by the circles and its’ size.** (Abbreviations: EAPC, the estimated average percentage change; ASR, the age-standardized rate; HDI, human development index).


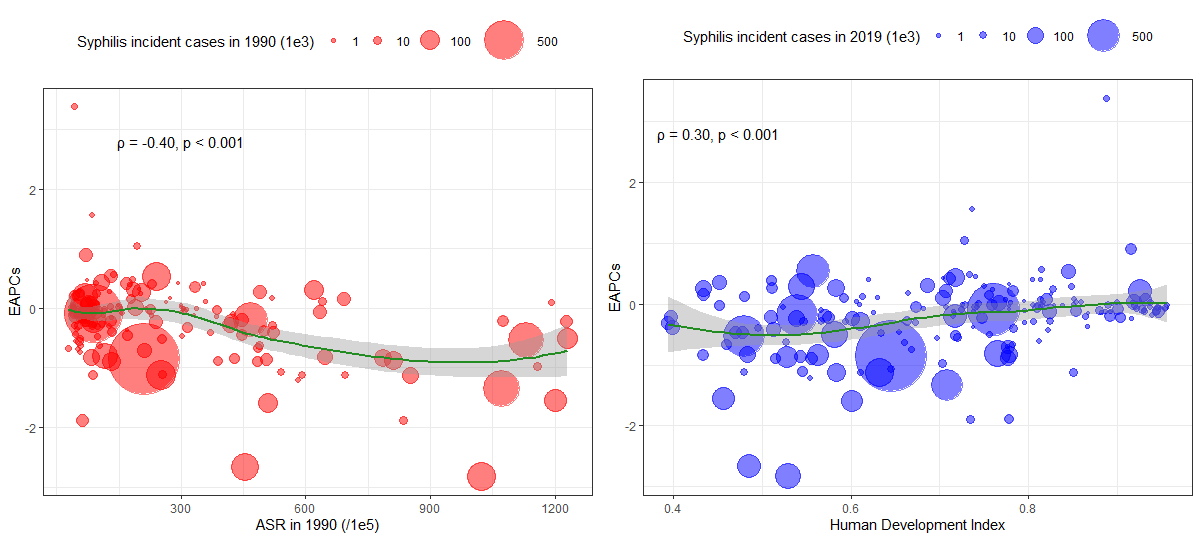


**Fig S17. The correlation between EAPC and ASR in 1990 (A), and HDI in 2019 (B) for chlamydia. The incident cases from countries was represented by the circles and its’ size.** (Abbreviations: EAPC, the estimated average percentage change; ASR, the age-standardized rate; HDI, human development index).


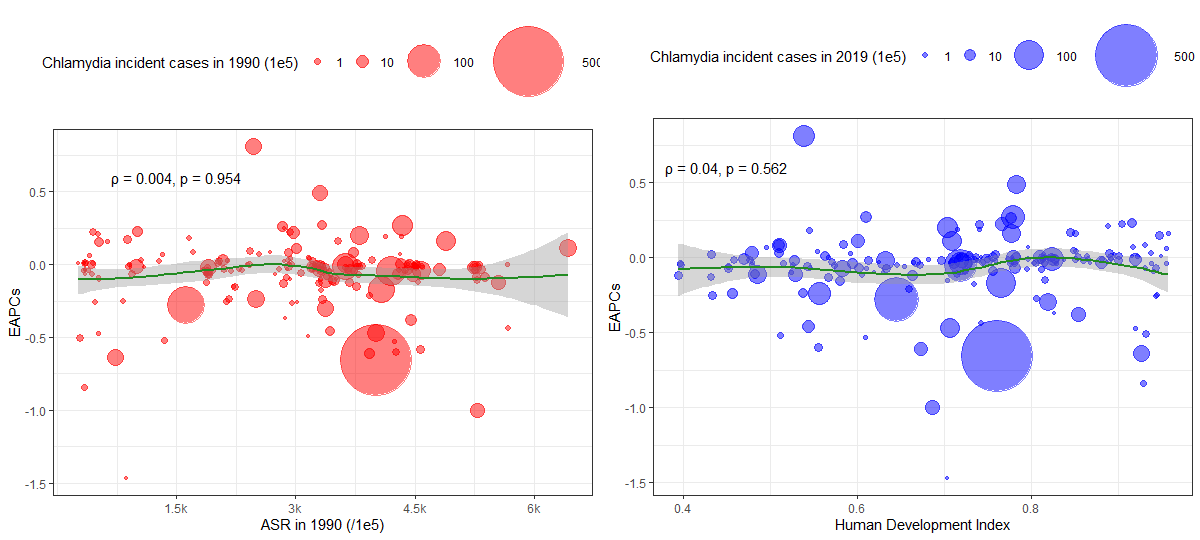


**Fig S18. The correlation between EAPC and ASR in 1990 (A), and HDI in 2019 (B) for gonorrhoea. The incident cases from countries was represented by the circles and its’ size.** (Abbreviations: EAPC, the estimated average percentage change; ASR, the age-standardized rate; HDI, human development index).


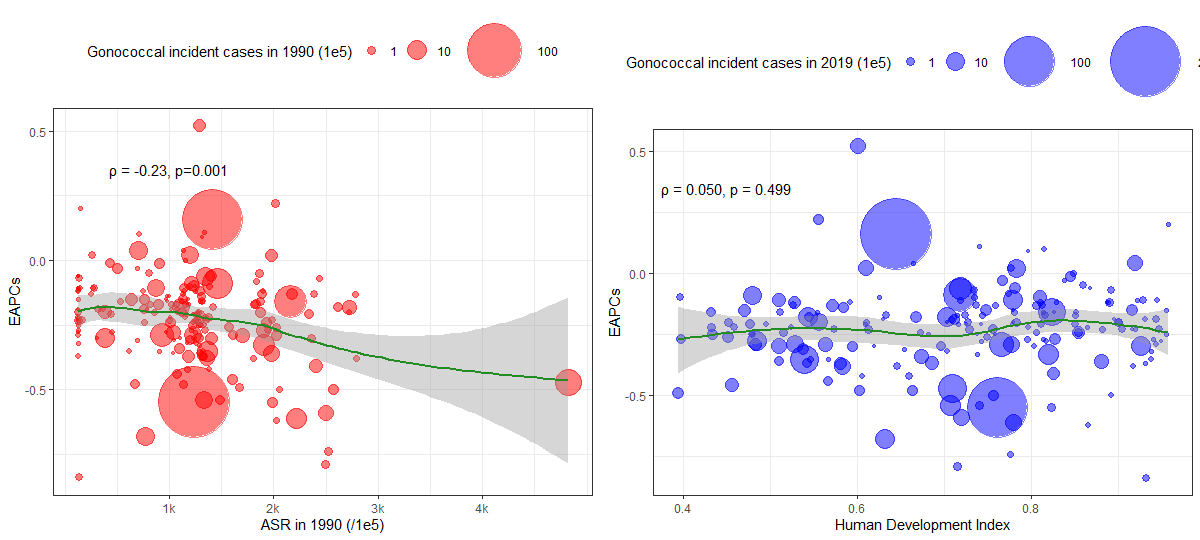


**Fig S19. The correlation between EAPC and ASR in 1990 (A), and HDI in 2019 (B) for trichomoniasis. The incident cases from countries was represented by the circles and its’ size.** (Abbreviations: EAPC, the estimated average percentage change; ASR, the age-standardized rate; HDI, human development index).


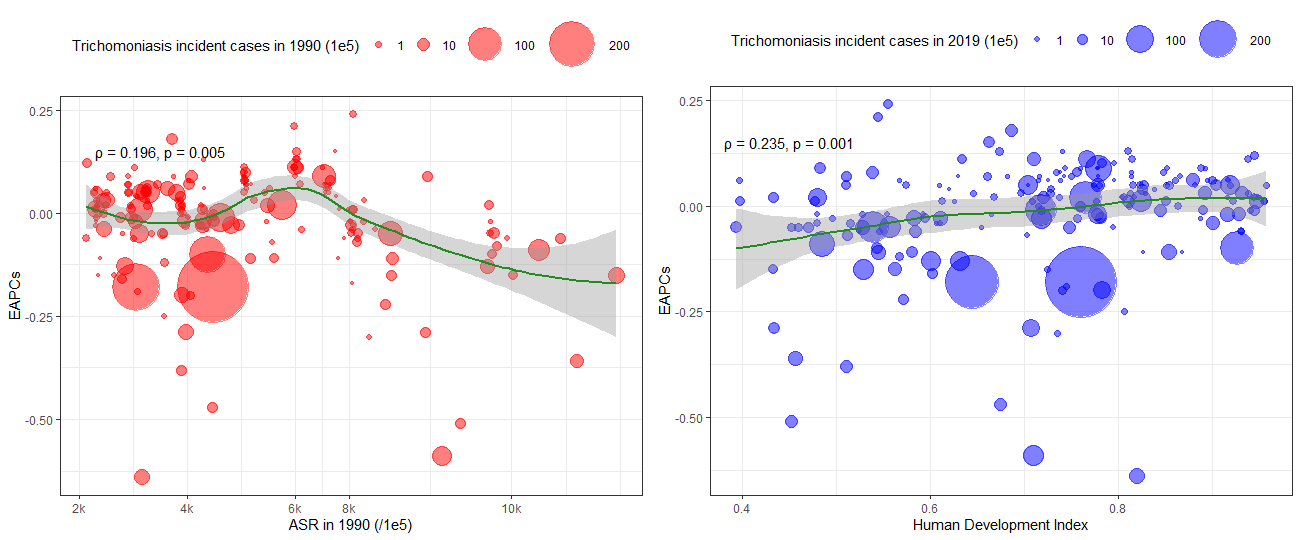


**Fig S20. The correlation between EAPC and ASR in 1990 (A), and HDI in 2019 (B) for genital herpes. The incident cases from countries was represented by the circles and its’ size.** (Abbreviations: EAPC, the estimated average percentage change; ASR, the age-standardized rate; HDI, human development index).


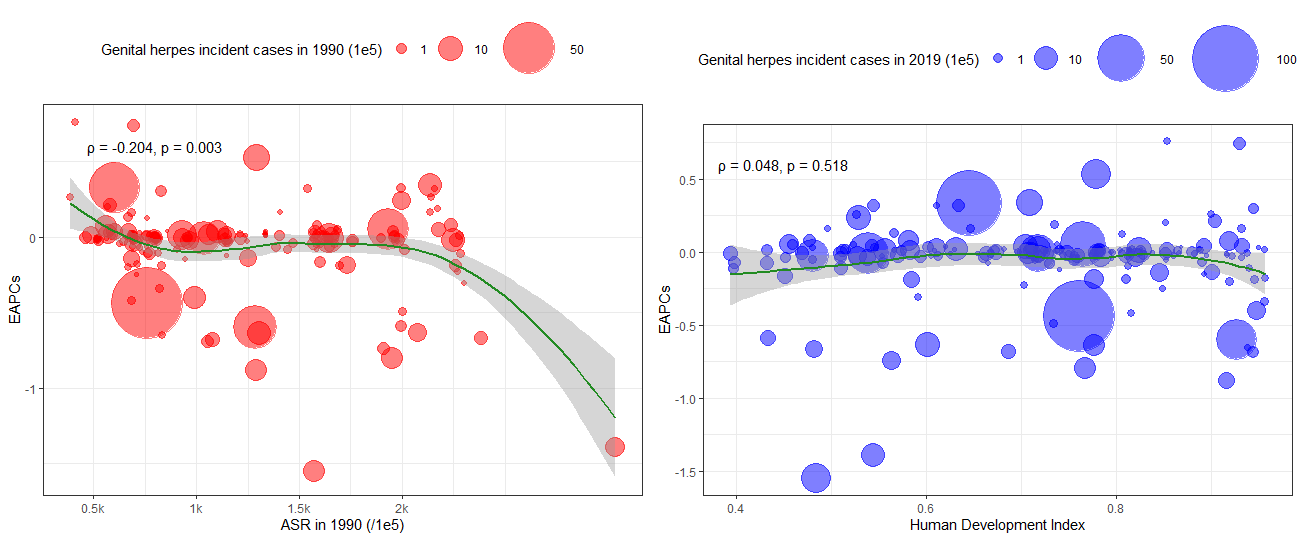

Supplement: Supplementary file 1 [file Data_Sheet_1.doc]
